# MicroRNA expression profiling of human breast cancer identifies new markers of tumor subtype

**DOI:** 10.1186/gb-2007-8-10-r214

**Published:** 2007-10-08

**Authors:** Cherie Blenkiron, Leonard D Goldstein, Natalie P Thorne, Inmaculada Spiteri, Suet-Feung Chin, Mark J Dunning, Nuno L Barbosa-Morais, Andrew E Teschendorff, Andrew R Green, Ian O Ellis, Simon Tavaré, Carlos Caldas, Eric A Miska

**Affiliations:** 1 Cancer Research UK, Cambridge Research Institute, Li Ka-Shing Centre, Robinson Way, Cambridge CB2 0RE, UK; 2 Department of Oncology, University of Cambridge, Hills Road, Cambridge CB2 2XZ, UK; 3 Wellcome Trust/Cancer Research UK Gurdon Institute, University of Cambridge, The Henry Wellcome Building of Cancer and Developmental Biology, Tennis Court Rd, Cambridge CB2 1QN, UK; 4 Department of Biochemistry, University of Cambridge, Tennis Court Rd, Cambridge CB2 1GA, UK; 5 Department of Applied Mathematics and Theoretical Physics, University of Cambridge, Centre for Mathematical Sciences, Wilberforce Road, Cambridge CB3 0WA, UK; 6 Department of Histopathology, School of Molecular Medical Sciences, University of Nottingham, Nottingham NG5 1PB, UK

## Abstract

**Background:**

MicroRNAs (miRNAs), a class of short non-coding RNAs found in many plants and animals, often act post-transcriptionally to inhibit gene expression.

**Results:**

Here we report the analysis of miRNA expression in 93 primary human breast tumors, using a bead-based flow cytometric miRNA expression profiling method. Of 309 human miRNAs assayed, we identify 133 miRNAs expressed in human breast and breast tumors. We used mRNA expression profiling to classify the breast tumors as luminal A, luminal B, basal-like, HER2+ and normal-like. A number of miRNAs are differentially expressed between these molecular tumor subtypes and individual miRNAs are associated with clinicopathological factors. Furthermore, we find that miRNAs could classify basal versus luminal tumor subtypes in an independent data set. In some cases, changes in miRNA expression correlate with genomic loss or gain; in others, changes in miRNA expression are likely due to changes in primary transcription and or miRNA biogenesis. Finally, the expression of *DICER1 *and *AGO2 *is correlated with tumor subtype and may explain some of the changes in miRNA expression observed.

**Conclusion:**

This study represents the first integrated analysis of miRNA expression, mRNA expression and genomic changes in human breast cancer and may serve as a basis for functional studies of the role of miRNAs in the etiology of breast cancer. Furthermore, we demonstrate that bead-based flow cytometric miRNA expression profiling might be a suitable platform to classify breast cancer into prognostic molecular subtypes.

## Abstract 2

Integrated analysis of miRNA expression and genomic changes in human breast tumors allows the classification of tumor subtypes.

## Background

MicroRNAs (miRNAs) were discovered in *Caenorhabditis elegans *during studies of the control of developmental timing [1-5]. miRNAs are approximately 22-nucleotide non-coding RNAs that are thought to regulate gene expression through sequence-specific base-pairing with target mRNAs [6]. To date, thousands of miRNAs have been identified in organisms as diverse as roundworms, flies, fish, frogs, mammals, flowering plants, mosses, and even viruses, using genetics, molecular cloning and predictions from bioinformatics [7-16]. The human genome encodes at least 474 miRNA genes [17,18].

miRNAs are transcribed as long RNA precursors (pri-miRNAs), which are processed in the nucleus by the RNase III enzyme complex Drosha-Pasha/DGCR8 to form the approximately 70-base pre-miRNAs [19-23]. Pre-miRNAs are exported from the nucleus by Exportin-5 [24], processed by the RNase III enzyme Dicer, and incorporated into an Argonaute-containing RNA-induced silencing complex (RISC) [25]. Within the silencing complex, miRNAs pair to the messages of protein-coding genes, usually through imperfect base-pairing with the 3'-untranslated region (3'UTR), thereby specifying the post-transcriptional repression of these target mRNAs [6,26]. Binding of the silencing complex causes translational repression [27-29] and/or mRNA destabilization, which is sometimes through direct mRNA cleavage [30,31] but usually through other mechanisms [32-36].

The function of human miRNAs is largely unknown. However, studies in roundworms, flies, fish and mice have demonstrated important roles for miRNAs in animal development [37]. miRNA target predictions suggest important roles for miRNAs in humans. Because many mRNAs have been under selective pressure to preserve pairing to a six nucleotide sequence in the 5' region of the miRNA known as the miRNA seed (nucleotides 2-7), targets of metazoan miRNAs can be predicted by searching for conserved matches to the seed region [38-42]. In humans, at least 10% of the protein-coding mRNAs might be conserved targets of miRNAs [38,39,41-49].

Despite their recent discovery, strong links between miRNAs and human cancer are emerging. Initial observations in roundworms and flies suggested possible connections between miRNAs and proliferation defects [50]. More recently, it was shown that the human miRNAs miR-15a and miR-16-1 map to a region on 13q14 that is often deleted in B-cell chronic lymphocytic leukemias (CLL) and that miR-15a and miR-16-1 are frequently deregulated in CLL patients [51]. A second study found that miR-143 and miR-145 expression levels were reduced in adenomatous and cancer stages of colorectal neoplasia [52]. Subsequently, a number of studies, using a range of techniques, including miRNA cloning, quantitative PCR, microarrays and bead-based flow cytometric miRNA expression profiling [53-56], demonstrated that miRNA expression is deregulated in many human cancers.

A number of miRNAs were found to have oncogenic potential. For example, the *mir-17 *miRNA cluster cooperates with the oncogene *Myc *to induce tumors in a mouse model [57] and miR-372 and miR-373 were found to cooperate with *RAS *in an *in vitro *assay [58]. miRNAs might also act as tumor suppressors. For example, deregulation of the oncogene *RAS *and *HMGA2 *by loss of regulation through the *let-7 *family of miRNAs might contribute to human cancer [59-61]. It is unclear how miRNAs might be deregulated in cancer; however, it has been observed that many human miRNAs lie within cancer associated genomic regions, that is, areas of loss, gain or rearrangement of the DNA in tumors [62]. However, transcriptional or post-transcriptional regulation of miRNAs in cancer has also been proposed [63,64].

The molecular classification of human tumors using mRNA microarray profiling is an area of intense research. A number of classifiers have been developed for human breast tumors, including the use of expression signatures as prognostic tools [65-75]. One of these classifiers can be used as a single sample predictor (SSP) to assign individual samples to one of five breast tumor subtypes: luminal A, luminal B, basal-like, HER2+ and normal breast-like [65,69,70,76].

Two recent studies have shown that a number of miRNAs are deregulated in human breast cancer [77,78]. A third study found that a number of miRNAs were differentially expressed in breast tumor biopsies and that miRNA expression correlated with HER2 and estrogen receptor (ER) status [79].

This study represents the first integrated analysis of miRNA expression, mRNA expression and genomic changes in human breast cancer and may serve as a basis for functional studies of the role of miRNAs in the etiology of breast cancer. Furthermore, we demonstrate that bead-based flow cytometric miRNA expression profiling might be a suitable platform to classify breast cancer into prognostic molecular subtypes. This potential will need to be addressed in a prospective study.

## Results

### There are 133 miRNAs expressed in normal human breast and primary human breast cancer

To generate a comprehensive set of miRNA expression profiles for primary human breast cancer we selected 99 primary human tumors, 5 normal breast samples and 33 breast cancer cell lines for miRNA expression profiling. Tumor samples were fresh-frozen and collected from Nottingham City Hospital Tumor Bank and are representative with regard to tumor subtypes and clinical parameters [80-82]. For miRNA profiling we chose a bead-based flow-cytometric miRNA expression platform, which has recently been developed and was found to have several advantages over glass-slide microarray profiling, including increased specificity [56]. We developed this platform further to include 333 probes for 309 unique human miRNAs based on the miRNA repository miRBase 8.1 [17,18]. miRNA labeling included RNA size selection using native polyacrylamide gels, ensuring that only mature miRNAs were assayed.

Using this miRNA expression platform we analyzed a total of 137 samples in 168 assays. Assays for 119 of these 137 samples (87%) passed our quality control, including 93 primary tumor samples, 5 normal breast samples and 21 cell lines (Additional data file 1). We detected the expression of 137 miRNAs in this sample set, 133 of which we detected in normal breast or breast tumors. We included a number of replicate probes and technical replicate samples and found that results were reproducible (Additional data files 5 and 6). For a subset of miRNAs and a subset of samples we also performed quantitative RT-PCR to independently assess miRNA expression (Additional data file 7). While there is generally good correlation between miRNA expression on both platforms, we do observe probe-specific differences. Sample quantity did not permit validation of miRNA expression using northern blotting; however, the bead-based flow-cytometric miRNA expression platform had been validated using northern blotting previously [56].

**Figure 1:**
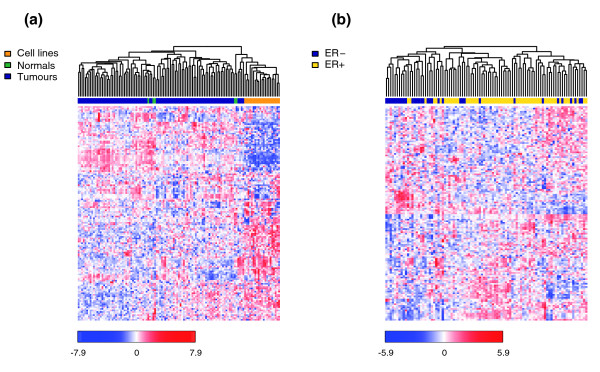
Unsupervised hierarchical clustering (Pearson correlation, average linkage) over 137 detected miRNAs. Heatmap colors represent relative miRNA expression as indicated in the color key. **(a) **Clustering of 21 cell lines (orange), 5 normal breast samples (green) and 93 primary tumors (blue). **(b) **Clustering of 93 primary tumors with ER status as shown.

Unsupervised hierarchical clustering of miRNA expression clearly separated cell lines from both normal breast and tumor samples and suggested that miRNA expression in cell lines is largely deregulated (Figure 1a). We did not observe a perfect separation of normal and tumor samples, as has been described before for primary human tumors [56]. However, as our study was focused on tumor subtypes, we profiled only a small number of normal breast samples. As we found major differences in miRNA expression between primary human tissue and cell lines, we excluded cell lines from subsequent analyses. Unsupervised clustering of the tumor samples revealed striking differences in miRNA expression between ER- and ER+ tumors (Figure 1b).

### MicroRNAs are differentially expressed between molecular breast tumor subtypes with clinical implications

**Figure 2:**
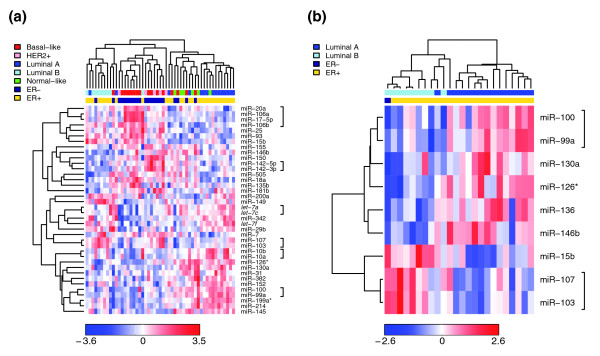
Supervised hierarchical clustering over selected miRNAs (Pearson correlation, average linkage). Heatmap colors represent relative miRNA expression as indicated in the color key for each panel. Brackets in the right margin indicate members of the same miRNA family. **(a) **Clustering of 51 tumor samples that could be classified as basal-like (red), HER2+ (pink), luminal A (dark blue), luminal B (light blue) or normal-like (green) over 38 miRNAs with Benjamini-Hochberg adjusted Kruskal-Wallis *p *< 0.05. **(b) **Clustering of 24 tumor samples classified as luminal A (dark blue) or luminal B (light blue) over 9 miRNAs with Benjamini-Hochberg adjusted Wilcoxon *p *< 0.05.

Next we tested if miRNAs are differentially expressed among breast cancer subtypes. To identify the molecular subtypes of our tumor samples we used a single sample predictor (SSP), which classifies breast tumors into five subtypes: luminal A, luminal B, basal-like, HER2+ and normal-like [65,69,70,76]. In addition to differences in mRNA expression profiles, these tumor subtypes also display distinct clinicopathological characteristics, including different survival rates (Additional data files 8 and 9). For example, the basal-like and HER2+ tumors are less differentiated and more aggressive and the luminal A and luminal B tumors are mostly ER+ with good and poor clinical outcome, respectively. Based on Agilent and Illumina mRNA expression data for 86 of our tumor samples [83] (unpublished results) we were able to classify 51 of the 93 tumor samples as 16 basal-like, 15 luminal A, 9 luminal B, 5 HER2+ and 6 normal-like tumors (Additional data file 1). miRNAs that were found to be differentially expressed in the tumor subtypes are shown in Figure 2a,b. miRNAs that are part of the same family show highly correlated expression. For example, the nine miRNAs that were found to be differentially expressed between luminal A and luminal B tumors represent seven miRNA families (Figure 2b).

Given the large number of miRNAs differentially expressed between molecular subtypes, we investigated the predictive potential of miRNAs in an independent test set. Using all 137 expressed miRNAs, we performed a model-based discriminant analysis [84] for the 16 basal-like and 15 luminal A tumors, the two largest subtype groups in our study (Additional data file 1). As we aimed to distinguish between molecular subtypes, we required a test set of samples with both miRNA and mRNA expression data available. The bead-based miRNA expression data in Lu *et al*. [56] included 11 breast tumor samples with corresponding Affymetrix gene expression data published in [56,85]. To our knowledge, no other studies with miRNA and mRNA data on breast tumor samples have been published. Based on the gene expression profiles and the SSP, six tumor samples could be assigned to molecular subtypes, three of which were classified as basal-like, two as luminal A and one as HER2+ (Additional data files 1, 14 and 19). Using the discriminator derived from our miRNA data, all three basal-like and two luminal A tumors in the independent miRNA data set were classified in concordance with their SSP molecular subtype classification.

### A number of miRNAs are associated with clinicopathological factors

**Figure 3:**
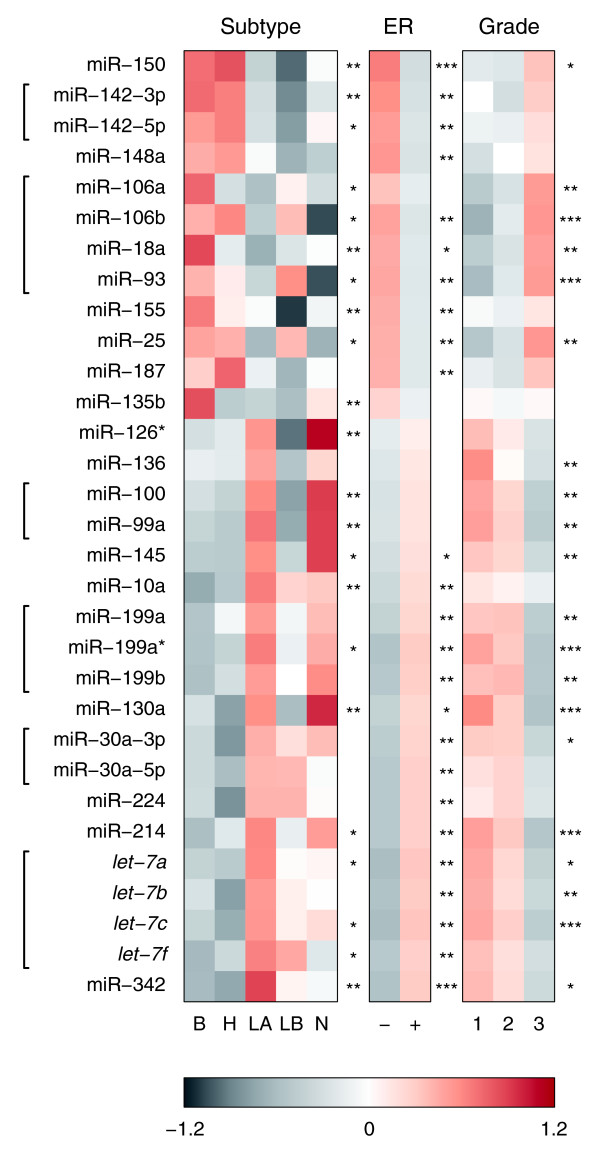
Association of individual miRNAs and tumor subtype or clinicopathological factors. Shown are 31 miRNAs and three factors with at least one association at adjusted *p *< 0.01. Differential expression was assessed by a non-parametric Wilcoxon rank sum test for comparison between two groups or a non-parametric Kruskal-Wallis test for comparison between multiple groups. To address the issue of multiple testing for the same factor, *p *values were adjusted by Benjamini and Hochberg's method [102]. Heatmap colors represent relative miRNA expression as shown in the color key. The expression values for a given sample group of interest were summarized by their mean. Brackets in the left margin indicate members of the same miRNA family. Significance levels are shown in the right margins: * adjusted *p *< 0.05; ** adjusted *p *< 0.01; *** adjusted *p *< 0.001. Abbreviations for subtype: B, basal-like; H, HER2+; LA, luminal A; LB, luminal B; N, normal-like.

We next assessed associations between individual miRNAs, molecular tumor subtypes and clinicopathological factors (Figure 3 and Additional data file 18). We tested for statistically significant associations with tumor characteristics such as molecular subtype, grade, stage, vascular invasion, ER status, Nottingham Prognostic Index (NPI) as well as TP53 status as determined by mutation screening and HER2 status assessed by immunohistochemistry (unpublished results). Figure 3 summarizes data for those 31 miRNAs and clinical factors for which there are significant associations at an adjusted *p *value less than 0.01 (Materials and methods). These 31 miRNAs represent 20 distinct miRNA families. Most of these miRNAs are expressed in the less aggressive, grade 1, ER+ samples. However, some miRNAs are expressed in the more aggressive grade 3/ER- tumors. We did not find any strong associations with stage, vascular invasion, NPI, TP53 or HER2 status.

### Chromosomal loss or gain cannot explain the majority of changes in miRNA expression

Given the changes in miRNA expression we observed, it is important to ask how these changes come about. We first tested if the changes in miRNA expression are likely due to chromosomal loss, gain or amplification as inferred from array comparative genomic hybridization (CGH) data. For 82 of the 93 tumor samples we analyzed for miRNA expression, we performed array CGH analysis based on gene centric oligonucleotide microarrays [86,87]. For each miRNA locus that was identified as altered in any of the samples, we performed separate non-parametric Wilcoxon rank sum tests to assess differences in expression between samples with loss, gain or amplification compared to samples without changes.

**Figure 4:**
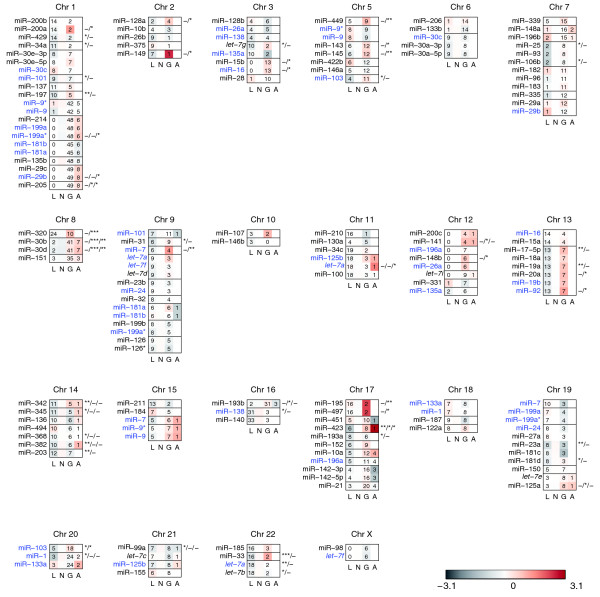
Association of miRNA expression and DNA copy number. miRNAs mapping to regions of genomic aberration were plotted according to chromosome and genomic location. Heatmap colors represent relative miRNA expression as shown in the color key. Expression values for samples with genomic loss (L), unaltered samples (N), samples with genomic gain (G) and amplification (A) were summarized by their mean, respectively, with numbers of samples as indicated. miRNAs transcribed from multiple loci are indicated in blue. Adjacent miRNAs not separated by a black line are less than 50 kb apart. Significance levels correspond to unadjusted *p *values obtained by a non-parametric Wilcoxon rank sum test (* *p *< 0.05, ** *p *< 0.01, *** *p *< 0.001). Given the high dependence of the performed tests, *p *values were not adjusted for multiple testing.

We found that in many cases expression differences could not be explained by any genome alterations detected by our array CGH data (Figure 4). The expression of 17 out of 129 mature miRNAs transcribed from genomic regions with an observed aberration correlated with genomic changes at 15 distinct chromosomal loci (*p *< 0.01). For miR-33 and miR-320, we found strong associations between miRNA expression and genomic alterations (*p *< 0.001), suggesting chromosomal change is a possible mechanism for mis-expression of these genes in primary human breast cancers. We also identified miRNA clusters whose changes in expression were correlated with copy number, for example, for miR-30b and miR-30d at C8q24.22 (*p *< 0.001) and miR-15b and miR-16-2 at C3q26.1 (*p *< 0.05).

### Expression of clustered miRNAs is coordinated

**Figure 5:**
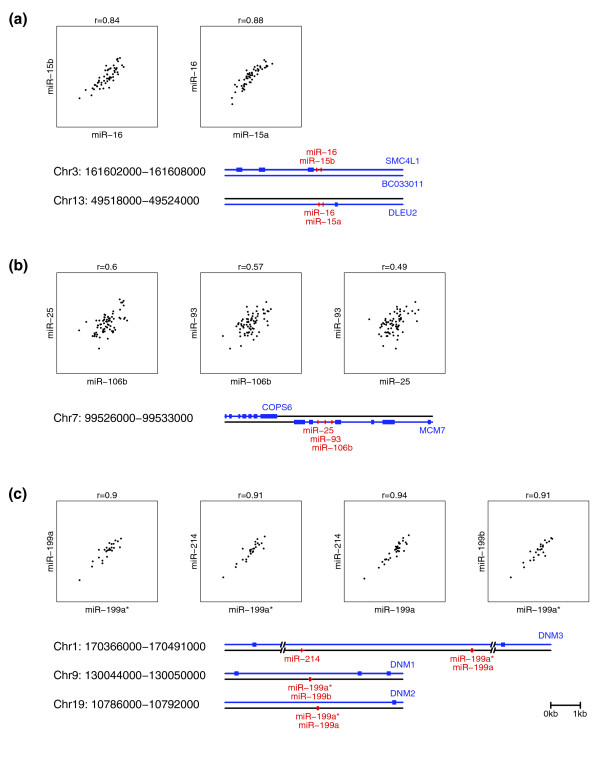
Expression of clustered miRNAs is coordinated. Shown are pairwise scatter plots of expression values for mature miRNAs transcribed from genomic regions within 50 kb of each other. **(a) **miR-15a, miR-15b and miR-16 transcribed from two intronic clusters at C3q26.1 (*SMC4L1*) and C13q14.3 (*DLEU2*). **(b) **miR-25, miR-93 and miR-106b transcribed from an intronic cluster at C7q22.1 (*MCM7*). **(c) **miR-199a, miR-199a*, miR-199b and miR-214 transcribed from one intergenic cluster at C1q24.3 and two intergenic stem-loops at C9q34.11 and C19p13.2. Pearson correlation coefficients (r) and data points shown are based on samples with available array CGH data and no identified genomic loss or gain at the relevant locus (Additional data file 1). Genome plots are drawn to scale as shown in the legend (bottom right), except where missing regions are indicated by vertical bars. Positive and negative strands are depicted by the top and bottom plots, respectively. Gene loci and miRNA stem-loop regions are colored in blue and red, respectively. The location of exons is marked by greater line width.

We noticed that miRNA clusters are often expressed coordinately in our sample set. For example, miR-106b, miR-93 and miR-25 situated on C7q22.1 are highly expressed in high-grade tumors. To further examine this phenomenon, we calculated the pairwise Pearson correlation of expression between miRNAs on the same chromosome and strand. We observed an abrupt drop in correlation of miRNA expression for pairs of miRNAs that were more than 50 kb apart (Additional data file 12). These observations agree well with what has been observed earlier in human tissue samples [88]. We therefore used a distance of 50 kb as a cut-off to identify 56 intergenic or gene-resident spatial clusters, 44 of which are represented in the set of 137 miRNAs detected in our sample set. Interestingly, 26 of 31 clusters for which expression data from multiple stem-loop regions were available show correlated expression with r > 0.4 (Figure 5 and Additional data file 13). For example, the miR-15 and miR-16 family are expressed from two clusters at chromosomes 3q and 13q, which are both highly correlated (r > 0.8). In many cases these correlations are likely due to shared regulatory elements or polycistronic expression of several miRNAs from a single primary transcript [88].

### A number of miRNA genes are co-regulated as part of larger domains

Since only 17 of the 137 miRNAs expressed in our samples showed changes in their expression associated with detected chromosomal abnormalities, changes in miRNA expression may be due to changes in transcription of primary miRNA transcripts. We showed above that miRNA clusters are expressed coordinately. We therefore asked if expression levels of miRNAs that are intronic are correlated with the expression of their host gene, as this suggests changes in primary transcription rates. To test this hypothesis, we compared miRNA expression data with Illumina mRNA expression data for our tumor sample set (unpublished results; Additional data file 13). We only detected correlations for seven miRNA host gene pairs (r > 0.4), suggesting that changes in miRNA expression in our tumor sample set are not generally linked to host gene expression (Table 1). These seven miRNA host gene pairs were miR-30e-5p/*NFYC*, miR-149/*GPC1*, miR-25/93/106b/*MCM7*, miR-342/*EVL *and miR-99a/*C21orf34*.

**Table 1:** MicroRNA/proximal probe correlations

		Host gene		Proximal probe		
			
miRNA	Chromosome position	Name	Pearson correlation	Name^†^	Pearson correlation	miRNA/probe distance (kb)
miR-101	1p31.1			*FLJ26232*	0.4337	35.99
miR-30e-5p	1p34.2	*NFYC*	0.4950			17.02
miR-181a	1q31.3			Hs.497310	0.4106	19.61
miR-205	1q32.2			*NPC-A-5*	0.7936	0.00
miR-10b	2q31.1			*HOXD10*	0.4902	30.96
				*HOXD8*	0.4472	18.53
miR-149	2q37.3	*GPC1*	0.6567			11.68
miR-30a-3p	6q13			*BC040204*	0.6406	16.17
miR-30a-5p	6q13			*BC040204*	0.7091	16.17
				*C6orf155*	0.4995	11.12
miR-30c	6q13			*BC040204*	0.4566	10.30
miR-106b	7q22.1	*MCM7*	0.5157			1.02
miR-25	7q22.1	*MCM7*	0.4939			0.59
miR-93	7q22.1	*MCM7*	0.4988			0.79
miR-181a	9q33.3	*NR6A1*^‡^		*R08260*	0.5913	1.51
miR-181b	9q33.3	*NR6A1*^‡^		*R08260*	0.5634	2.78
miR-196a	12q13.13			*HOXC10*	0.4914	1.75
miR-342	14q32.2	*EVL*	0.7208			34.26
miR-10a	17q21.32			GI_30159691	0.8123	0.40
				*HOXB6*	0.8085	16.10
				*HOXB5*	0.7150	11.48
				*HOXB3*	0.6908	29.79
				*HOXB2*	0.7019	36.74
				*HOXB4*	0.6856	2.90
				*HOXB8*	0.5937	32.43
miR-199a*	19p13.2	*DNM2*^‡^		*TMED1*	0.4111	15.22
miR-99a	21q21.1	*C21orf34*	0.4766			67.87
miR-155	21q21.3			*BIC*	0.4688	0.73
*let-7a*	22q13.31			*FLJ27365*	0.5272	1.45
*let-7b*	22q13.31			*FLJ27365*	0.5732	2.38

^†^Gene symbol, accession number or Illumina probe identifier. ^‡^Gene lies on the opposite strand.

For miRNA genes that are intergenic, we performed a similar comparison using the most proximal probes (within 50 kb) from the Illumina platform as these probes might correspond to primary miRNA transcripts (Additional data file 13). Only 23 out of 243 miRNA/proximal probe pairs at 11 distinct loci correlated in expression (r > 0.4; Table 1). Some of these miRNAs have proximal probes that are very close and likely represent primary miRNA transcripts. For example, miR-205 expression is highly correlated with the proximal probe for transcript *NPC-A-5 *(r > 0.75). One striking example of correlated expression of miRNAs and proximal probes was miR-10a, which is part of the *HOXB *cluster (C17q21.32), where Illumina probe data suggest the co-regulation of a region from *HOXB2 *to *HOXB6 *including miR-10a (Table 1).

### Some changes in miRNA expression may be due to changes in miRNA biosynthesis

**Figure 6:**
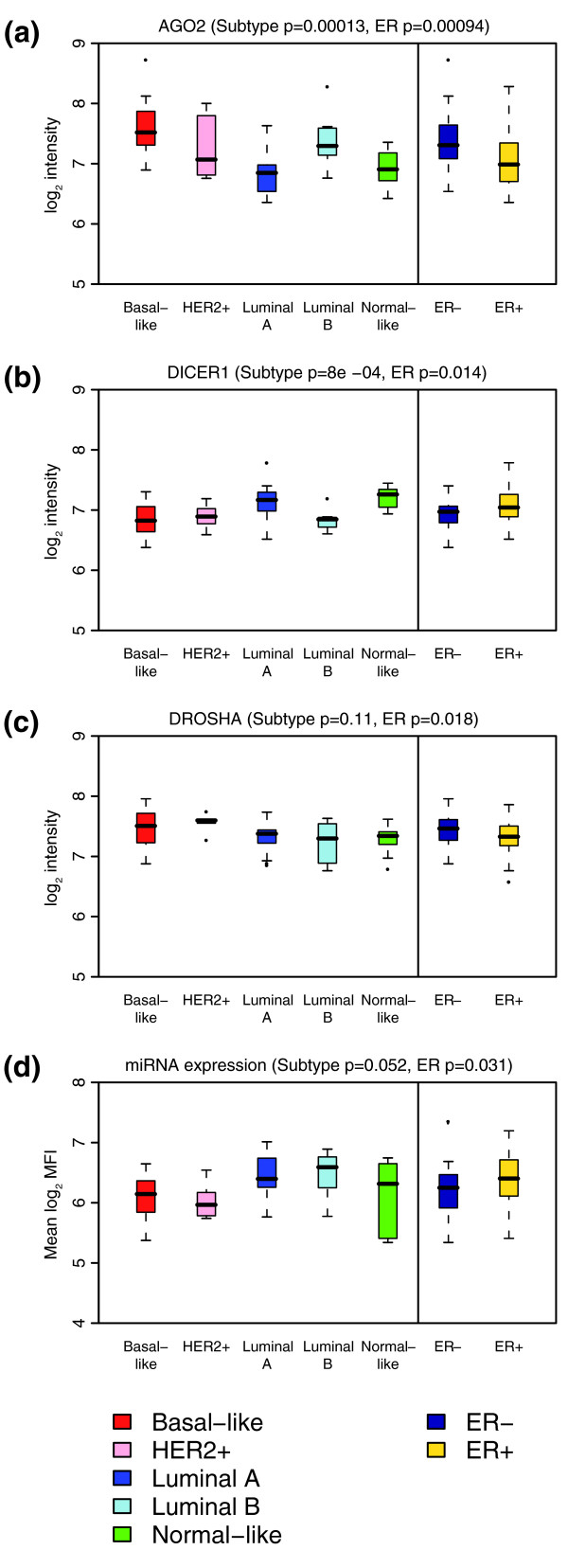
Genes required for miRNA biogenesis are differentially expressed according to molecular subtype and ER status. Shown are boxplots of Illumina log_2 _intensities for **(a) ***AGO2 *(*EIF2C2*), **(b) ***DICER1*, **(c) ***DROSHA *(*RNASEN*). Data are based on 58 samples that could be classified according to molecular subtype (17 basal-like (red), 5 HER2+ (pink), 18 luminal A (dark blue), 8 luminal B (light blue), 10 normal-like (green)) and 99 samples with known ER status (31 ER- (blue), 68 ER+ (yellow)). **(d) **Boxplots of mean miRNA expression after control-based normalization. Data are based on 51 samples that could be classified according to molecular subtype (16 basal-like (red), 5 HER2+ (pink), 15 luminal A (dark blue), 9 luminal B (light blue), 6 normal-like (green)) and 93 samples with known ER status (33 ER- (blue), 60 ER+ (yellow)). Black bars indicate the median; boxes interquartile range; whiskers most extreme data points not exceeding 1.5 times the interquartile range; points outliers. *P *values are based on non-parametric Kruskal-Wallis tests for subtype and Wilcoxon rank sum tests for ER status.

As genomic changes and transcriptional regulation of miRNA expression do not explain the changes in miRNA expression we observed in human breast cancers, post-transcriptional regulation of miRNA expression has to be considered. Indeed, recent studies suggested that primary miRNA processing might be deregulated in human cancer [64,89,90]. Therefore, we tested whether genes required for miRNA biogenesis are differentially expressed in our breast cancer samples. As we found many changes in miRNA expression across the five clinical tumor subtypes we had defined above (Figure 2), we asked whether *DICER1*, *DROSHA*, *DGCR8*, *AGO1*, *AGO2*, *AGO3 *or *AGO4 *expression differs among these subgroups. We found significant changes in the expression of *DICER1 *(*p *< 0.001), which was low in the more aggressive basal-like, HER2+ and luminal B type tumors, and *AGO2*, which was high in basal-like, HER2+ and luminal B type tumors (Figure 6). We did not find significant changes in the expression of *DROSHA*, *DGCR8*, or any of the other *AGO *genes (Figure 6 and Additional data file 10). We also observed significant changes in *AGO2*, *DICER1 *and *DROSHA *expression in relation to ER status, with *AGO2 *and *DROSHA *being higher and *DICER1 *lower in ER- tumor samples (Figure 6).

The observed deregulation of genes required for miRNA biogenesis may be expected to lead to global changes in miRNA expression. To further investigate this possibility, we utilized an alternative approach to between-sample normalization. For the analyses described previously, sample median centering proved advantageous in removing technical variation between samples without changing trends in differential expression (Additional data files 1 and 4). However, this method necessarily removed any global changes in miRNA expression. Using an alternative normalization based on spike-in controls, similar to the method described in [56], we detected small differences in mean miRNA levels according to ER status with lower mean miRNA expression in ER- tumors (Figure 6d).

## Discussion

Using an innovative bead-based miRNA expression profiling method we have determined the expression profile for 309 miRNAs in primary human breast cancer. We found that miRNA expression classified molecular tumor subtypes. Furthermore, a number of individual miRNAs were associated with clinicopathological factors. Changes in miRNA expression were complex and were likely due to genomic loss or gain, transcriptional and post-transcriptional regulation and changes in the expression of miRNA biogenesis enzymes. This study forms the basis for developing miRNA expression signatures as diagnostic tools for breast cancer and also furthers our understanding of the role of miRNAs in tumorigenesis.

Two previous studies of miRNA expression in human breast cancer have focused on comparing normal tissues to tumor samples. Here we focused on miRNA expression analysis of a large set of primary human tumors to reveal signatures of tumor subtype. Nevertheless, we also identified 7 out of 24 miRNAs that had previously been associated with breast cancers compared to normal tissues [78] (Additional data file 18). In addition, we can confirm three of 26 miRNAs that were reported in a separate study [77]. Notably, one miRNA, miR-155, is differentially expressed in ER- versus ER+ tumors (Figure 3), overexpressed in breast tumors compared to normal controls [77,78] and additionally other tumor types, suggesting that this miRNA may have diagnostic potential beyond breast cancer [54,91-93]. More recently, a quantitative RT-PCR study of miRNA expression from breast cancer biopsies revealed that miRNA expression classifies ER status [79], which is in agreement with our observations (Figure 1b). Surprisingly, we found little agreement among miRNAs we identified as being associated with clinicopathological factors and miRNAs identified in this context in a previous study [77].

We showed that a large number of miRNAs in our data set are associated with molecular subtypes, and we explored the predictive potential of miRNAs in an independent test set. A model-based discriminant analysis of our training set of 31 basal-like and luminal A samples resulted in the classification of 5 samples from an independent study that was in accordance with gene expression-based molecular subtype classification. Although these results are promising, the test set is too small to allow for a sensible performance assessment of the classifier. However, there are currently no other breast tumor data sets with both mRNA and miRNA expression data publicly available that would allow further validation of miRNA-based molecular subtype classification.

If miRNA expression profiles classify primary breast tumor subtypes, they may prove useful as diagnostic tools in the future and this could be assessed in a prospective study. Bead-based array miRNA profiling may be particularly well suited to assay miRNA expression in large-scale diagnostic trials since it is a high-throughput and cost-effective method [56,94]. If miRNAs prove useful for clinical breast cancer diagnosis, they have the additional advantage that, in contrast to most mRNAs, they are long-lived *in vivo *[35] and very stable *in vitro *[95], which might be critical in a clinical setting and allow analysis of paraffin-embedded samples.

We found that the differences in miRNA expression we observed are likely not due to genomic loss or gain (Figure 4). Therefore, we investigated the regulation of miRNA expression at the transcriptional and post-transcriptional level (Figure 5, Table 1). As previously described for normal human tissues [88], we found that the majority of miRNA clusters are co-regulated in human breast tumors. These data are also in agreement with similar observations made in human leukemia samples [96] and support the hypothesis that changes in miRNA expression in human cancer may not be distinct from normal tissue-specific miRNA expression in humans. In some instances, miRNA expression also correlates with host gene expression in the case of intronic miRNAs, or with the expression of larger domains, such as the *HOXB *cluster (Table 1 and Additional data file 13). In these instances, miRNA expression appears to be mainly under transcriptional control.

However, in many cases we observe that miRNA expression is not correlated with host genes or primary miRNA transcripts, suggesting post-transcriptional regulation of miRNA expression. Regulation of miRNA expression at the level of *DROSHA *has previously been proposed for human cancer [64,90]. We found that *DICER1 *expression is significantly downregulated in the more aggressive basal-like, HER2+ and luminal B type tumors. Interestingly, a recent study showed that downregulating *DICER1 *expression promotes tumorgenesis *in vitro *and in a mouse lung cancer model [97]. Together, these data suggest that *DICER1 *deregulation might be involved in the etiology of human breast cancer. In addition, we find that the deregulation of genes in the miRNA biogenesis pathway that we observed is in agreement with a number of independent data sets [98] (Additional data file 11).

Although both mRNA and miRNA expression profiles were found to be informative with regard to tumor subtype, their functional relationship remains unclear. In particular, we were interested to discover if changes in miRNA expression may correlate with changes in mRNA levels of direct targets (Additional data file 1). We considered miRNA families with identical seed (nucleotides 2-7) and mRNAs with conserved seed complementarity in their 3'UTR (Targetscan 3.1) [38]. A number of miRNA families showed differential expression between subtypes for their mean expression profile. We could detect only a few instances of enrichment for down- or up-regulation of predicted target mRNAs consistent with changes in miRNA expression, although previous studies of normal human tissue did observe such an enrichment [45,99]. However, these data are consistent with the hypothesis that many miRNAs act at the level of translation rather than mRNA stability.

## Conclusion

To date, many studies of miRNA expression in human cancer have focused only on the deregulation of miRNA expression. Here we integrated the analysis of miRNA expression, mRNA expression and DNA copy number in human breast cancer. Based on a combined analysis of miRNA and mRNA expression data we have identified a number of miRNAs that are differentially expressed between molecular tumor subtypes. In addition, we identified candidate miRNAs that are regulated at the genomic, transcriptional and likely post-transcriptional levels in breast cancer using miRNA, mRNA and array CGH data. Using mRNA expression data, we also found that the expression of genes in the miRNA biogenesis pathway is deregulated in breast cancer. We suggest that further analysis of integrated data sets might help to unravel miRNA-dependent pathways in human breast cancer.

## Materials and methods

### Sample collection

Primary breast tumor specimens were obtained with appropriate ethical approval from the Nottingham Tenovus Primary Breast Cancer Series (Nottingham City Hospital Tumor Bank, Nottingham, UK). All cases were primary operable invasive breast carcinomas collected from 1990 to 1996. Clinical information, including therapy, has been published previously [80-83,87].

### RNA extraction and labeling

RNA was extracted from primary tumors and cell lines using a standard Trizol (Invitrogen, Carlsbad, CA, USA) protocol, modified by washing the final RNA pellet with 80% EtOH. Frozen tumors were sectioned on a cryostat prior to homogenization in Trizol. RNA quantity and quality were assessed by Nanodrop (Nanodrop Technologies, Wilmington, DE, USA) and Agilent 2100 bioanalyzer (Agilent Technologies, Santa Clara, CA, USA), respectively.

miRNAs were extracted from 5 μg of sample total RNA using denaturing PAGE. Briefly, samples were spiked with three synthetic pre-labeling control RNAs (5'**-**pCAGUCAGUCAGUCAGUCAGUCAG-3', 5'-pGACCUCCAUGUAAACGUACAA-3', 5'-pUUGCAGAUAACUGGUACAAG-3'; Dharmacon, Lafayette, CO, USA) to control for target preparation efficiency, at 3 fmoles/sample. After purification of 18-26 bp RNAs, adaptors were ligated at the 3' and 5' ends using T4 RNA ligase (Fermentas, Burlington, OT, CA), a RNA-DNA hybrid 5'-pUUUaaccgcgaattccagt-idT-3' (Dharmacon; X = RNA, x = DNA, p = phosphate, idT = inverted [3'-3' bond] deoxythymidine) was ligated to the 3' end and 5'-acggaattcctcactAAA-3' (Dharmacon) was ligated to the 5' end using T4 RNA ligase. These bi-ligated products underwent reverse transcription using an adaptor specific primer (M37, 5'-pTACTGGAATTCGCGGTTA-3') and then amplified and labeled using PCR (M37 and M33, 5'-biotin-CAACGGAATTCCTCACTAAA-3'). Amplification was performed on an Eppendorf thermal cycler at 95°C for 30 s, 50°C for 30 s and 72°C for 40 s for 18 cycles. PCR products were precipitated without glycogen and redissolved in 66 μl 1 × TE buffer containing 1 μl of three biotinylated post labeling controls (100 fmols each, FVR506, PTG20210, MRC677).

### Bead coupling and hybridization

Oligonucleotide probes were coupled to color-coded polystyrene beads, allowing the simultaneous detection of about 90 different target oligonucleotides. To obtain expression profiles for 309 miRNAs, we created four distinct sets of bead-coupled miRNA probes. Each sample was hybridized to the four bead sets to generate a complete miRNA profile. Oligos were 5'-amino modified with a 6-carbon linker and conjugated to carboxylated xMAP beads (Luminex, Austin, TX, USA) in 96-well formats following the standard manufacturer's protocol. To generate bead set pools, 3 μl of each oligo-bead conjugate was mixed into 1 ml 1.5× TMAC buffer (4.5 M tetramethylammonium chloride, 0.15% sarkosyl, 75 mM Tris-HCl pH 8.0, 6 mM EDTA). Samples were hybridized in a 96-well format with two water-only blanks and at least three bead blanks containing water instead of the labeled sample for use as a background control. We included replicate probes and technical replicate samples across bead sets and sample plates, respectively, to aid quality control and data preprocessing. Hybridization was carried out overnight at 50°C with 33 μl of the bead pool and 15 μl of labeled sample.

Unbound sample was removed from beads by washing with 1 × TE and re-suspending in 1× TMAC buffer. SAPE, streptavidin-phycoerythrin, premium grade (Invitrogen) was added to the beads (1:100 dilution) and incubated for 10 minutes at 50°C to bind to biotin moieties on the cDNA. Samples were processed on a Luminex 100 machine and median fluorescence intensity values acquired using the StarStation software (ACS, Sheffield, UK).

### Computational analysis

#### Preprocessing

Median fluorescence intensity values smaller than a threshold of 1 were set equal to 1, and all values were transformed by taking logs (base 2). Samples with low mean expression were excluded from further analyses (Additional data files 1 and 3). To reduce noise due to absent probes, each probe was required to exceed a log_2 _median fluorescence intensity value of 6 in at least one sample. Systematic probe effects were median-corrected (Additional data files 1 and 2). Replicate probes were summarized by their mean profile and samples were centered to have zero median. Technical replicate samples were summarized by their mean profile. For a more detailed description of preprocessing please see Additional data file 1.

#### Genomic annotation

miRNA probe sequences were matched against stem-loop sequences in miRBase (release 8.1). Genomic miRNA clusters were identified by requiring any two stem-loops on the same chromosome and strand within 50 kb to belong to the same clusters (Additional data file 16). A miRNA was defined as gene-resident if its stem-loop is completely contained in the locus of a gene transcript on the same chromosomal strand as annotated in the Known Genes and RefSeq Genes tables obtained from the UCSC Genome Browser (hg18) [100] (Additional data file 17).

#### Illumina gene expression data

Illumina gene expression data were processed and summarized in the Illumina BeadStudio software. Analyses of the probe level data were performed using the *beadarray *Bioconductor package [101]. After quality control, between-array qspline normalization was performed for 112 arrays for 99 samples. Replicate arrays were averaged and expression values were transformed by taking logs (base 2).

### Subtype classification

Each array in the preprocessed Illumina and Agilent [83] gene expression data set was normalized to have zero mean and standard deviation one, and each probe was centered to have zero median. An SSP annotation for Agilent probes was provided in [76]. Detailed information on the SSP annotation for Illumina probes can be found in Additional data files 1 and 15. Multiple probes for the same UniGene cluster ID in either data set were summarized by their median profile. Samples were then assigned to the nearest subtype centroid as determined by Spearman correlation, requiring a minimum correlation of 0.3. Samples that could be assigned to subtypes based on both Agilent and Illumina expression profiles were classified according to the Illumina assignment (Additional data file 1).

#### Hierarchical clustering

Prior to hierarchical clustering, miRNA profiles were standardized to have mean zero and standard deviation one. Clustering was performed with average linkage and Pearson correlation.

#### Supervised analyses

Differential expression was assessed by a non-parametric Wilcoxon rank sum test for comparison between two groups or a non-parametric Kruskal-Wallis test for comparison between multiple groups. To address the issue of multiple testing for the same contrast, adjusted *p *values were obtained by Benjamini and Hochberg's method [102].

Copy-number-driven expression

For each miRNA stem-loop identified as gained, lost or amplified in any of the samples, separate non-parametric Wilcoxon rank sum tests were performed to assess differences in expression between samples with genomic changes and unaltered samples [103]. *P *values were not adjusted for multiple testing due to the high level of dependence between the performed tests.

Coexpression of proximal miRNAs and Illumina probes

For a given chromosome and strand, pairwise Pearson correlation coefficients were calculated for all miRNA probes and those Illumina probes mapping to a host gene or within 50 kb of a miRNA stem-loop. To account for coexpression caused by DNA copy number changes, correlation coefficients for probe pairs were calculated using only those samples with available array CGH data showing no evidence for aberration at either locus (Additional data file 1).

All analyses were performed in the statistical programming environment R [104] using customized functions and functions available from Bioconductor [105,106] and the MCLUST package [107]. All miRNA expression data have been submitted to the Gene Expression Omnibus (GEO) with accession number GSE7842.

## Abbreviations

CGH, comparative genomic hybridization; ER, estrogen receptor; miRNA, microRNA; NPI, Nottingham Prognostic Index; SSP, single sample predictor.

## Authors' contributions

CB, CC and EAM conceived and designed the study. ARG and IOE provided breast cancer samples and clinical information. CB, IS and SC performed the experiments under the supervision of CC and EAM. The statistical analysis and experimental design were conducted by LDG and supervised by NPT. ST and AET provided statistical advice. MD preprocessed the Illumina gene expression data. NLBM provided the Illumina probe annotation. CB, LDG, NPT, ST, CC and EAM wrote the manuscript.

## Additional data files

The following additional data are available with the online version of this paper. A detailed description of the computational analysis carried out is given in additional data file 1 and a layout of the experimental design is shown in additional data file 2. Additional data on miRNA expression analysis can be found in additional data file 3 (pre-processing), additional data file 4 (normalization), additional data file 5 (replicate probes), additional data file 6 (replicate samples) and additional data file 7 (qRT-PCR validation). Additional data file 8 contains a mRNA expression heatmap for 82 classified samples and 75 intrinsic genes, and additional data file 9 contains a pairwise comparison of Kaplan-Meier survival curves for 74 classified samples with available follow up data. Additional data on differential expression of miRNA processing genes can be found in additional data file 10 (this data set) and additional data file 11 (other data sets). Additional data file 12 shows the correlation of proximal miRNA probes and additional data file 13 shows correlations between miRNA probes and Illumina probes. Additional data file 14 shows a model-based discriminant analysis for Basal-like and Luminal A tumors. Additional data file 15 contains annotation for the intrinsic gene probe set (single sample predictor). Additional data file 16 lists spatial miRNA clusters, additional data file 17 lists host gene coordinates of intragenic miRNAs and additional data file 18 associations between individual miRNAs, molecular tumor subtypes and clinicopathological factors. Additional data file 19 contains the intrinsic gene probe sets used for the model-based discriminant analysis.

## Supplementary Material

Additional data file 1 This file contains a detailed description of the computational analyis. Click here for file

Additional data file 2 A. Data matrix of miRNA expression values (schematic). The 333 rows and 168 columns correspond to probes and samples respectively. Expression values for each sample were obtained from hybridizations to four distinct bead sets (with approximately 90 probes each), carried out in separate wells of 96-well plates. Hybridizations were performed on eight plates, using two plates for each bead set. The allocation of samples between the two plates for a given bead set was kept consistent for all four bead sets. Thus both probes and samples could be ordered according to the plate of origin, partitioning the data matrix into eight blocks corresponding to measurements from distinct plates. Expression values for a representative well on plate 1 for beadset 1 are indicated in grey. B. Heatmap of unnormalized log_2 _MFI values for all miRNA probes and all samples. Probes were median centred prior to plotting. C. Heatmap of differences between the probe median for the randomized samples on a given plate and the probe median for all samples on both plates. Click here for file

Additional data file 3 A. Histograms of log_2 _MFI values obtained from wells containing sample material (white) and blank control wells (blue). B. The number of detected probes after filtering was plotted against a range of cutoff values. Probes were removed (filtered) if they did not exceed the chosen cutoff (red) in at least one sample. C, D. Sample quality control. Pearson correlation coefficients for technical replicate samples were plotted against the smaller of the two sample means for (C) cell line technical replicate samples and (D) normal and tumor technical replicate samples. The cutoff used for quality control is indicated by a vertical line. Colours corresponding to sample status are explained in the colour key. E. Technical sample effects. Pairwise differences between the medians of technical replicate samples were plotted for unnormalized data (black), data normalized based on spike-in controls (blue) and data normalized by sample median centering (red). Dashed lines indicate the maximum difference between the medians of any two samples for unnormalized data (black) and for data normalized using spike-in controls (blue). Click here for file

Additional data file 4 Between-sample normalization. A. Shown are data normalized based on spike-in controls for the same miRNAs and factors as in Figure 3 in the main text. B. miRNAs and factors with at least one association at adjusted p < 0.01 based on data normalized using spike-in controls. All miRNAs thus identified were also identified after sample median centering with the exception of miR-152, which was found to be associated with all three factors at p < 0.05 (Additional data file 18). Heatmap colours reflect relative miRNA expression. The expression values for a given sample group of interest were summarized by their mean. Brackets in the left margin indicate members of the same miRNA family. Significance levels are indicated in the right margins: * adjusted p < 0.05, ** adjusted p < 0.01, *** adjusted p < 0.001. Abbreviations for subtype: B = Basal-like, H = HER2+, LA = Luminal A, LB = Luminal B, N = Normal-like. Click here for file

Additional data file 5 Pairwise scatter plots of replicate probes after sample quality control, probe filtering and within-plate probe correction (none of the replicated probes were removed due to probe filtering). Scatter plots for one failed probe (miR-224-4) are marked in red. Click here for file

Additional data file 6 Pairwise scatter plots of technical replicate samples after sample quality control, probe filtering, within-plate probe correction and summarizing replicate probes. Click here for file

Additional data file 7 Normalized log_2 _MFI values were plotted against log_2_-transformed and median-corrected measurements obtained by qRT-PCR Click here for file

Additional data file 8 Expression values are based on Illumina data when available, and Agilent data otherwise. The two data sets were normalized as described. Missing values in the Agilent data are indicated in white. Samples were ordered according to molecular subtype (see colour key). The heatmap does not present a hierarchical clustering but merely illustrates differences in gene expression. A. Luminal/ER+ gene cluster. B. ERBB2 and GRB7-containing cluster. C. Interferon-regulated cluster including STAT1. D. Basal epithelial cluster. E. Proliferation cluster. Click here for file

Additional data file 9 Pairwise comparison of Kaplan-Meier survival curves for 74 classified samples with available follow up data (21 Basal-like, 7 HER2+, 25 Luminal A, 10 Luminal B, 11 Normal-like). A non-parametric log rank test was used to assess differences in clinical outcome. Click here for file

Additional data file 10 Shown are boxplots of log_2 _expression for *DGCR8*, *DICER1*, *DROSHA *(*RNASEN*), *AGO1 *(*EIF2C1*), *AGO2 *(*EIF2C2*), *AGO3 *(*EIF2C3*) and *AGO4 *(*EIF2C4*). The data were obtained for 58 samples classified according to subtype (17 Basal-like, 5 HER2+, 18 Luminal A, 8 Luminal B, 10 Normal-like) and 99 samples with known ER status (31 ER-, 68 ER+). We only included Illumina probes not mapping to introns and which could be detected at log_2 _expression 6 in at least one sample. Differential expression was assessed using a non-parametric Kruskal-Wallis test (subtype) and Wilcoxon rank sum test (ER status). Click here for file

Additional data file 11 Shown are boxplots of normalized gene expression units for each candidate gene that showed differential expression (Student's t-test p < 0.001). Data were obtained from the cancer microarray database ONCOMINE [98], and differential expression was assessed using Student's t-test. Each row of plots corresponds to a unique gene; data obtained from different studies are separated by a solid vertical black line. For each data set the number of ER negative (blue) and ER positive (yellow) samples is included in the lower figure margin. The first authors of the relevant publications are included in the plot title. Click here for file

Additional data file 12 Pearson correlation coefficients for mature miRNAs mapping to the same chromosome and strand were plotted against decreasing ranks of pairwise distances. Diamonds represent a moving average over five correlation coefficients. The absolute distance is plotted in blue and indicated on the right y-axis. Distance 50 kb is indicated by a vertical red line. Click here for file

Additional data file 13 Heatmap of Pearson correlation coefficients (accounting for DNA copy number changes as described) between miRNA probes and selected Illumina probes on the same chromosome and strand. Blank entries are due to missing DNA copy number information. Probes are arranged in genomic order. Black boxes indicate clusters of adjacent probes less than 50 kb apart. Green boxes indicate clusters of probes mapping to the same host gene. Mature miRNAs included in multiple stem-loops are indicated in blue. Relative genomic probe positions are marked as white bars on the chromosomal plot below each heatmap. Click here for file

Additional data file 14 SSP molecular subtype classification based on the Affymetrix gene expression data for normal breast and breast tumor samples in [56,85]. Spearman correlations with the five subtype centroids are shown for all 14 samples. The solid horizontal black line indicates the minimum correlation required for subtype assignment. If the minimal correlation with a subtype centroid was achieved, the classification was made using the centroid with highest Spearman correlation. B. Shown are class posterior probabilities for 16 Basal-like and 15 Luminal A tumors in the training set (using all detected 138 miRNAs); and three Basal-like and two Luminal A tumors in the test set (using the 77 detected miRNAs in common with the training set). Red and blue indicate the posterior probability of belonging to the Basal-like and Luminal A subtype respectively. Plotting characters indicate the gene expression based subtype classification with squares and triangles representing Basal-like and Luminal A samples respectively. Samples were assigned to the class with posterior probability greater than 0.5 (solid horizontal black line). Click here for file

Additional data file 15 Intrinsic genes, probe sets (single sample predictor) Click here for file

Additional data file 16 Spatial miRNA clusters Click here for file

Additional data file 17 Host gene coordinates of intragenic miRNAs Click here for file

Additional data file 18 Associations between individual miRNAs, molecular tumor subtypes and clinicopathological factors Click here for file

Additional data file 19 Intrinsic genes, probe sets (model-based discriminant analysis) Click here for file
