# Supplementary material for: MicroRNA expression profiling of human breast cancer identifies new markers of tumor subtype
Source: Genome Biol. 2007 Oct 8;8(10):R214. doi: 10.1186/gb-2007-8-10-r214 (PMC2246288; doi:10.1186/gb-2007-8-10-r214)
Supplement: Additional data file 1 — This file contains a detailed description of the computational analyis. [file gb-2007-8-10-r214-S1.pdf]

SUPPLEMENTARY NOTES

MicroRNA expression profiling of human breast cancer identifies new markers of tumour subtype

Cherie Blenkiron[1,2,3,4,*], Leonard D Goldstein[1,2,5,*], Natalie P Thorne[1,2,5], Inmaculada Spiteri[1,2], Suet-Feung Chin[1,2], Mark J Dunning[1,2], Nuno L Barbosa-Morais[1,2], Andrew E Teschendorff[1,2], Andrew R Green[6], Ian O Ellis[6], Simon Tavaré[1,2,5], Carlos Caldas[1,2,$], Eric A Miska[3,4,$]

[*] These authors contributed equally to this work.

[1] Cancer Research UK, Cambridge Research Institute, Li Ka-Shing Centre, Robinson Way, Cambridge, CB2 0RE, UK

[2] Department of Oncology, University of Cambridge, Hills Road, Cambridge CB2 2XZ, UK

[3] Wellcome Trust/Cancer Research UK Gurdon Institute, University of Cambridge, The Henry Wellcome Building of Cancer and Developmental Biology, Tennis Court Rd, Cambridge, CB2 1QN, UK

[4] Department of Biochemistry, University of Cambridge, 80 Tennis Court Rd, Cambridge, CB2 1GA, UK

[5] Department of Applied Mathematics and Theoretical Physics, University of Cambridge, Centre for Mathematical Sciences, Wilberforce Road, Cambridge CB3 0WA, UK

[6] Department of Histopathology, School of Molecular Medical Sciences, University of Nottingham, Nottingham, NG5 1PB, UK

[$] Corresponding authors: email cc234@cam.ac.uk, phone +44-1223-331989, +44-1223-404420 (CC) or email eam29@cam.ac.uk, phone +44-1223-767220, +44-1223-767225 (EAM)

Experimental design

Bead-based flow-cytometric miRNA profiling is relatively novel, with only few publications utilizing the platform to date [56, 97]. We therefore designed the experiment in a manner that allowed us to investigate technical effects and assess the reproducibility of measurements for a given probe or target sample.

As described in [56] probes were coupled to colour-coded polystyrene beads allowing the simultaneous detection of about 90 different target oligonucleotides. To obtain expression profiles for 309 miRNAs we created four distinct sets of bead-coupled miRNA probes. To assess the reproducibility of measurements from distinct bead sets, twelve spare colour-coded beads were selected to carry the same probe in each bead set. These probes consisted of six replicated miRNA probes (*let-7a*, *let-7b*, miR-24, miR-26a, miR-181a, miR-224) and six control probes for synthetic oligonucleotides.

To assess the reproducibility of sample profiles, 16 out of the 137 samples were selected for replication. 15 samples were hybridized in triplicate and one sample in duplicate, resulting in a total of 168 samples.

The 168 x 4 hybridizations were carried out on eight 96-well microtiter plates, using two plates for each bead set (Additional data file 2; panel A). Samples were separated into two groups and, for a given bead set, hybridizations were allocated between plates accordingly. Each of the two groups included at least one replication for each of the 16 replicated samples.

Low-level analysis

Median fluorescence intensity (MFI) values smaller than a threshold of 1 were set equal to 1. The data were then transformed by taking logs (base 2) to achieve approximate independence of signal intensity and variance.

As described previously, we observed some signal for probes in blank control wells (median log_2 MFI 3.9) (Additional data file 3; panel A). To reduce the amount of noise due to probes for miRNAs which were absent in our samples, we performed filtering by requiring each probe to exceed a log_2 MFI of 6 in at least one sample. This value was based on the distribution of signal levels detected in the blank control wells and by considering the number of detected probes for a range of cutoff values (Additional data file 3; panel B).

Samples with low mean intensity were more strongly affected by noise. This was confirmed by considering pairwise correlations of the technical replicate samples over a range of intensities (Additional data file 3; panels C, D). Reduced reproducibility was observed for samples with mean smaller than approximately 5.5 on the log_2 scale. Sample quality control was performed by requiring the mean of each sample to exceed this cutoff. As miRNA expression was generally lower in cell lines, a large number of cell line samples were removed from further analyses. 142 out of 168 samples (119 out of 137 unique biological samples) passed quality control. After sample quality control probe filtering was repeated to exclude probes that exceeded the chosen

cutoff in the removed samples only. This second filtering step removed one additional probe. We thus detected signal for 161 out of 333 probes, corresponding to 137 unique miRNAs.

We observed previously unreported probe-specific plate effects (Additional data file 2; panels B, C). Hybridization efficiency for a given probe may differ between plates. Due to the non-uniform melting temperature of miRNA probes, small differences in hybridization temperature likely result in better hybridization conditions for some probes and less favourable conditions for others. The effect necessitates the correction for probe effects within plates and questions the use of spike-in controls for normalization purposes. We removed the effect by subtracting differences between the probe median for the randomized samples on a given plate and the probe median for all samples on both plates (with the same bead set).

Replicated probes were summarized by their mean expression profile. With the exception of one probe, profiles were highly correlated (median Pearson correlation excluding the failed probe 0.94, range from 0.80 to 1.00) (Additional data file 5). The failed probe was removed prior to summarizing replicate measurements. Between-sample normalization was performed by subtracting the median for each sample (see below). Expression profiles for technical replicate samples were then summarized by their mean profile. Replicated samples also showed highly correlated expression (median Pearson correlation 0.91, range from 0.80 to 0.97) (Additional data file 6).

Between-sample normalization

Between-sample normalization of gene expression data relies on the assumption that certain features of the sample distributions are equal across samples. In the context of miRNA expression data, assumptions commonly accepted for gene expression data may be too strong due to the small number of expression values or unjustified in the case of global changes in miRNA expression.

We compared sample median centering with a normalization based on spike-in controls similar to the method described in [56]. Briefly the $\log_2$-transformed control intensities were corrected for probe-specific plate effects as described above. Probe effects were removed by subtracting the probe median to obtain relative changes in spike-in control levels. Sample effects were then estimated by the arithmetic mean of eight measurements for two spike-in controls in each sample (one measurement per control and bead set). Samples were normalized by subtracting the estimated effect.

In our data we observed relatively large technical sample effects compared to overall differences in miRNA expression between samples. On the $\log_2$-scale differences between the medians of technical replicate samples were as high as 1 compared to 2.1 for the difference between the lowest and highest sample medians (Additional data file 3; panel E). Median centering samples removes both technical and biological global differences. The normalization based on spike-in controls is less likely to remove biological effects but spike-in controls were too variable to remove all technical variation (after control-based normalization differences between the

medians of technical replicate samples still attained 0.8 on the log_2-scale, see panel E in Additional data file 3).

Changes in miRNA expression between the groups of interest appeared to be sufficiently symmetric for sample median centering to remove technical variation without removing effects between groups. The normalization thus led to more significant differences between groups, without changing the trend of expression changes as compared to control-based normalization (Figure 3, Additional data file 4). All analyses in this study were based on data normalized by sample median centering, except for the analysis of global miRNA expression (Figure 6D), which relied on control-based normalization.

qRT-PCR validation

RNA from 12 tumour samples was used to validate 16 miRNAs detected by bead-based flow-cytometric profiling. TaqMan miRNA assays (Applied Biosystems, CA, USA) were used as per manufacturer protocol. For each sample 10 ng of total RNA was converted to cDNA using a miRNA specific probe. This product was then subjected to PCR on an ABI7900 HT and accumulation of product determined by fluorescence. PCR amplifications were performed in triplicate and quantified using a standard curve generated from running serial dilutions of an RNA (cDNA) normal/tumour pool. Measurements were transformed by taking logs (base 2) and median-corrected for run differences and RNA input (Additional data file 7).

Molecular subtype classification

Tumour samples with available gene expression data were classified into five molecular subtypes using a published single sample predictor (SSP) [76]. The SSP consists of five subtype centroids (mean expression profiles) for 306 intrinsic genes defined by UniGene Cluster IDs (Build 161). Individual samples are classified by assignment to the nearest subtype centroid as determined by Spearman correlation.

We used two gene expression data sets: data for 135 samples based on Agilent microarrays published in Naderi *et al.* [83] and unpublished data for 99 samples based on the Illumina platform. Together these data sets included gene expression data for 86 of the 93 tumour samples in the miRNA profiling study.

Annotation of the 306 intrinsic genes in terms of 326 Agilent probes is provided by [76], 323 of which were included in the Naderi data set. We could further annotate 324 Illumina probes for 296 of the intrinsic genes: For each intrinsic gene (UniGene Cluster ID) the representative accession number or gene symbol was mapped to GenBank accession numbers or gene symbols in the Illumina probe annotation respectively. Genes were mapped through gene symbols only if there was no match for the representative accession number. Illumina probes with multiple GenBank accession numbers or gene symbols were used only if no probe with unique GenBank accession number or gene symbol was available. A number of UniGene Cluster IDs could not be

mapped through representative accession number or gene symbol but could be annotated manually. The complete annotation is provided in Additional data file 15.

Consistent with the preprocessing of the combined test set for the SSP in [76], all samples were normalized to have zero mean and standard deviation one. Genes were median centred within each data set. Expression values for the previously annotated SSP probes were then extracted. Agilent samples with more than 50% missing SSP probe expression values were removed from further analyses. Multiple probes for the same intrinsic gene were summarized by the median expression profile.

Spearman correlation coefficients were calculated for each sample and subtype. To reduce the number of spurious classifications we required a minimum correlation of 0.3 with the nearest subtype centroid. For six out of 32 samples classified by Agilent and Illumina gene expression data, the classification differed between platforms. Although inconsistent classification might be due to technical bias, it also indicated that not all samples naturally fell into one of the five molecular subtype categories: For all six inconsistently classified samples the two nearest subtypes agreed between platforms, and in three cases correlation with the nearest subtype was only marginally higher than correlation with the second nearest subtype for both platforms.

To allow for a consistent analysis of the Illumina gene expression data (which was the primary gene expression data set used in this study), samples were classified according to the Illumina data whenever both classifications were available. In total 82 samples were classified (51 of which were included in the miRNA expression data), using either Agilent or Illumina data.

Molecular and prognostic significance of the subtype classification are illustrated in Additional data files 8, 9.

Model-based discriminant analysis for Basal-like and Luminal A tumours

We considered a training set consisting of all 16 Basal-like and 15 Luminal A samples and all 137 detected miRNAs. Prior to building the classifier all miRNAs were centred and scaled to have mean zero and standard deviation one. The R package MCLUST [84, 110-113] was used to produce a density estimate for the training data. The training data were assumed to be generated by a mixture distribution with density $f(x) = \tau_1 f_1(x) + \tau_2 f_2(x)$, where f_k is the probability density function of observations in class k, and τ_k is the probability that an observation comes from the kth mixture component ($0 < \tau_k < 1$ for $k = 1, 2$ and $\tau_1 + \tau_2 = 1$). The class conditional probability density functions were assumed to be multivariate normal $f_k(x) = \phi(x \mid \mu_k, \Sigma_k)$, with class mean μ_k and class covariance matrix Σ_k ($k = 1, 2$). Classification of new samples could then be achieved by considering the posterior class probabilities $\tau_k f_k(x) / (\tau_1 f_1(x) + \tau_2 f_2(x))$. Four parameterizations of the two covariance matrices were considered imposing the following cross-cluster constraints: (i) spherical clusters of the same size, (ii) spherical clusters with variable size, (iii) diagonal clusters of the same size, (iv) diagonal clusters with variable size. Leave-one-out cross validation resulted in an error rate of 0.097 for parameterizations (i) and (ii), and the most parsimonious model (i) was chosen to estimate the class densities.

As the desired classifier distinguishes between molecular subtypes, we required a test set of samples with both available miRNA and mRNA expression data. The miRNA expression data in [56] included three normal breast and 11 breast tumour samples. Affymetrix gene expression data for the 11 tumours were published in [85] and [56].

As suggested in [56], the preprocessed gene expression data for the 14 samples of interest were filtered by requiring each probe set summary value to exceed 7.25 on the $\log_2$ scale in at least one sample. Samples were normalized to have mean zero and standard deviation one. Probe set summary values were then median centred. 297 of the 306 SSP genes could be mapped to 423 probe sets included on the Affymetrix Human 6800 and Human 35KsubA chips (Additional data file 19). Multiple probe sets for the same intrinsic gene were summarized by the median probe set summary profile. Samples were assigned to the nearest subtype centroid as determined by Spearman correlation. The subtype centroid correlations for Affymetrix data were low compared to the Agilent and Illumina data. This may be due in part to median centering probe set summary values for only 14 samples, which is not robust and likely to remove biological information if the sample set is biased towards certain subtypes. A minimal correlation of 0.15 was required for subtype assignment. Reassuringly, all three normal breast samples were classified as Normal-like. Furthermore all four samples assigned to the aggressive Basal-like and HER2+ subtypes belonged to the poorly differentiated tumours from [56].

The preprocessed MFI values were extracted for the three normal breast and eleven breast tumour samples. Probes were filtered by requiring each probe to exceed a value of 7.25 on the $\log_2$ scale in at least one sample. After filtering, 77 miRNA probes were in common between

training and test set. Samples were median centred and probes were centred and scaled to have mean zero and standard deviation one.

The class conditional density of samples with a reduced number of variables $z = (x_i)$ for i taking values in a subset I of $\{1,\ldots,137\}$ is given by $f_k^*(z) = \phi(z \mid \mu^*_k, \Sigma^*_k)$ where $\mu^*_k = (\mu_{ki})$ and $\Sigma^*_k = (\Sigma_{kij})$ with i, j taking values in I. Hence classification based on the reduced set of variables could be achieved by considering the posterior class probabilities $\tau_k f_k^*(z) / (\tau_1 f_1^*(z) + \tau_2 f_2^*(z))$ (Additional data file 14).

miRNA processing genes

Having established the molecular subtype for 58 of the 99 samples included in the Illumina data set, we asked whether genes in the miRNA biogenesis pathway were differentially expressed between subtypes. We considered *DGCR8*, *DICER1*, *DROSHA* (*RNASEN*), *AGO1* (*EIF2C1*), *AGO2* (*EIF2C2*), *AGO3* (*EIF2C3*) and *AGO4* (*EIF2C4*). We only included data for Illumina probes which did not map to introns and could be detected at $\log_2$ intensity higher than 6 in at least one sample. Differential expression according to subtype was assessed using a non-parametric Kruskal-Wallis test, differential expression between ER status was assessed using a non-parametric Wilcoxon rank sum test (Additional data file 10).

The observed differential expression of miRNA processing genes according to ER status is in agreement with independent datasets included in the cancer microarray database ONCOMINE

[98]. Consistent with our data *AGO2*, *DICER1* and *DROSHA* were found to be differentially expressed (Student's t-test $p < 0.001$) in eight (out of 18) [67, 114-120], four (out of 13) [67, 120, 121] and one (out of 16) [67] studies respectively (Additional data file 11). For each of these genes, all data sets with $p > 0.001$ but $p < 0.05$ showed deregulation consistent with our results.

AGO1 exhibited significantly higher expression in ER negative tumours (Student's t-test $p < 0.001$) in two studies [67, 120], while *AGO3*, *AGO4* and *DGCR8* showed no differential expression at this significance level.

Finally we note that *AGO2* is one of the 306 intrinsic genes forming the single sample predictor in [76].

Genomic clustering of miRNAs

To examine the coordinate expression of clustered miRNAs we calculated the pairwise correlation of expression between miRNAs on the same chromosome and strand. Pearson correlation coefficients were plotted against the ranks of pairwise distances as determined by the genomic coordinates of the mature miRNAs (Additional data file 12). Correlations markedly dropped for miRNA pairs with distance greater than 50 kb. Consequently, any two miRNA stem-loop regions on the same chromosome and strand within 50 kb of each other were defined to belong to the same cluster. We thus defined 56 spatial clusters (Additional data file 16), 38 and

15 of which are intergenic and gene-resident respectively; three clusters partially overlap with gene loci. (Only 44 of the 56 clusters are represented by the probes included in our study.) For subsequent analyses, we further extracted gene- resident miRNAs and defined the host gene coordinates as the extreme coordinates of all transcripts containing the miRNA (Additional data file 17). Provided gene symbols are UniGene symbols (Build 192) [103].

Genomic coordinates of miRNA stem-loop regions and gene transcripts were based on miRBase (Release 8.1) [17, 18] and the UCSC Genome Browser (hg18) [122] Known Genes and RefSeq Genes tables respectively. The coordinates of mature miRNAs were obtained by mapping the relevant probe sequences to stem-loop sequences and extracting the relative coordinates.

Correlation of miRNAs and proximal Illumina probes

For each miRNA stem-loop region on a given chromosome and strand we extracted all Illumina probes which mapped to the spatial cluster or host gene locus if applicable, or within 50 kb of the stem-loop coordinates. We then calculated all pairwise Pearson correlation coefficients and plotted the correlation matrix as a heatmap, with probes arranged in the order of their genomic coordinates (Additional data file 13). In this representation the spatial information is limited to the order of probes on the chromosomal strand. We therefore included chromosomal plots indicating relative probe positions.

We wanted to ensure that the observed correlations of proximal probes are not solely caused by changes in DNA copy number. Each correlation coefficient was therefore calculated using only those 82 samples for which array CGH data were available, and any samples with evidence for genomic aberrations at either of the two probe loci were excluded. For this purpose, the locus of a miRNA probe was defined as cluster coordinates, host gene coordinates or 50 kb up- and downstream of the stem-loop region as applicable. The locus of Illumina probes was defined as the extreme coordinates of all annotated transcripts or probe coordinates in the case of missing annotation.

Differential expression of miRNA families and predicted targets

We were interested to see whether there was any evidence in our data for miRNAs affecting the expression of genes with conserved seed matches [38, 39].

We showed that a number of miRNAs show differential expression between subtypes, suggesting that changes in miRNA expression may contribute to gene expression changes between subtypes. For the purpose of investigating interactions of miRNAs and putative target genes, we focused on miRNA and mRNA expression changes between subtypes, as the relatively homogeneous gene expression within subtypes provides a theoretical advantage for detecting any such interaction.

Since we were interested in the effect of miRNAs on genes with conserved seed matches, we had to consider the cumulative expression of all miRNAs with identical seed sequence rather than the expression of individual miRNAs. We therefore summarized the miRNA expression data for families of miRNAs sharing the same seed sequence by averaging over the relative expression profiles of all family members.

To reduce the number of statistical tests performed, we only considered 24 candidate miRNA families which showed significant expression changes between subtypes (Kruskal-Wallis adjusted $p < 0.05$). For these miRNAs we extracted genes with conserved seed matches as identified by TargetScanS 3.1 [85, 98].

Since miRNA-mediated changes in gene expression are likely to be small compared to changes due to other mechanisms, we chose a statistical test which does not take into account the size of change: For each subtype contrast and candidate miRNA family we tested (1) for differences in expression of the miRNA family using a non-parametric Wilcoxon rank sum test, and (2) enrichment for up- or down-regulation among changes in median expression of genes with conserved seed matches using Fisher's sign test. (Expression profiles for multiple Illumina probes annotated to the same gene symbol were previously summarized by their median expression profile to avoid spuriously significant results.)

We thus identified 18 instances of differentially expressed miRNAs and simultaneous deregulation of genes with conserved seed matches, nine of which were consistent with the hypothesis of miRNA-mediated gene regulation (results not shown).

Figure Legends

Additional data file 2. Experimental design. A. Data matrix of miRNA expression values (schematic). The 333 rows and 168 columns correspond to probes and samples respectively. Expression values for each sample were obtained from hybridizations to four distinct bead sets (with approximately 90 probes each), carried out in separate wells of 96-well plates. Hybridizations were performed on eight plates, using two plates for each bead set. The allocation of samples between the two plates for a given bead set was kept consistent for all four bead sets. Thus both probes and samples could be ordered according to the plate of origin, partitioning the data matrix into eight blocks corresponding to measurements from distinct plates. Expression values for a representative well on plate 1 for beadset 1 are indicated in grey. B. Heatmap of unnormalized $\log_2$ MFI values for all miRNA probes and all samples. Probes were median centred prior to plotting. C. Heatmap of differences between the probe median for the randomized samples on a given plate and the probe median for all samples on both plates.

Additional data file 3. Preprocessing of miRNA expression data. A. Histograms of $\log_2$ MFI values obtained from wells containing sample material (white) and blank control wells (blue). B. The number of detected probes after filtering was plotted against a range of cutoff values. Probes were removed (filtered) if they did not exceed the chosen cutoff (red) in at least one sample. C, D. Sample quality control. Pearson correlation coefficients for technical replicate samples were plotted against the smaller of the two sample means for (C) cell line technical replicate samples and (D) normal and tumour technical replicate samples. The cutoff used for quality control is indicated by a vertical line. Colours corresponding to sample status are explained in the colour

key. E. Technical sample effects. Pairwise differences between the medians of technical replicate samples were plotted for unnormalized data (black), data normalized based on spike-in controls (blue) and data normalized by sample median centering (red). Dashed lines indicate the maximum difference between the medians of any two samples for unnormalized data (black) and for data normalized using spike-in controls (blue).

Additional data file 4. Between-sample normalization. A. Shown are data normalized based on spike-in controls for the same miRNAs and factors as in Figure 3 in the main text. B. miRNAs and factors with at least one association at adjusted $p < 0.01$ based on data normalized using spike-in controls. All miRNAs thus identified were also identified after sample median centering with the exception of miR-152, which was found to be associated with all three factors at $p < 0.05$ (Additional data file 18). Heatmap colours reflect relative miRNA expression. The expression values for a given sample group of interest were summarized by their mean. Brackets in the left margin indicate members of the same miRNA family. Significance levels are indicated in the right margins: * adjusted $p < 0.05$, ** adjusted $p < 0.01$, *** adjusted $p < 0.001$. Abbreviations for subtype: B = Basal-like, H = HER2+, LA = Luminal A, LB = Luminal B, N = Normal-like.

Additional data file 5. Replicate probes. Pairwise scatter plots of replicate probes after sample quality control, probe filtering and within-plate probe correction (none of the replicated probes were removed due to probe filtering). Scatter plots for one failed probe (miR-224-4) are marked in red.

Additional data file 6. Technical replicate samples. Pairwise scatter plots of technical replicate samples after sample quality control, probe filtering, within-plate probe correction and summarizing replicate probes.

Additional data file 7. qRT-PCR validation. Normalized $\log_2$ MFI values were plotted against $\log_2$-transformed and median-corrected measurements obtained by qRT-PCR.

Additonal data file 8. Gene expression heatmap for 82 classified samples and 75 of the 80 intrinsic genes included in Figure 2 of [76]. Expression values are based on Illumina data when available, and Agilent data otherwise. The two data sets were normalized as described. Missing values in the Agilent data are indicated in white. Samples were ordered according to molecular subtype (see colour key). The heatmap does not present a hierarchical clustering but merely illustrates differences in gene expression. A. Luminal/ER+ gene cluster. B. ERBB2 and GRB7-containing cluster. C. Interferon-regulated cluster including STAT1. D. Basal epithelial cluster. E. Proliferation cluster.

Additional data file 9. Pairwise comparison of Kaplan-Meier survival curves for 74 classified samples with available follow up data (21 Basal-like, 7 HER2+, 25 Luminal A, 10 Luminal B, 11 Normal-like). A non-parametric log rank test was used to assess differences in clinical outcome.

Additional data file 10. miRNA processing genes show differential expression according to subtype, and ER status. Shown are boxplots of $\log_2$ expression for *DGCR8*, *DICER1*, *DROSHA*

(*RNASEN*), *AGO1* (*EIF2C1*), *AGO2* (*EIF2C2*), *AGO3* (*EIF2C3*) and *AGO4* (*EIF2C4*). The data were obtained for 58 samples classified according to subtype (17 Basal-like, 5 HER2+, 18 Luminal A, 8 Luminal B, 10 Normal-like) and 99 samples with known ER status (31 ER-, 68 ER+). We only included Illumina probes not mapping to introns and which could be detected at $\log_2$ expression 6 in at least one sample. Differential expression was assessed using a non-parametric Kruskal-Wallis test (subtype) and Wilcoxon rank sum test (ER status).

Additional data file 11. miRNA processing genes show differential expression according to ER status in publicly available data sets. Shown are boxplots of normalized gene expression units for each candidate gene that showed differential expression (Student's t-test $p < 0.001$). Data were obtained from the cancer microarray database ONCOMINE [98], and differential expression was assessed using Student's t-test. Each row of plots corresponds to a unique gene; data obtained from different studies are separated by a solid vertical black line. For each data set the number of ER negative (blue) and ER positive (yellow) samples is included in the lower figure margin. The first authors of the relevant publications are included in the plot title.

Additional data file 12. Correlation of proximal miRNAs. Pearson correlation coefficients for mature miRNAs mapping to the same chromosome and strand were plotted against decreasing ranks of pairwise distances. Diamonds represent a moving average over five correlation coefficients. The absolute distance is plotted in blue and indicated on the right y-axis. Distance 50 kb is indicated by a vertical red line.

Additional data file 13. Correlation of miRNA and Illumina probes. Heatmap of

Pearson correlation coefficients (accounting for DNA copy number changes as described) between miRNA probes and selected Illumina probes on the same chromosome and strand. Blank entries are due to missing DNA copy number information. Probes are arranged in genomic order. Black boxes indicate clusters of adjacent probes less than 50 kb apart. Green boxes indicate clusters of probes mapping to the same host gene. Mature miRNAs included in multiple stem-loops are indicated in blue. Relative genomic probe positions are marked as white bars on the chromosomal plot below each heatmap.

Additional data file 14. Model-based discriminant analysis for Basal-like and Luminal A tumours. A. SSP molecular subtype classification based on the Affymetrix gene expression data for normal breast and breast tumour samples in [56, 85]. Spearman correlations with the five subtype centroids are shown for all 14 samples. The solid horizontal black line indicates the minimum correlation required for subtype assignment. If the minimal correlation with a subtype centroid was achieved, the classification was made using the centroid with highest Spearman correlation. B. Shown are class posterior probabilities for 16 Basal-like and 15 Luminal A tumours in the training set (using all detected 138 miRNAs); and three Basal-like and two Luminal A tumours in the test set (using the 77 detected miRNAs in common with the training set). Red and blue indicate the posterior probability of belonging to the Basal-like and Luminal A subtype respectively. Plotting characters indicate the gene expression based subtype classification with squares and triangles representing Basal-like and Luminal A samples respectively. Samples were assigned to the class with posterior probability greater than 0.5 (solid horizontal black line).
