# Supplementary figures and images for: MicroRNA expression profiling of human breast cancer identifies new markers of tumor subtype
Source: Genome Biol. 2007 Oct 8;8(10):R214. doi: 10.1186/gb-2007-8-10-r214 (PMC2246288; doi:10.1186/gb-2007-8-10-r214)

**A**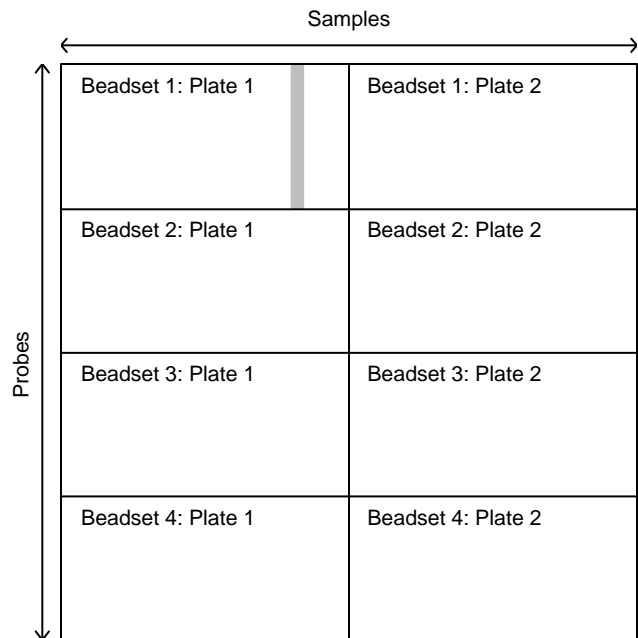**B**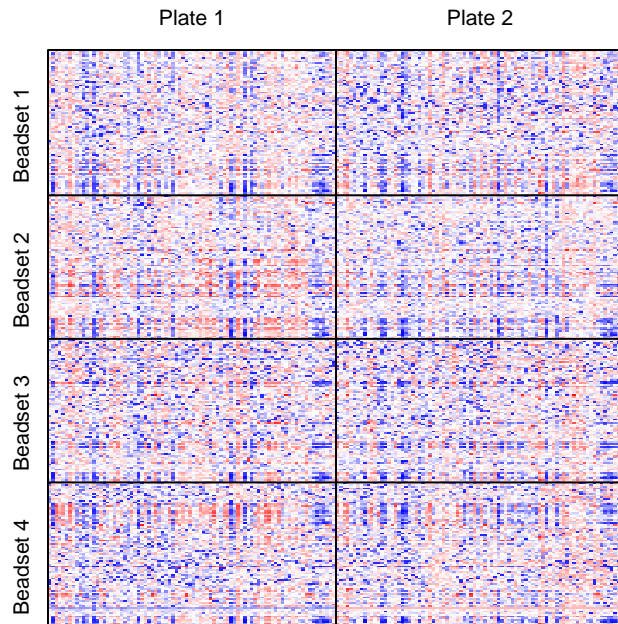**C**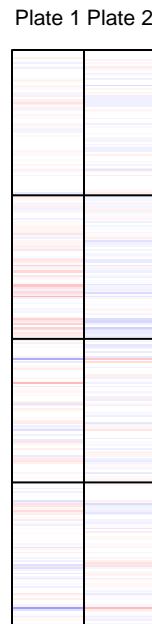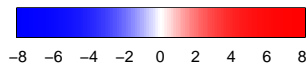

Supplement: Additional data file 2 — A. Data matrix of miRNA expression values (schematic). The 333 rows and 168 columns correspond to probes and samples respectively. Expression values for each sample were obtained from hybridizations to four distinct bead sets (with approximately 90 probes each), carried out in separate wells of 96-well plates. Hybridizations were performed on eight plates, using two plates for each bead set. The allocation of samples between the two plates for a given bead set was kept consistent for all four bead sets. Thus both probes and samples could be ordered according to the plate of origin, partitioning the data matrix into eight blocks corresponding to measurements from distinct plates. Expression values for a representative well on plate 1 for beadset 1 are indicated in grey. B. Heatmap of unnormalized log2 MFI values for all miRNA probes and all samples. Probes were median centred prior to plotting. C. Heatmap of differences between the probe median for the randomized samples on a given plate and the probe median for all samples on both plates. [file gb-2007-8-10-r214-S2.pdf]

**A**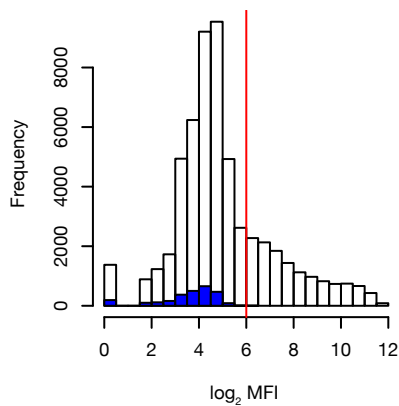**B**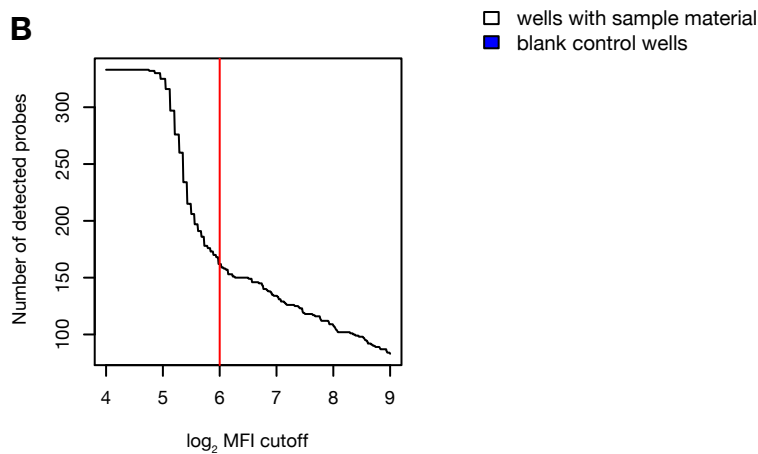**C**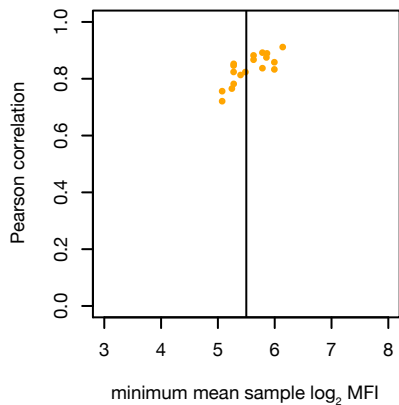**D**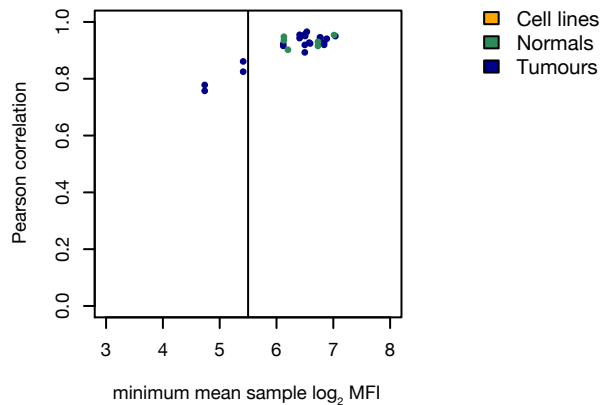**E**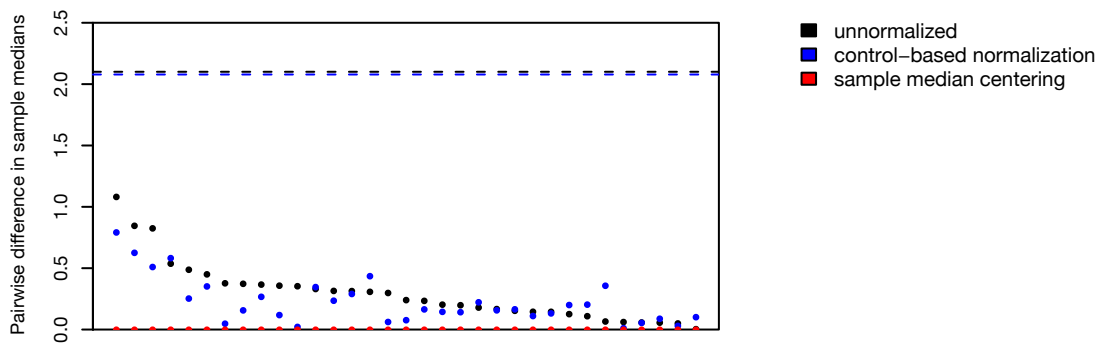

Supplement: Additional data file 3 — A. Histograms of log2 MFI values obtained from wells containing sample material (white) and blank control wells (blue). B. The number of detected probes after filtering was plotted against a range of cutoff values. Probes were removed (filtered) if they did not exceed the chosen cutoff (red) in at least one sample. C, D. Sample quality control. Pearson correlation coefficients for technical replicate samples were plotted against the smaller of the two sample means for (C) cell line technical replicate samples and (D) normal and tumor technical replicate samples. The cutoff used for quality control is indicated by a vertical line. Colours corresponding to sample status are explained in the colour key. E. Technical sample effects. Pairwise differences between the medians of technical replicate samples were plotted for unnormalized data (black), data normalized based on spike-in controls (blue) and data normalized by sample median centering (red). Dashed lines indicate the maximum difference between the medians of any two samples for unnormalized data (black) and for data normalized using spike-in controls (blue). [file gb-2007-8-10-r214-S3.pdf]

**A**

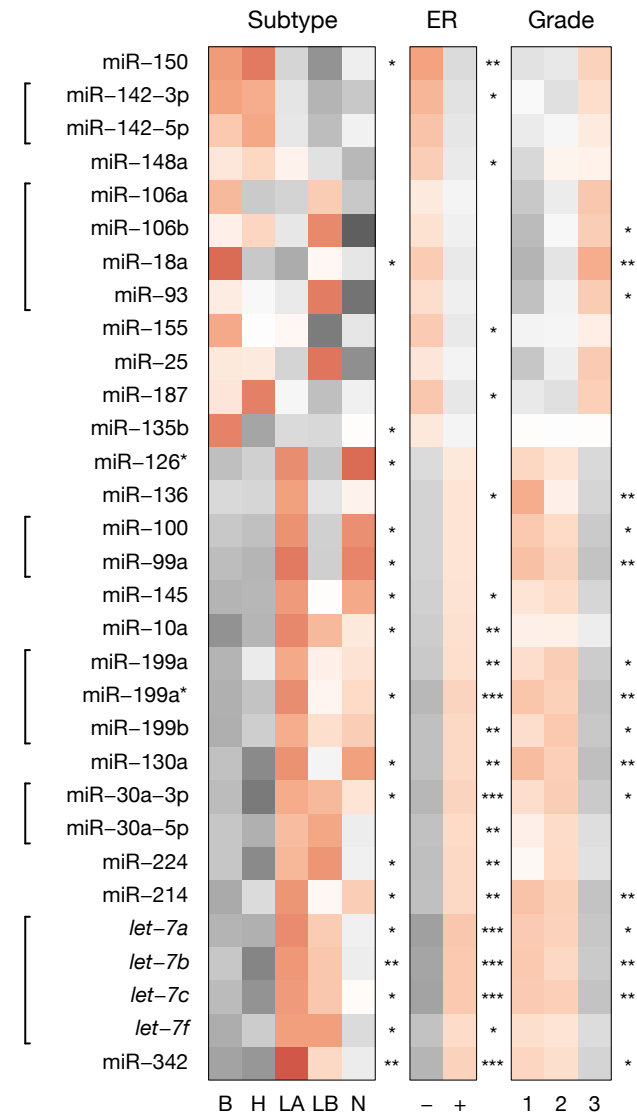

**B**

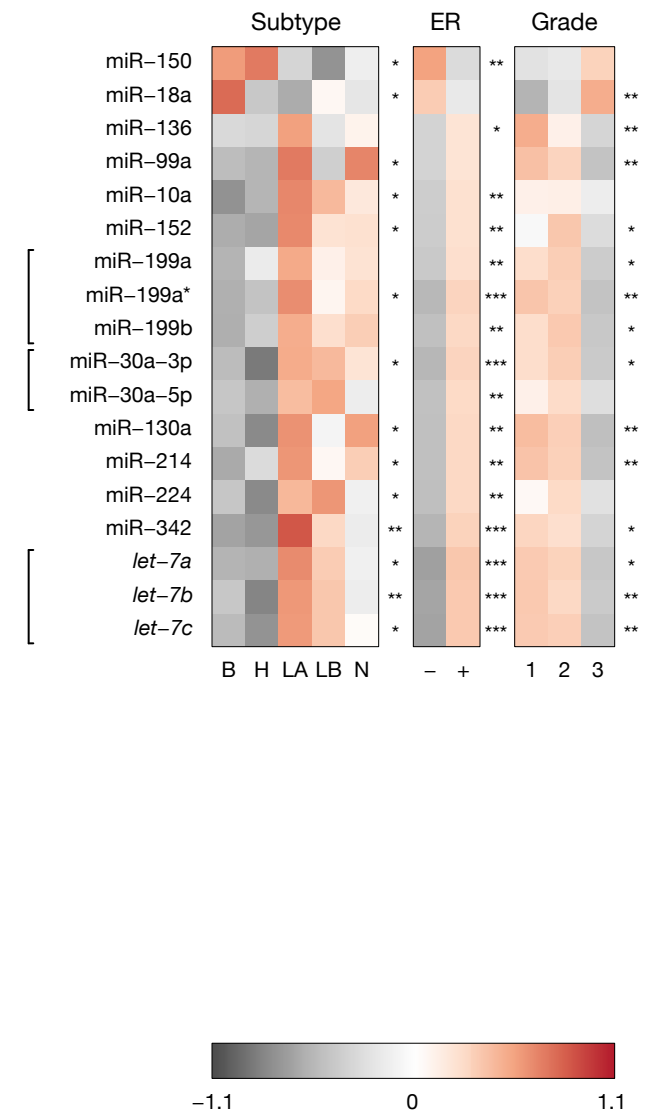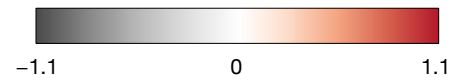

Supplement: Additional data file 4 — Between-sample normalization. A. Shown are data normalized based on spike-in controls for the same miRNAs and factors as in Figure 3 in the main text. B. miRNAs and factors with at least one association at adjusted p < 0.01 based on data normalized using spike-in controls. All miRNAs thus identified were also identified after sample median centering with the exception of miR-152, which was found to be associated with all three factors at p < 0.05 (Additional data file 18). Heatmap colours reflect relative miRNA expression. The expression values for a given sample group of interest were summarized by their mean. Brackets in the left margin indicate members of the same miRNA family. Significance levels are indicated in the right margins: * adjusted p < 0.05, ** adjusted p < 0.01, *** adjusted p < 0.001. Abbreviations for subtype: B = Basal-like, H = HER2+, LA = Luminal A, LB = Luminal B, N = Normal-like. [file gb-2007-8-10-r214-S4.pdf]

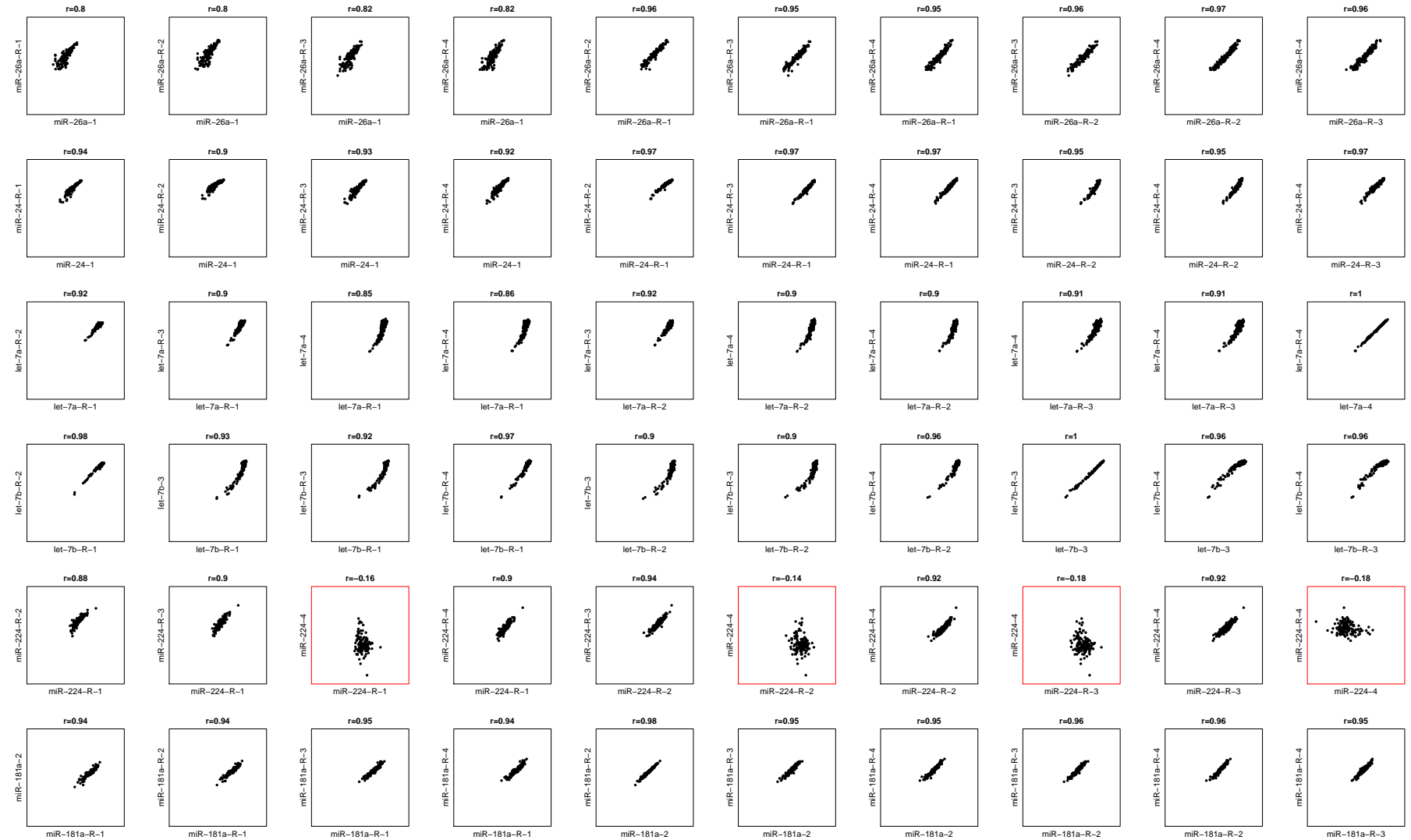

Supplement: Additional data file 5 — Pairwise scatter plots of replicate probes after sample quality control, probe filtering and within-plate probe correction (none of the replicated probes were removed due to probe filtering). Scatter plots for one failed probe (miR-224-4) are marked in red. [file gb-2007-8-10-r214-S5.pdf]

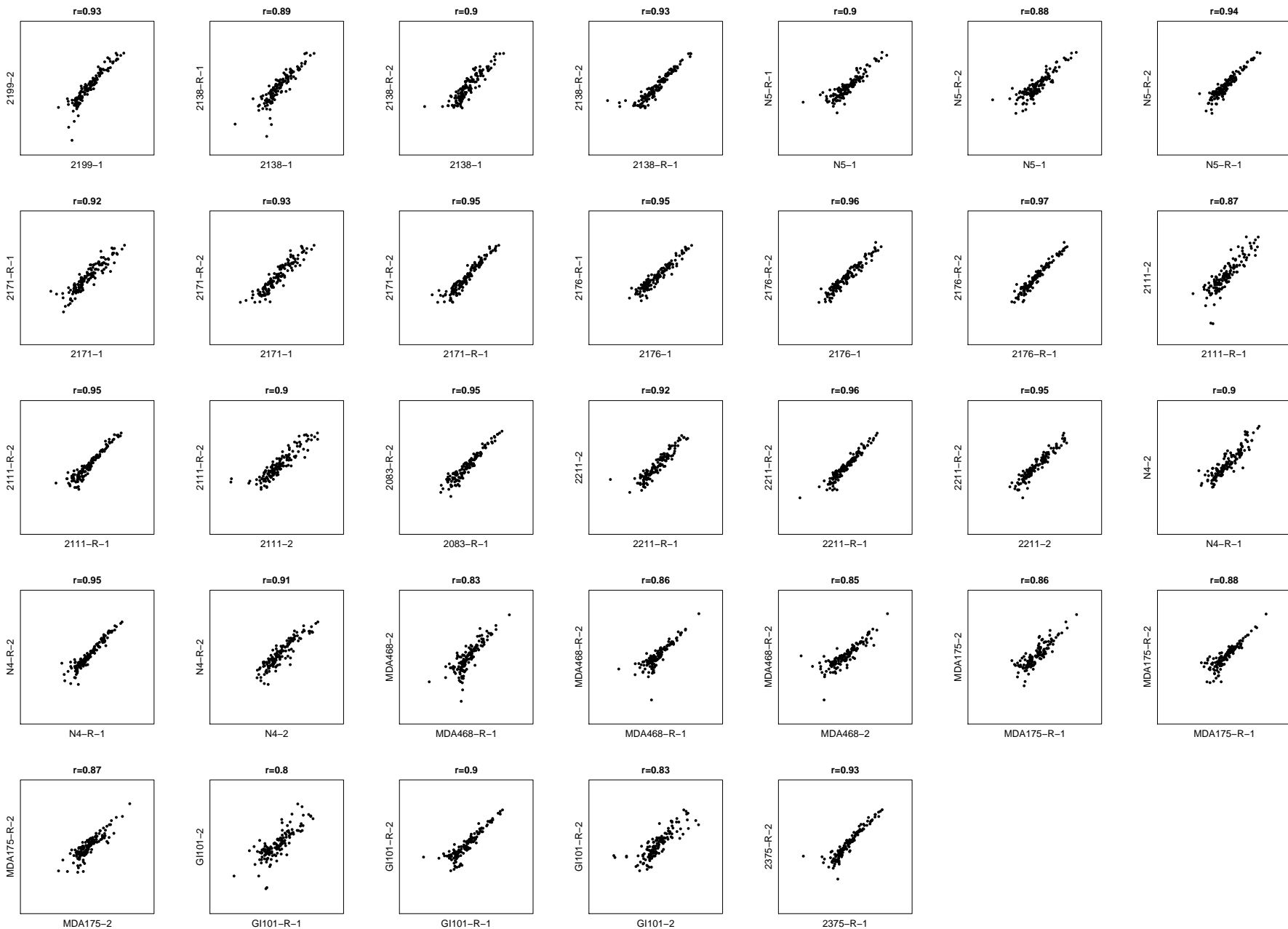

Supplement: Additional data file 6 — Pairwise scatter plots of technical replicate samples after sample quality control, probe filtering, within-plate probe correction and summarizing replicate probes. [file gb-2007-8-10-r214-S6.pdf]

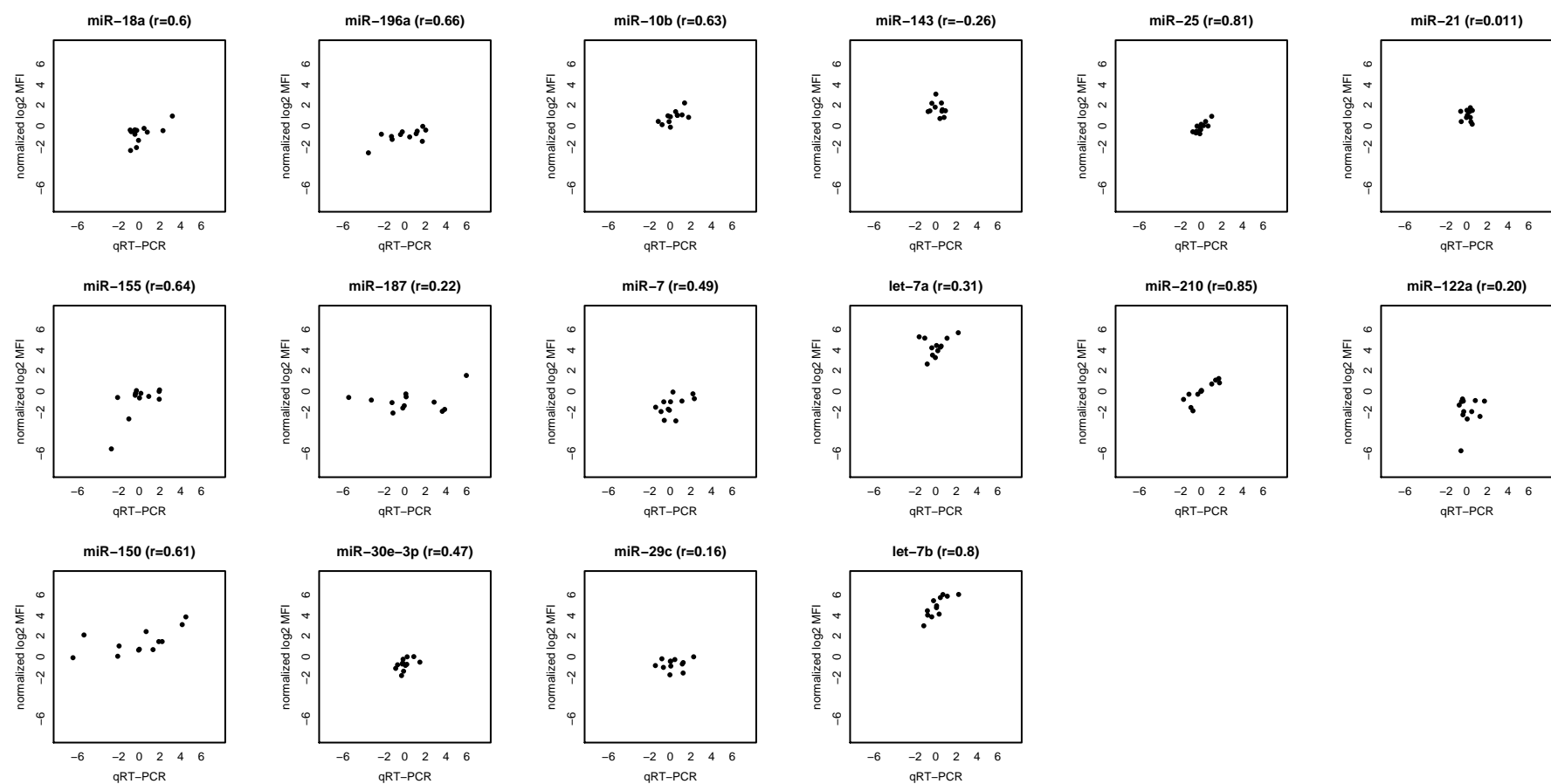

Supplement: Additional data file 7 — Normalized log2 MFI values were plotted against log2-transformed and median-corrected measurements obtained by qRT-PCR [file gb-2007-8-10-r214-S7.pdf]

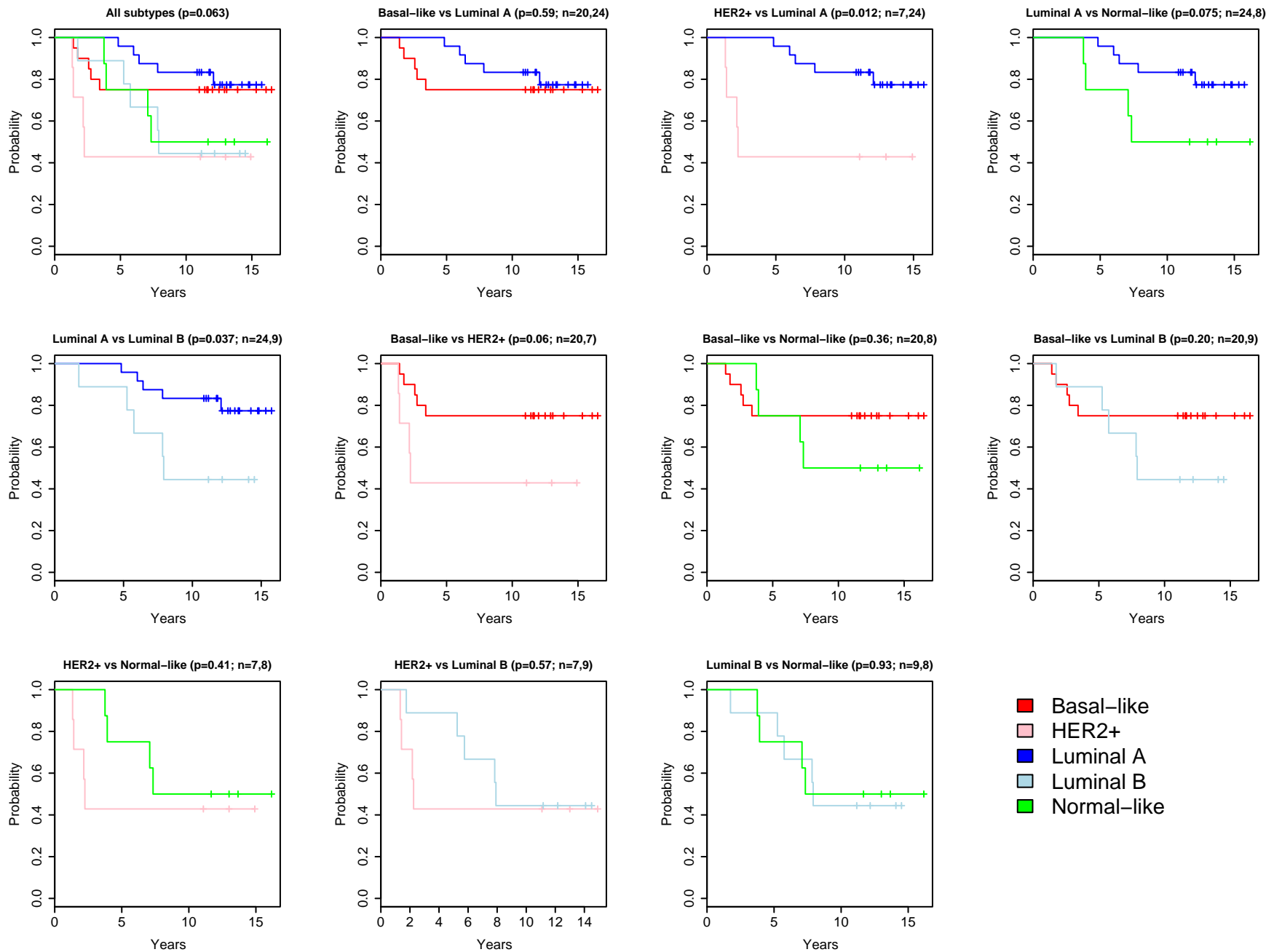

Supplement: Additional data file 9 — Pairwise comparison of Kaplan-Meier survival curves for 74 classified samples with available follow up data (21 Basal-like, 7 HER2+, 25 Luminal A, 10 Luminal B, 11 Normal-like). A non-parametric log rank test was used to assess differences in clinical outcome. [file gb-2007-8-10-r214-S9.pdf]

## AGO1 (EIF2C1)

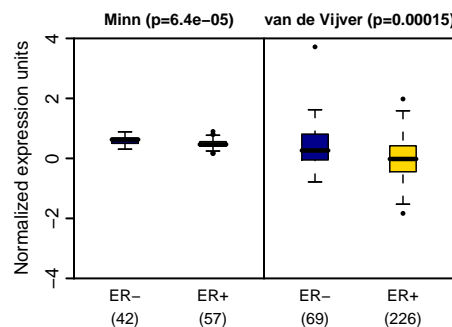

## AGO2 (EIF2C2)

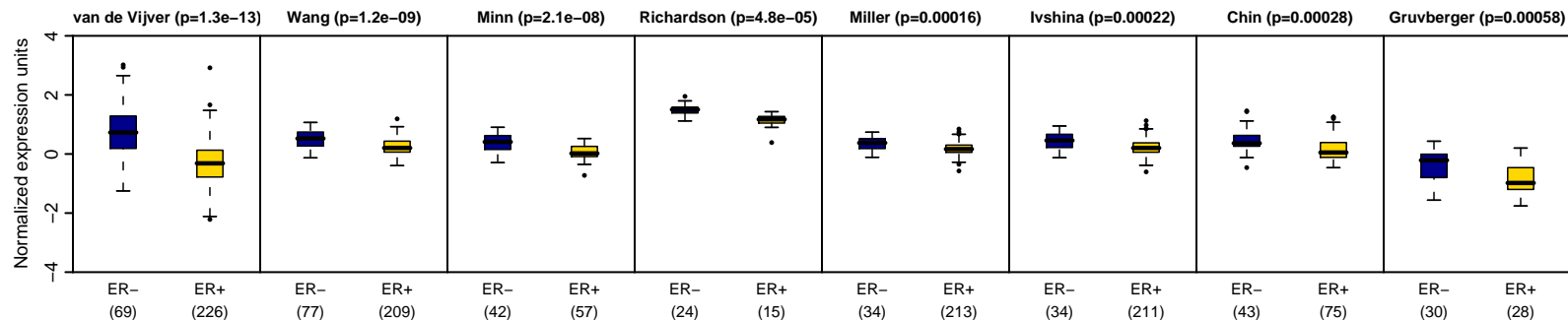

## DICER1

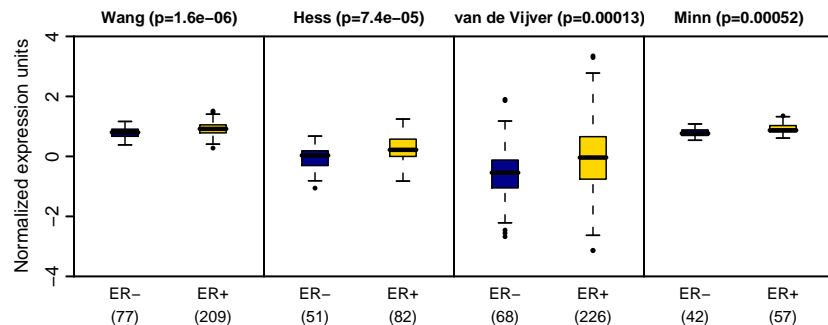

## DROSHA (RNASEN)

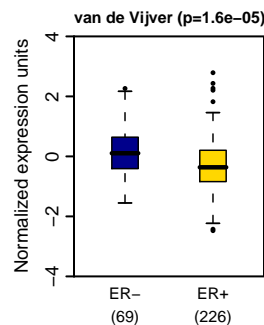

ER-  
ER+

Supplement: Additional data file 11 — Shown are boxplots of normalized gene expression units for each candidate gene that showed differential expression (Student's t-test p < 0.001). Data were obtained from the cancer microarray database ONCOMINE [98], and differential expression was assessed using Student's t-test. Each row of plots corresponds to a unique gene; data obtained from different studies are separated by a solid vertical black line. For each data set the number of ER negative (blue) and ER positive (yellow) samples is included in the lower figure margin. The first authors of the relevant publications are included in the plot title. [file gb-2007-8-10-r214-S11.pdf]

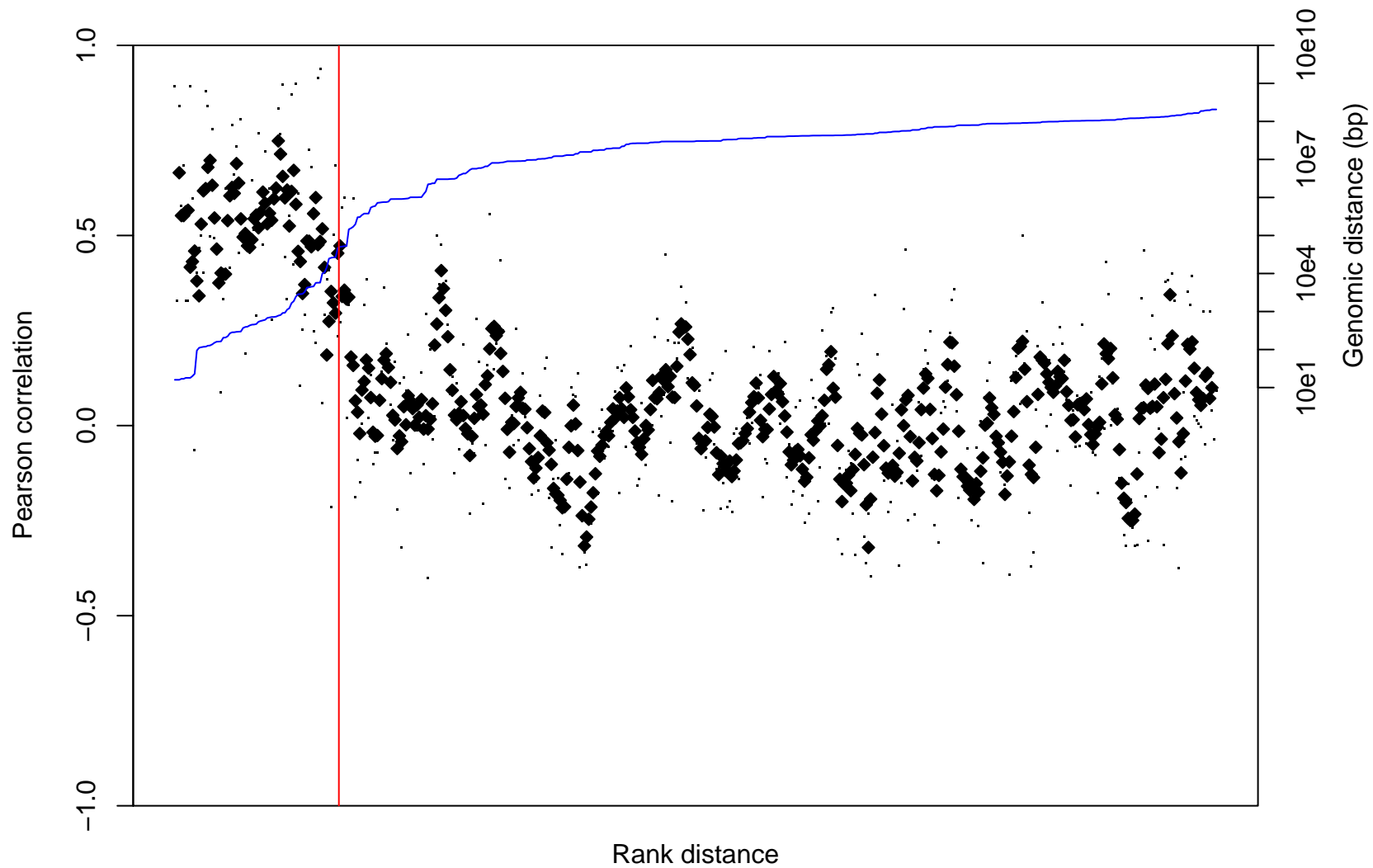

Supplement: Additional data file 12 — Pearson correlation coefficients for mature miRNAs mapping to the same chromosome and strand were plotted against decreasing ranks of pairwise distances. Diamonds represent a moving average over five correlation coefficients. The absolute distance is plotted in blue and indicated on the right y-axis. Distance 50 kb is indicated by a vertical red line. [file gb-2007-8-10-r214-S12.pdf]

**A**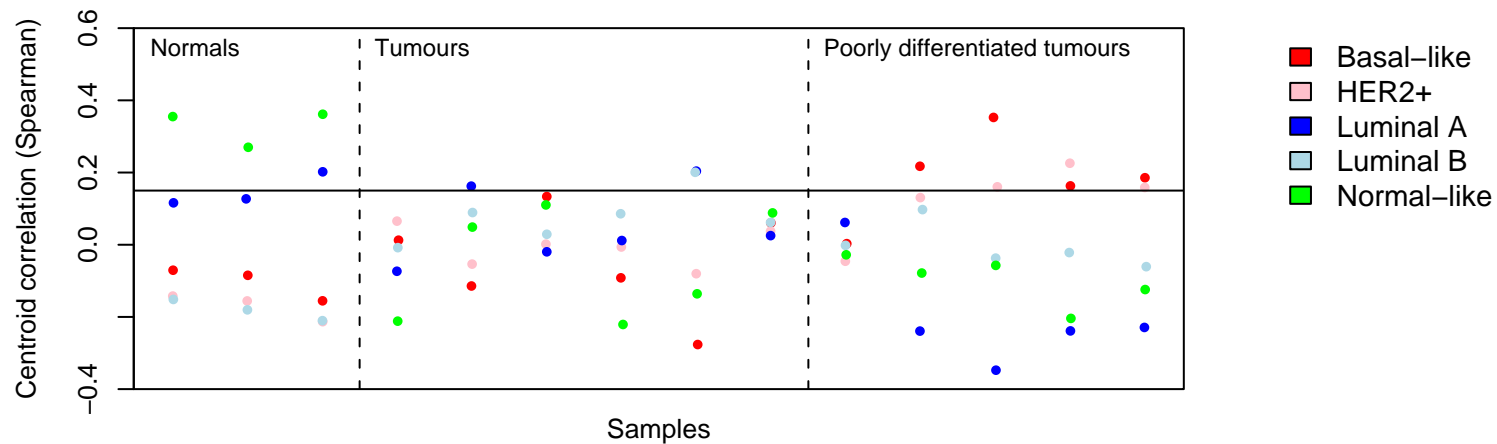**B**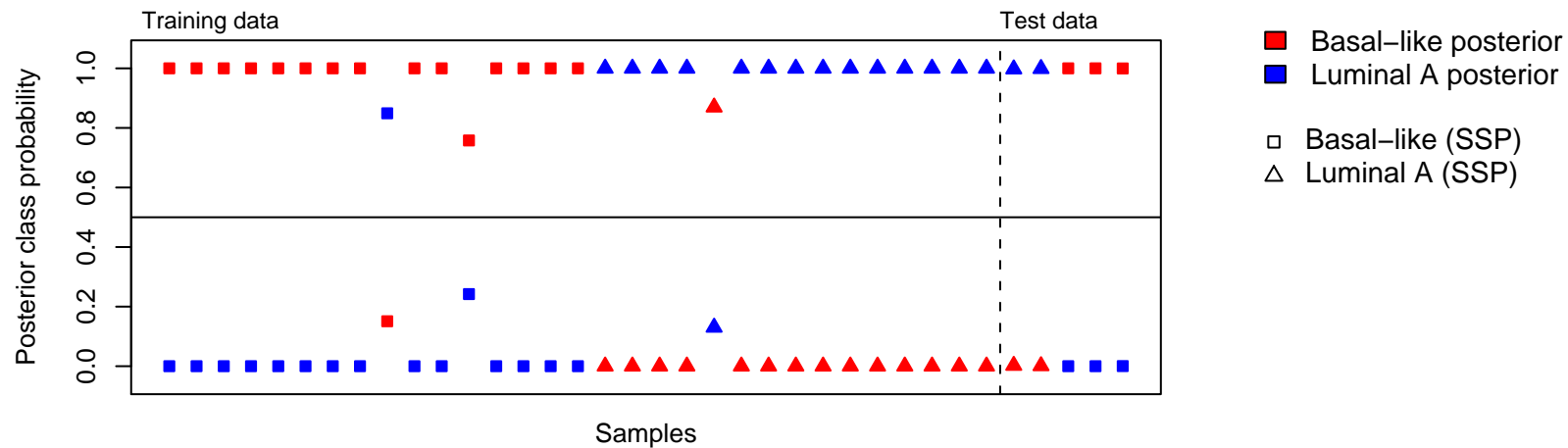

Supplement: Additional data file 14 — SSP molecular subtype classification based on the Affymetrix gene expression data for normal breast and breast tumor samples in [56,85]. Spearman correlations with the five subtype centroids are shown for all 14 samples. The solid horizontal black line indicates the minimum correlation required for subtype assignment. If the minimal correlation with a subtype centroid was achieved, the classification was made using the centroid with highest Spearman correlation. B. Shown are class posterior probabilities for 16 Basal-like and 15 Luminal A tumors in the training set (using all detected 138 miRNAs); and three Basal-like and two Luminal A tumors in the test set (using the 77 detected miRNAs in common with the training set). Red and blue indicate the posterior probability of belonging to the Basal-like and Luminal A subtype respectively. Plotting characters indicate the gene expression based subtype classification with squares and triangles representing Basal-like and Luminal A samples respectively. Samples were assigned to the class with posterior probability greater than 0.5 (solid horizontal black line). [file gb-2007-8-10-r214-S14.pdf]
