# Supplementary material for: MicroRNA expression profiling of human breast cancer identifies new markers of tumor subtype
Source: Genome Biol. 2007 Oct 8;8(10):R214. doi: 10.1186/gb-2007-8-10-r214 (PMC2246288; doi:10.1186/gb-2007-8-10-r214)
Supplement: Additional data file 8 — Expression values are based on Illumina data when available, and Agilent data otherwise. The two data sets were normalized as described. Missing values in the Agilent data are indicated in white. Samples were ordered according to molecular subtype (see colour key). The heatmap does not present a hierarchical clustering but merely illustrates differences in gene expression. A. Luminal/ER+ gene cluster. B. ERBB2 and GRB7-containing cluster. C. Interferon-regulated cluster including STAT1. D. Basal epithelial cluster. E. Proliferation cluster. [file gb-2007-8-10-r214-S8.pdf]

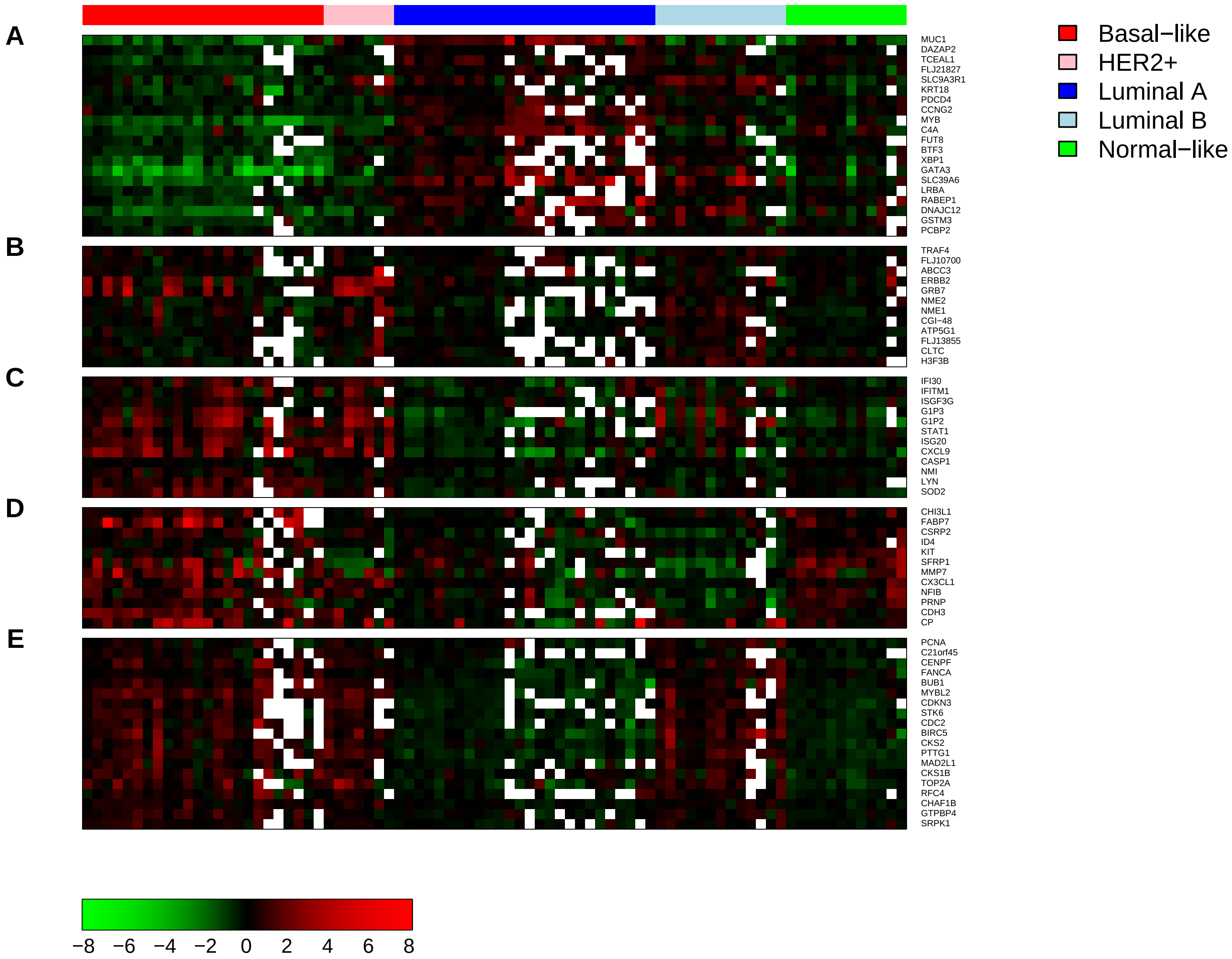
A
B
C
D
E
Basal−like
HER2+
Luminal A
Luminal B
Normal−like
MUC1
DAZAP2
TCEAL1
FLJ21827
SLC9A3R1
KRT18
PDCD4
CCNG2
MYB
C4A
FUT8
BTF3
XBP1
GATA3
SLC39A6
LRBA
RABEP1
DNAJC12
GSTM3
PCBP2
TRAF4
FLJ10700
ABCC3
ERBB2
GRB7
NME2
NME1
CGI−48
ATP5G1
FLJ13855
CLTC
H3F3B
IFI30
IFITM1
ISGF3G
G1P3
G1P2
STAT1
ISG20
CXCL9
CASP1
NMI
LYN
SOD2
CHI3L1
FABP7
CSRP2
ID4
KIT
SFRP1
MMP7
CX3CL1
NFIB
PRNP
CDH3
CP
PCNA
C21orf45
CENPF
FANCA
BUB1
MYBL2
CDKN3
STK6
CDC2
BIRC5
CKS2
PTTG1
MAD2L1
CKS1B
TOP2A
RFC4
CHAF1B
GTPBP4
SRPK1
−8
−6
−4
−2
0
2
4
6
8
