# Supplementary material for: MicroRNA expression profiling of human breast cancer identifies new markers of tumor subtype
Source: Genome Biol. 2007 Oct 8;8(10):R214. doi: 10.1186/gb-2007-8-10-r214 (PMC2246288; doi:10.1186/gb-2007-8-10-r214)
Supplement: Additional data file 10 — Shown are boxplots of log2 expression for DGCR8, DICER1, DROSHA (RNASEN), AGO1 (EIF2C1), AGO2 (EIF2C2), AGO3 (EIF2C3) and AGO4 (EIF2C4). The data were obtained for 58 samples classified according to subtype (17 Basal-like, 5 HER2+, 18 Luminal A, 8 Luminal B, 10 Normal-like) and 99 samples with known ER status (31 ER-, 68 ER+). We only included Illumina probes not mapping to introns and which could be detected at log2 expression 6 in at least one sample. Differential expression was assessed using a non-parametric Kruskal-Wallis test (subtype) and Wilcoxon rank sum test (ER status). [file gb-2007-8-10-r214-S10.pdf]

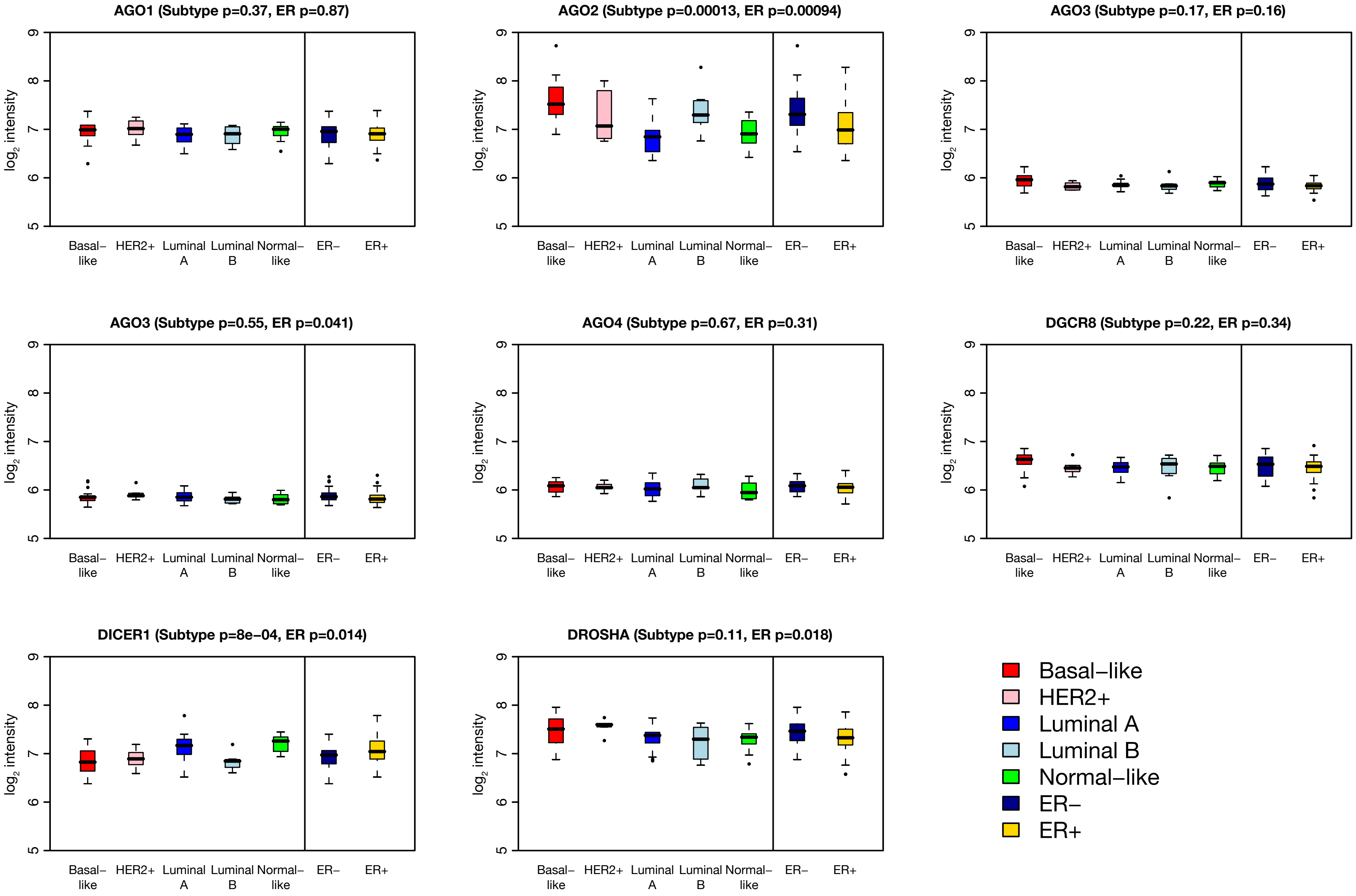
AGO1 (Subtype p=0.37, ER p=0.87)
AGO2 (Subtype p=0.00013, ER p=0.00094)
AGO3 (Subtype p=0.17, ER p=0.16)
AGO3 (Subtype p=0.55, ER p=0.041)
AGO4 (Subtype p=0.67, ER p=0.31)
DGCR8 (Subtype p=0.22, ER p=0.34)
DICER1 (Subtype p=8e−04, ER p=0.014)
DROSHA (Subtype p=0.11, ER p=0.018)
log_2 intensity
5
6
7
8
9
Basal−like
HER2+
Luminal A
Luminal B
Normal−like
ER−
ER+
