# Supplementary material for: MicroRNA expression profiling of human breast cancer identifies new markers of tumor subtype
Source: Genome Biol. 2007 Oct 8;8(10):R214. doi: 10.1186/gb-2007-8-10-r214 (PMC2246288; doi:10.1186/gb-2007-8-10-r214)
Supplement: Additional data file 13 — Heatmap of Pearson correlation coefficients (accounting for DNA copy number changes as described) between miRNA probes and selected Illumina probes on the same chromosome and strand. Blank entries are due to missing DNA copy number information. Probes are arranged in genomic order. Black boxes indicate clusters of adjacent probes less than 50 kb apart. Green boxes indicate clusters of probes mapping to the same host gene. Mature miRNAs included in multiple stem-loops are indicated in blue. Relative genomic probe positions are marked as white bars on the chromosomal plot below each heatmap. [file gb-2007-8-10-r214-S13.pdf]

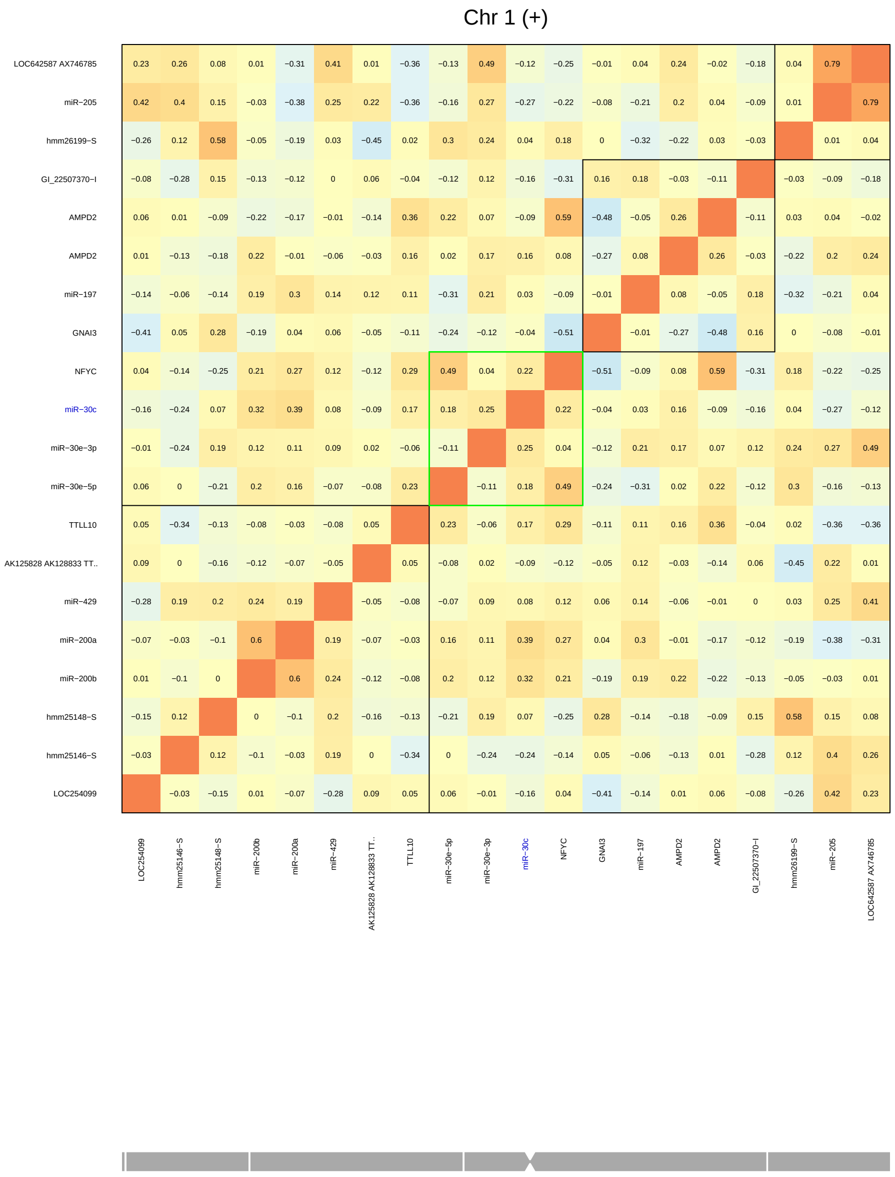
Chr 1 (+)
LOC642587 AX746785
miR−205
hmm26199−S
GI_22507370−I
AMPD2
AMPD2
miR−197
GNAI3
NFYC
miR−30c
miR−30e−3p
miR−30e−5p
TTLL10
AK125828 AK128833 TT..
miR−429
miR−200a
miR−200b
hmm25148−S
hmm25146−S
LOC254099

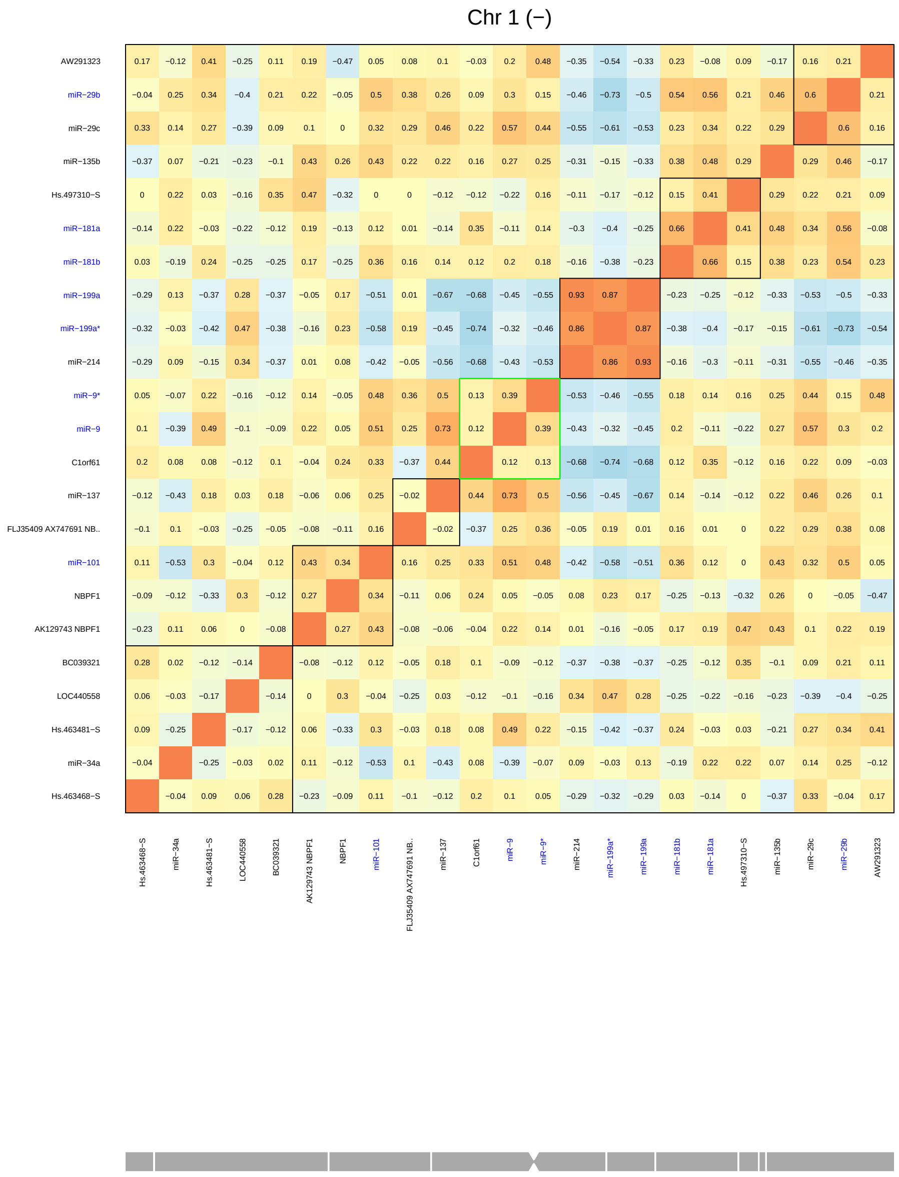
Chr 1 (−)
AW291323
miR−29b
miR−29c
miR−135b
Hs.497310−S
miR−181a
miR−181b
miR−199a
miR−199a*
miR−214
miR−9*
miR−9
C1orf61
miR−137
FLJ35409 AX747691 NB..
miR−101
NBPF1
AK129743 NBPF1
BC039321
LOC440558
Hs.463481−S
miR−34a
Hs.463468−S

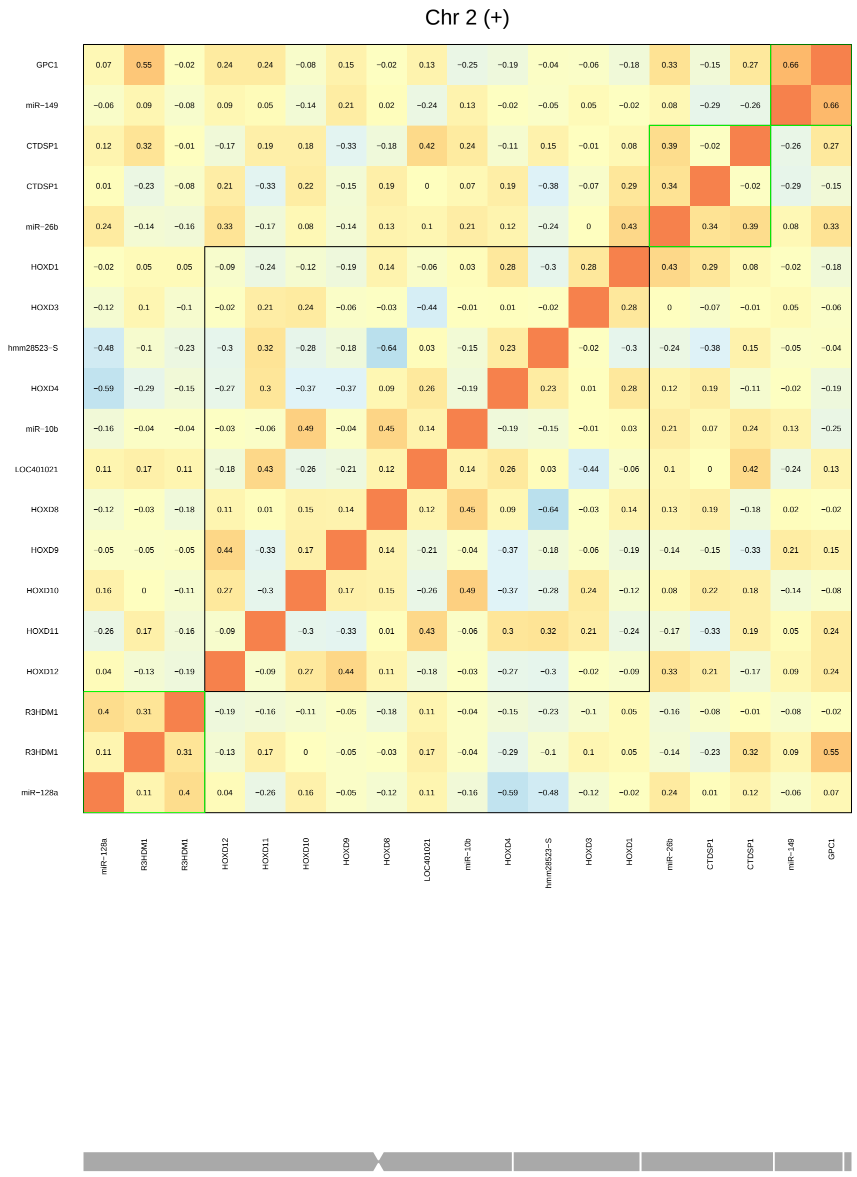
Chr 2 (+)
GPC1
miR−149
CTDSP1
CTDSP1
miR−26b
HOXD1
HOXD3
hmm28523−S
HOXD4
miR−10b
LOC401021
HOXD8
HOXD9
HOXD10
HOXD11
HOXD12
R3HDM1
R3HDM1
miR−128a

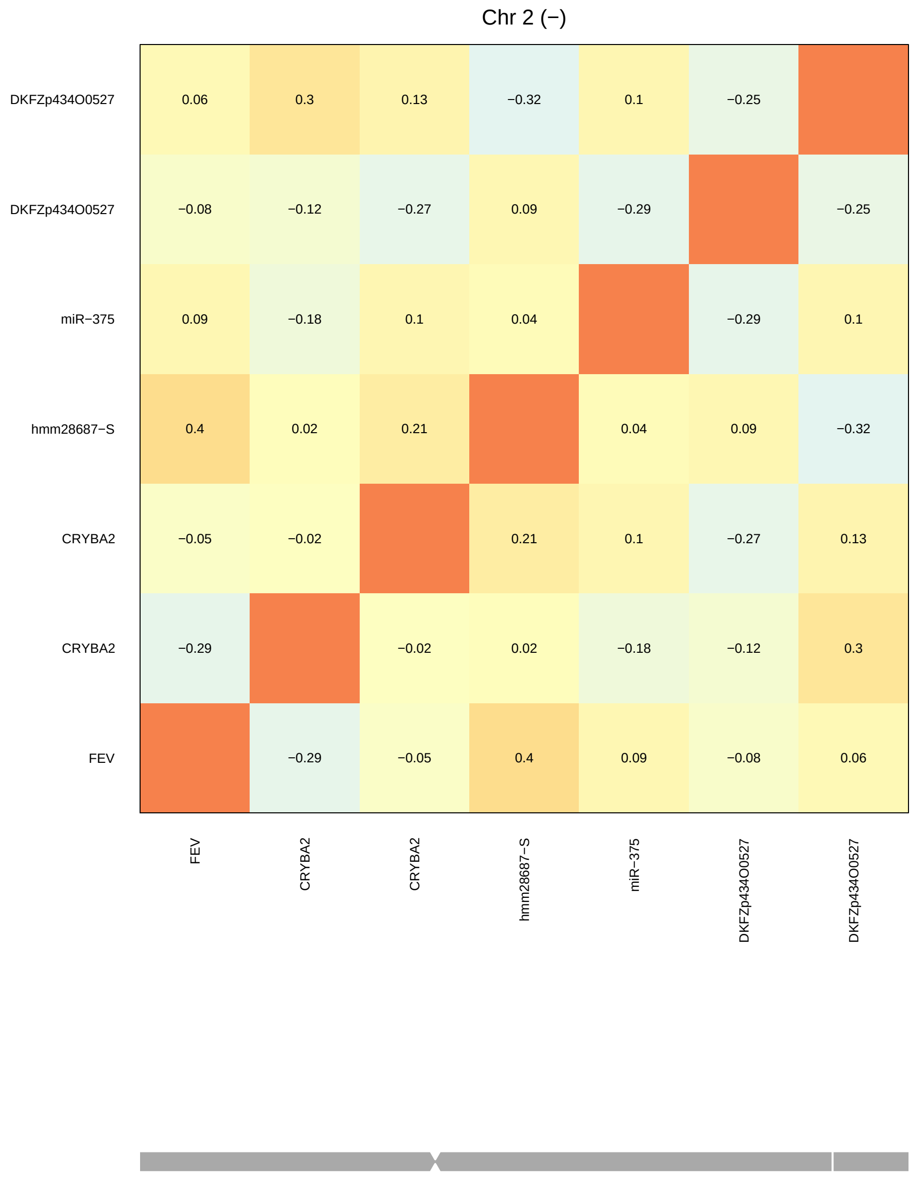
Chr 2 (−)
DKFZp434O0527
DKFZp434O0527
miR−375
hmm28687−S
CRYBA2
CRYBA2
FEV

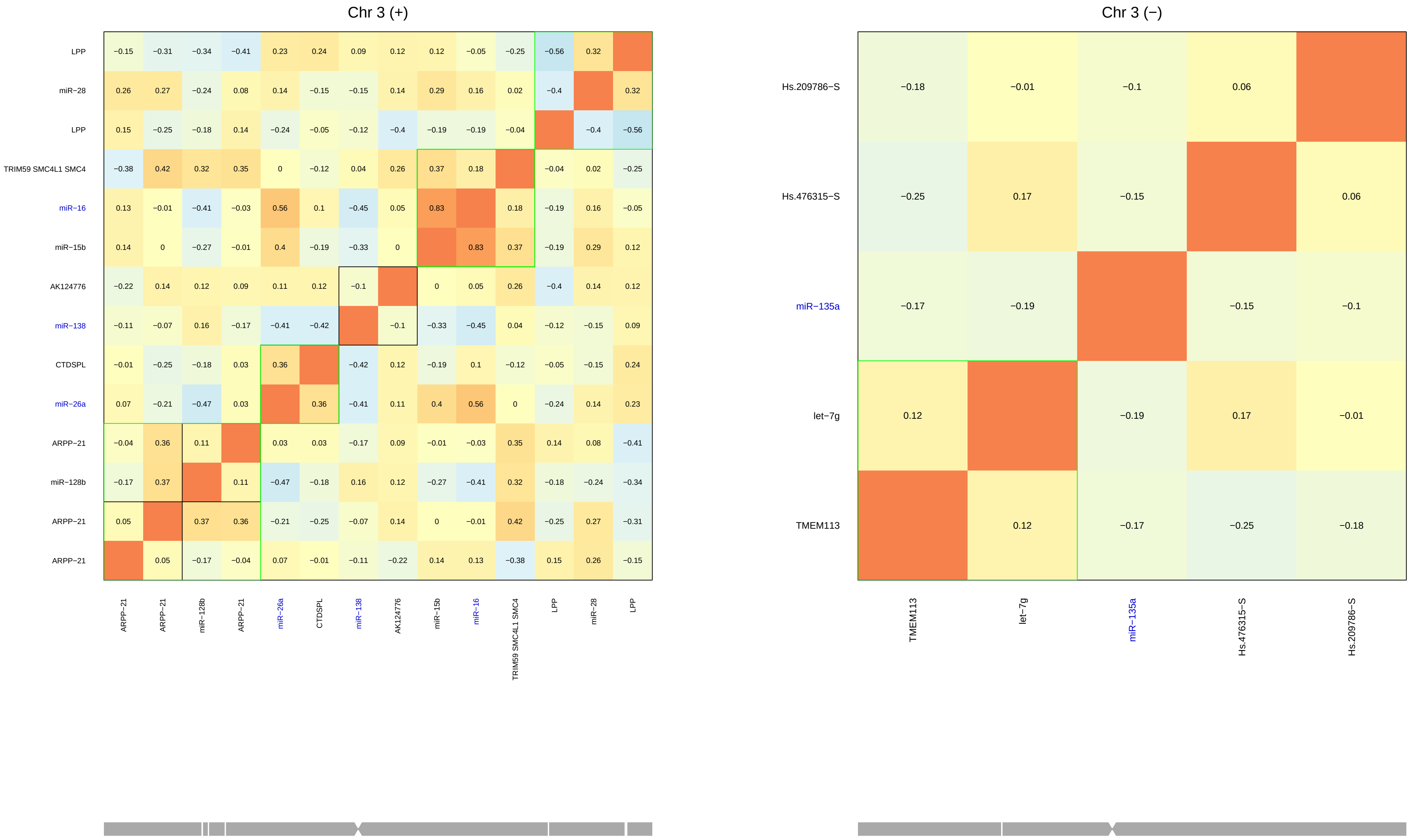

Chr 3 (+)
LPP
miR−28
LPP
TRIM59 SMC4L1 SMC4
miR−16
miR−15b
AK124776
miR−138
CTDSPL
miR−26a
ARPP−21
miR−128b
ARPP−21
ARPP−21
Chr 3 (−)
Hs.209786−S
Hs.476315−S
miR−135a
let−7g
TMEM113

Chr 4 (+)

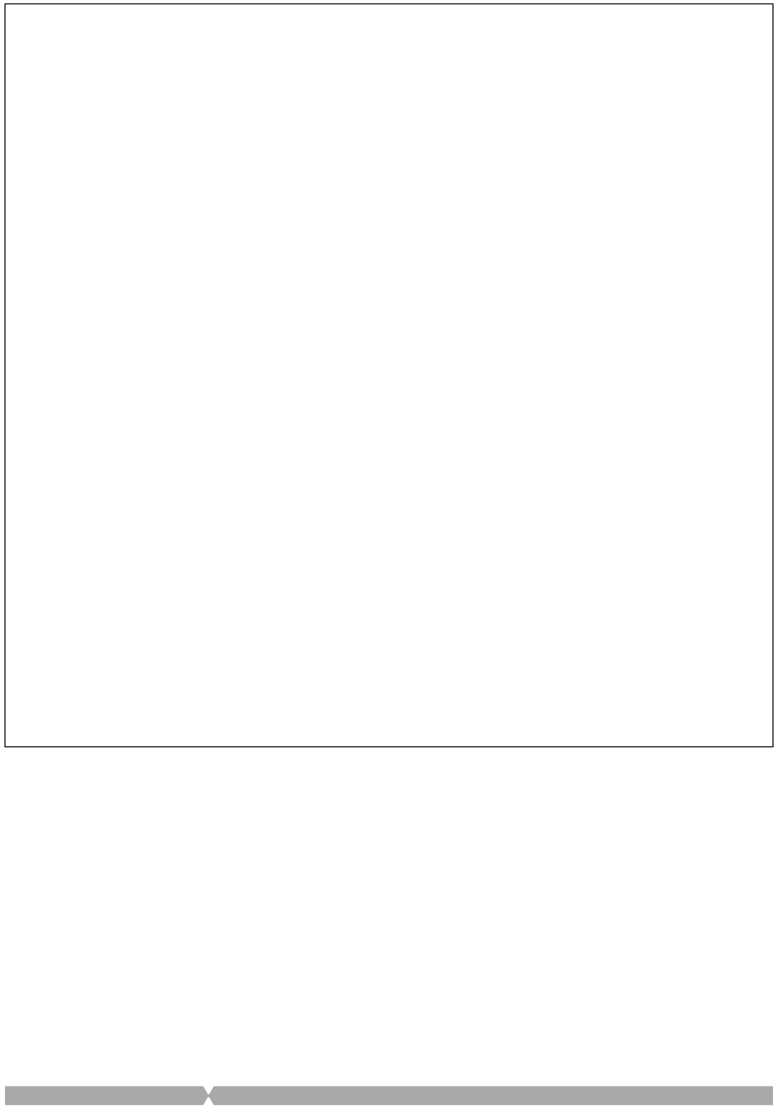

Chr 4 (−)

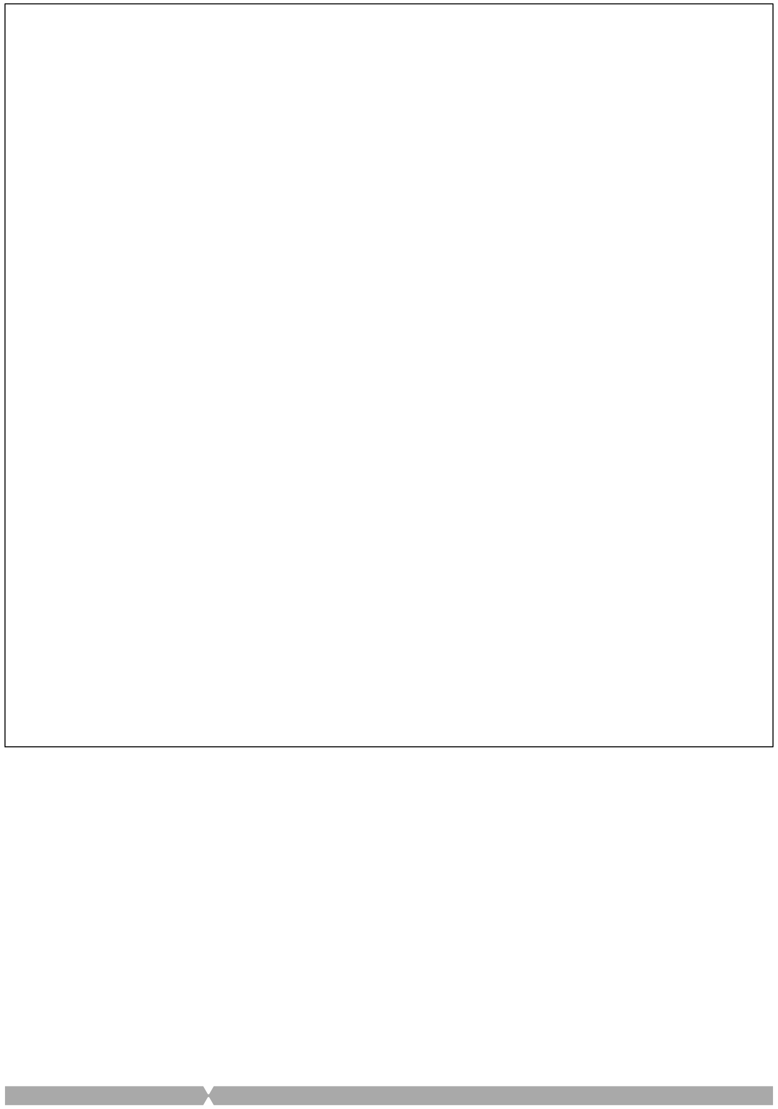

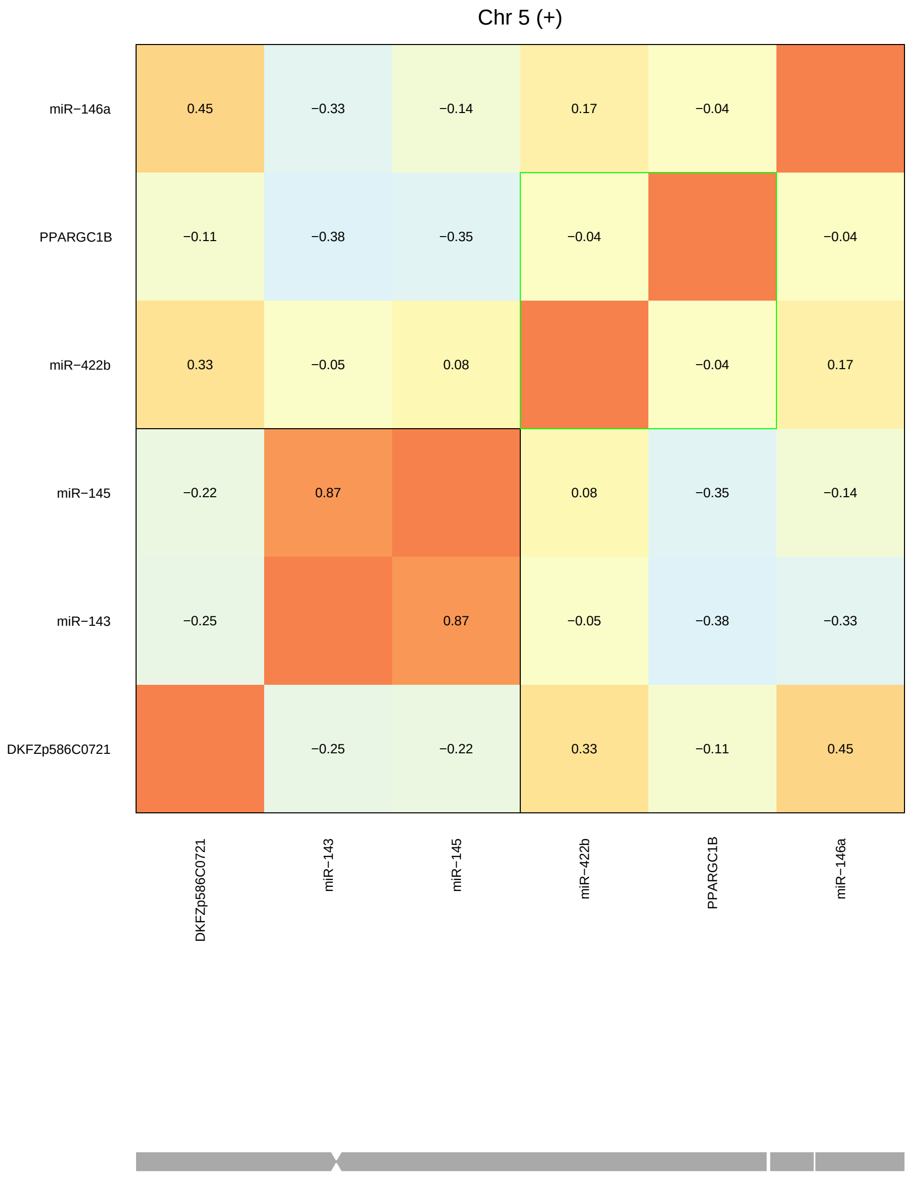
Chr 5 (+)
miR−146a
PPARGC1B
miR−422b
miR−145
miR−143
DKFZp586C0721

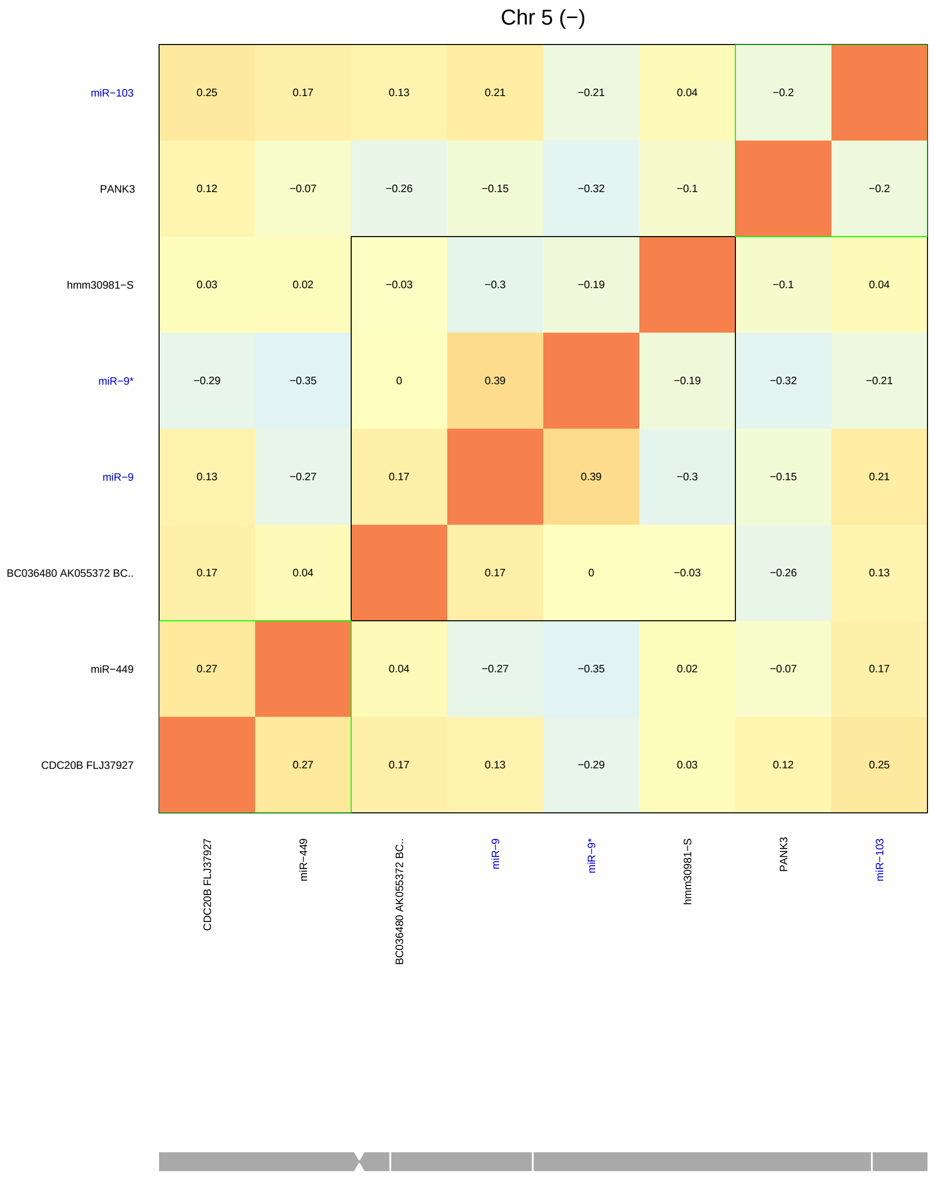
Chr 5 (−)
miR−103
PANK3
hmm30981−S
miR−9*
miR−9
BC036480 AK055372 BC..
miR−449
CDC20B FLJ37927

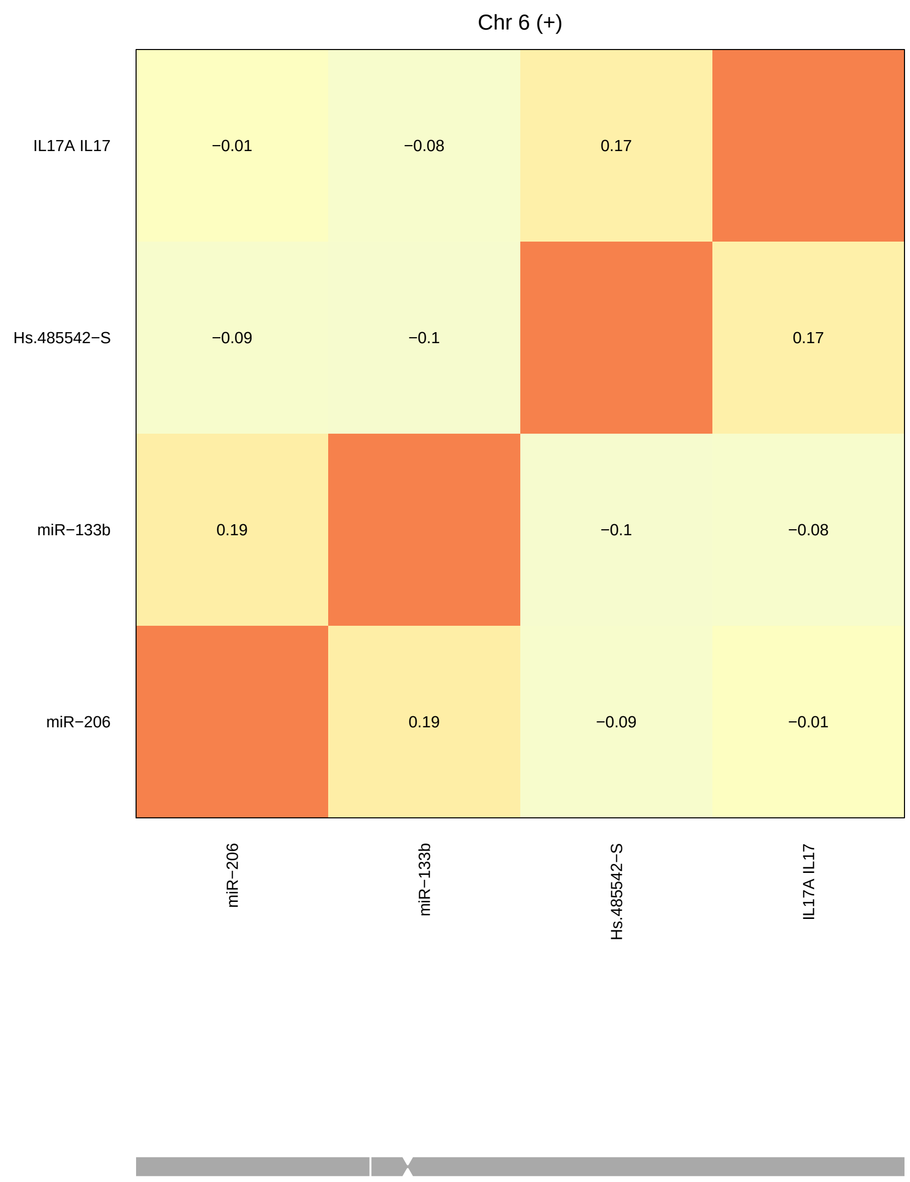
Chr 6 (+)
IL17A IL17
Hs.485542−S
miR−133b
miR−206
−0.01
−0.08
0.17
−0.09
−0.1
0.17
0.19
−0.1
−0.08
0.19
−0.09
−0.01
miR−206
miR−133b
Hs.485542−S
IL17A IL17

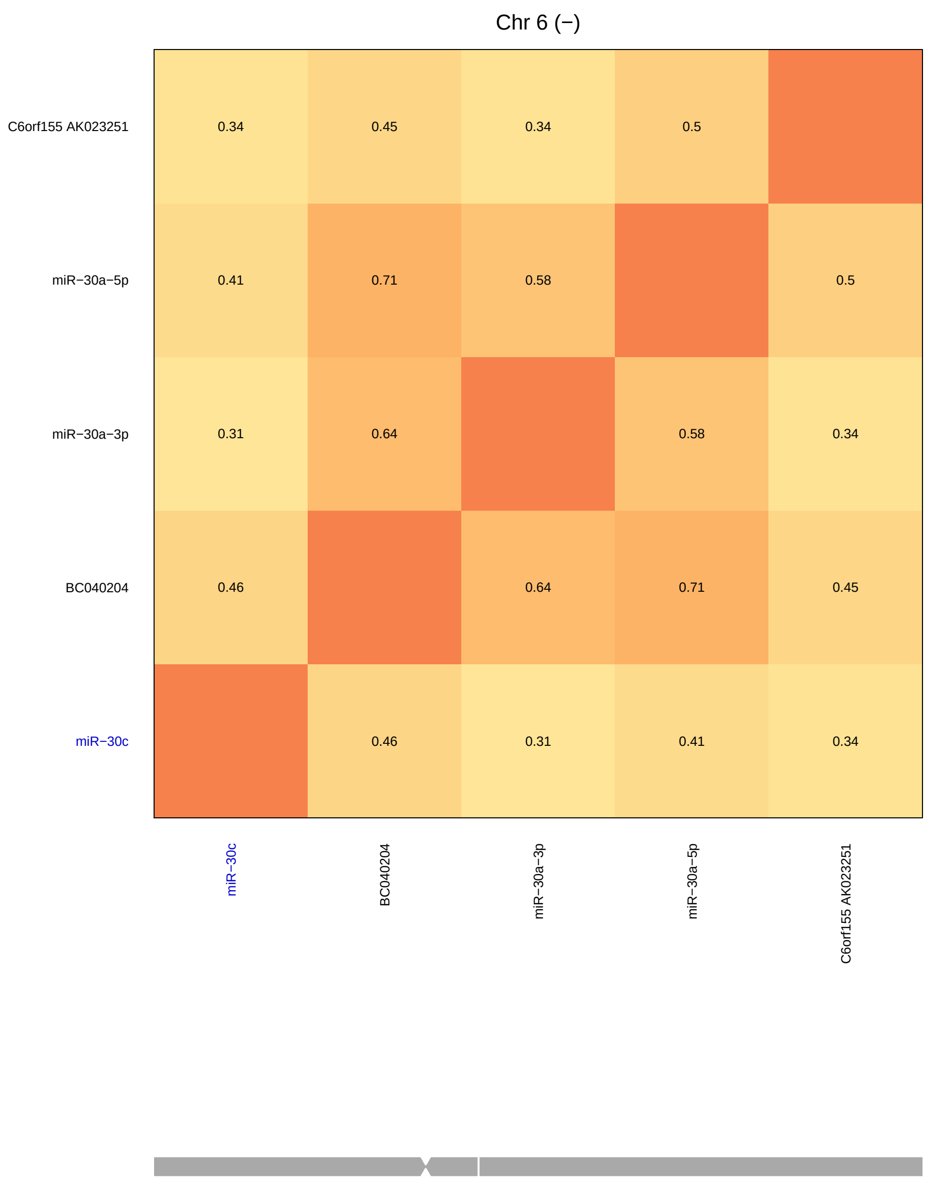
Chr 6 (−)
C6orf155 AK023251
miR−30a−5p
miR−30a−3p
BC040204
miR−30c
0.34
0.45
0.34
0.5
0.41
0.71
0.58
0.5
0.31
0.64
0.58
0.34
0.46
0.64
0.71
0.45
0.46
0.31
0.41
0.34
miR−30c
BC040204
miR−30a−3p
miR−30a−5p
C6orf155 AK023251

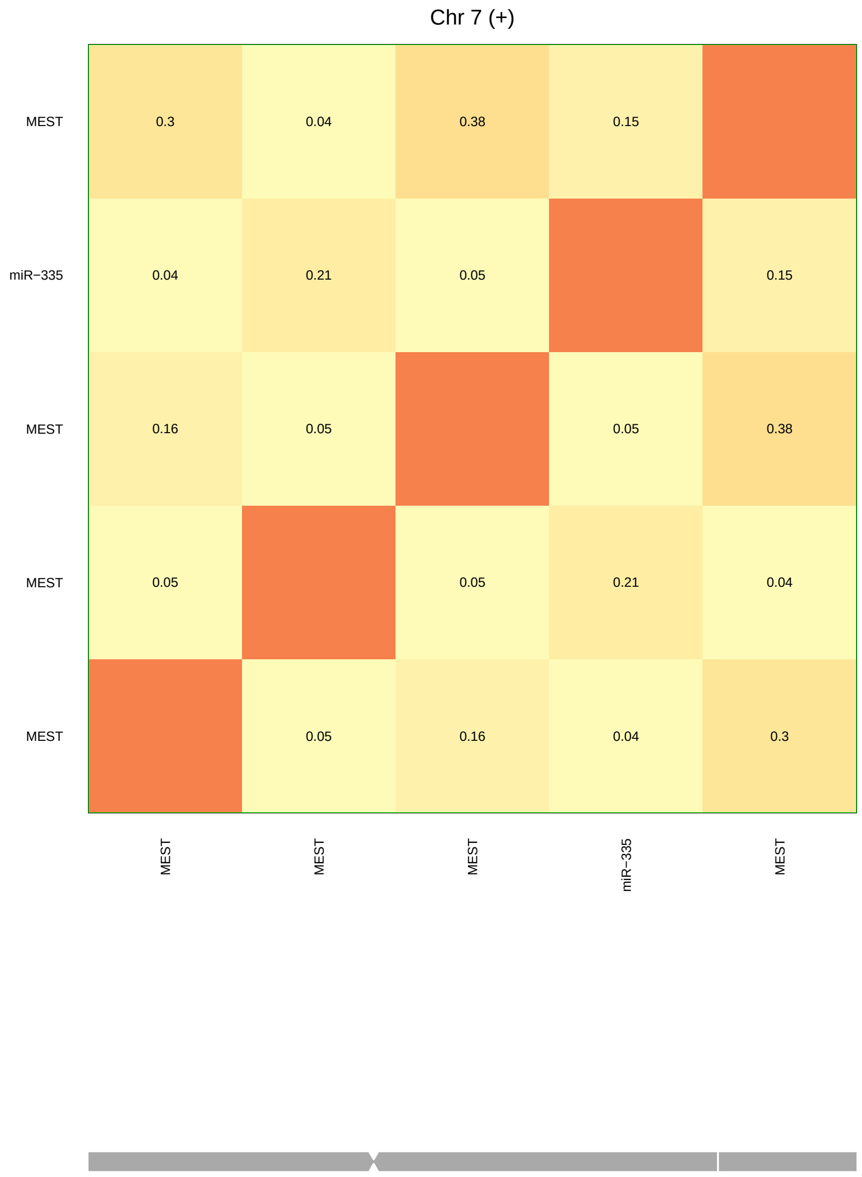
Chr 7 (+)
MEST
miR−335
MEST
MEST
MEST
0.3 0.04 0.38 0.15
0.04 0.21 0.05 0.15
0.16 0.05 0.05 0.38
0.05 0.05 0.21 0.04
0.05 0.16 0.04 0.3
MEST
MEST
MEST
miR−335
MEST

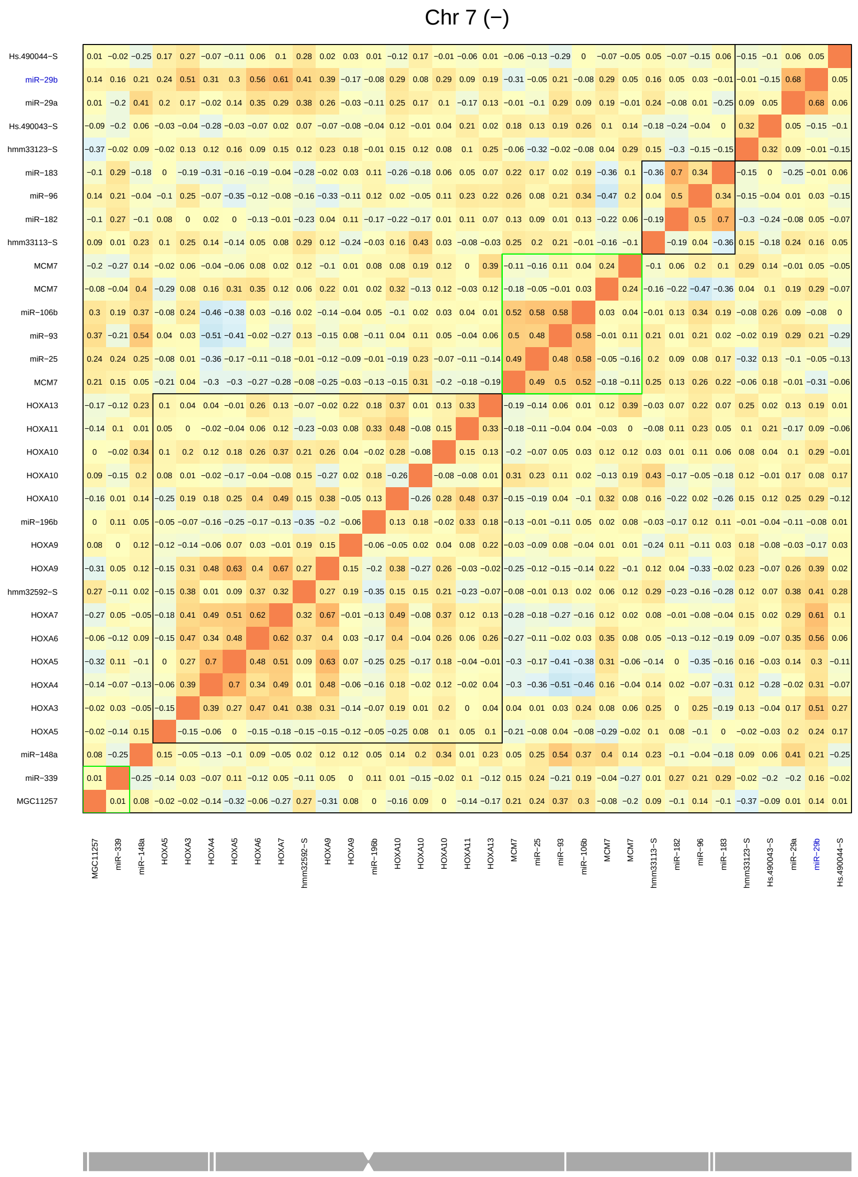
Chr 7 (−)
Hs.490044−S
miR−29b
miR−29a
Hs.490043−S
hmm33123−S
miR−183
miR−96
miR−182
hmm33113−S
MCM7
MCM7
miR−106b
miR−93
miR−25
MCM7
HOXA13
HOXA11
HOXA10
HOXA10
HOXA10
miR−196b
HOXA9
HOXA9
hmm32592−S
HOXA7
HOXA6
HOXA5
HOXA4
HOXA3
HOXA5
miR−148a
miR−339
MGC11257

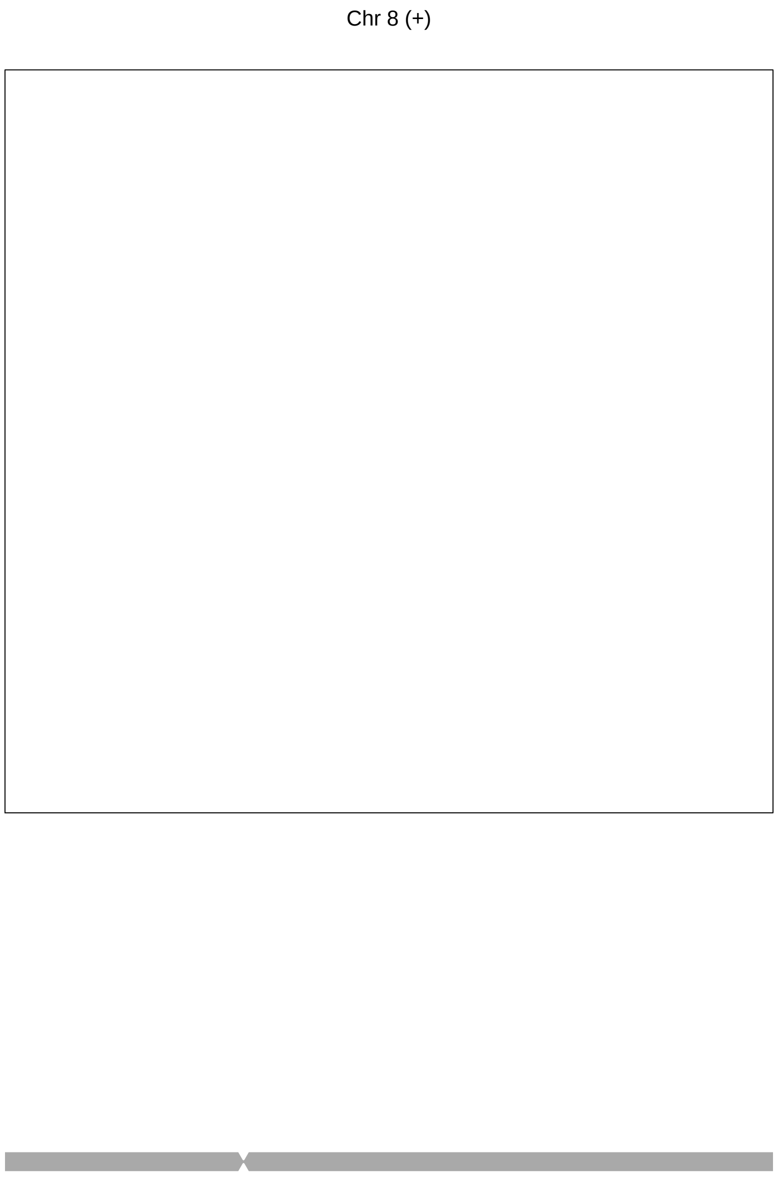
Chr 8 (+)

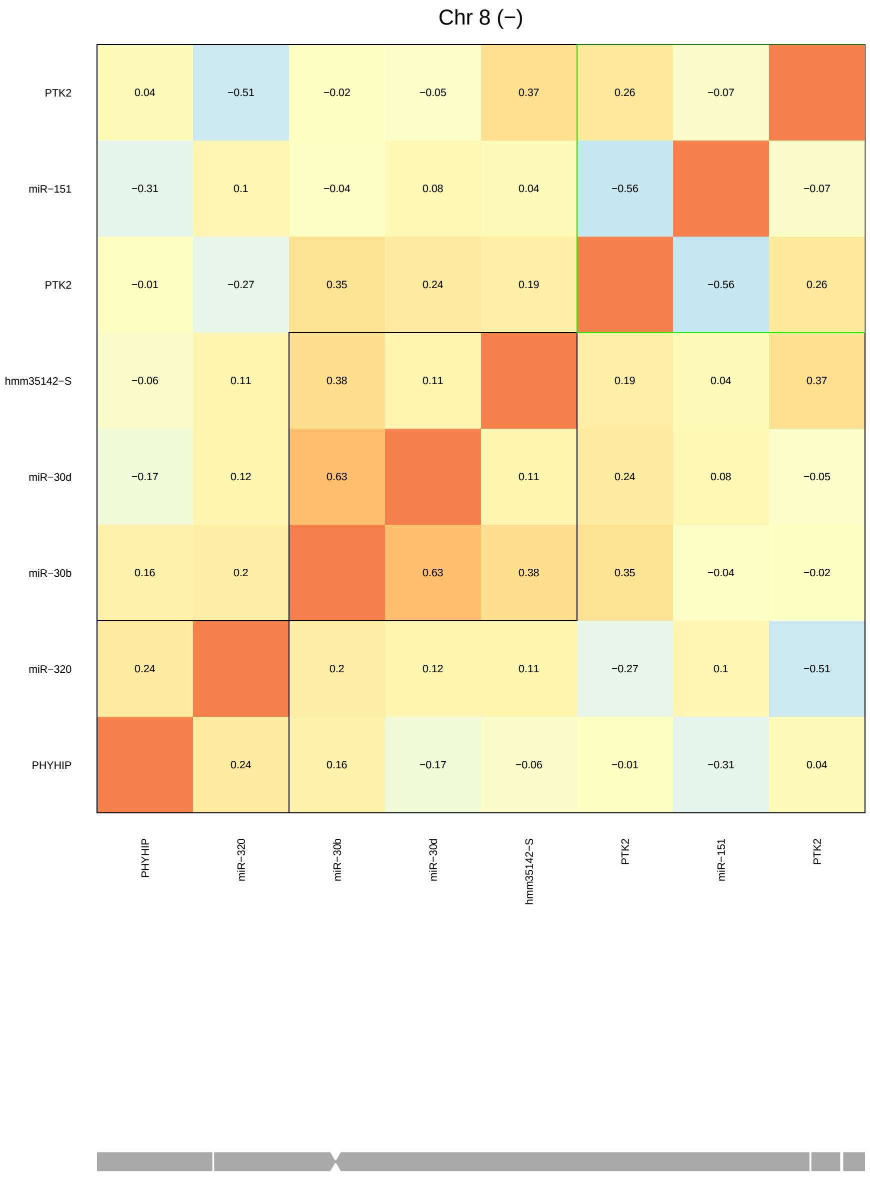
Chr 8 (−)
PTK2
miR−151
PTK2
hmm35142−S
miR−30d
miR−30b
miR−320
PHYHIP
0.04 −0.51 −0.02 −0.05 0.37 0.26 −0.07
−0.31 0.1 −0.04 0.08 0.04 −0.56 −0.07
−0.01 −0.27 0.35 0.24 0.19 −0.56 0.26
−0.06 0.11 0.38 0.11 0.19 0.04 0.37
−0.17 0.12 0.63 0.11 0.24 0.08 −0.05
0.16 0.2 0.63 0.38 0.35 −0.04 −0.02
0.24 0.2 0.12 0.11 −0.27 0.1 −0.51
0.24 0.16 −0.17 −0.06 −0.01 −0.31 0.04
PHYHIP
miR−320
miR−30b
miR−30d
hmm35142−S
PTK2
miR−151
PTK2

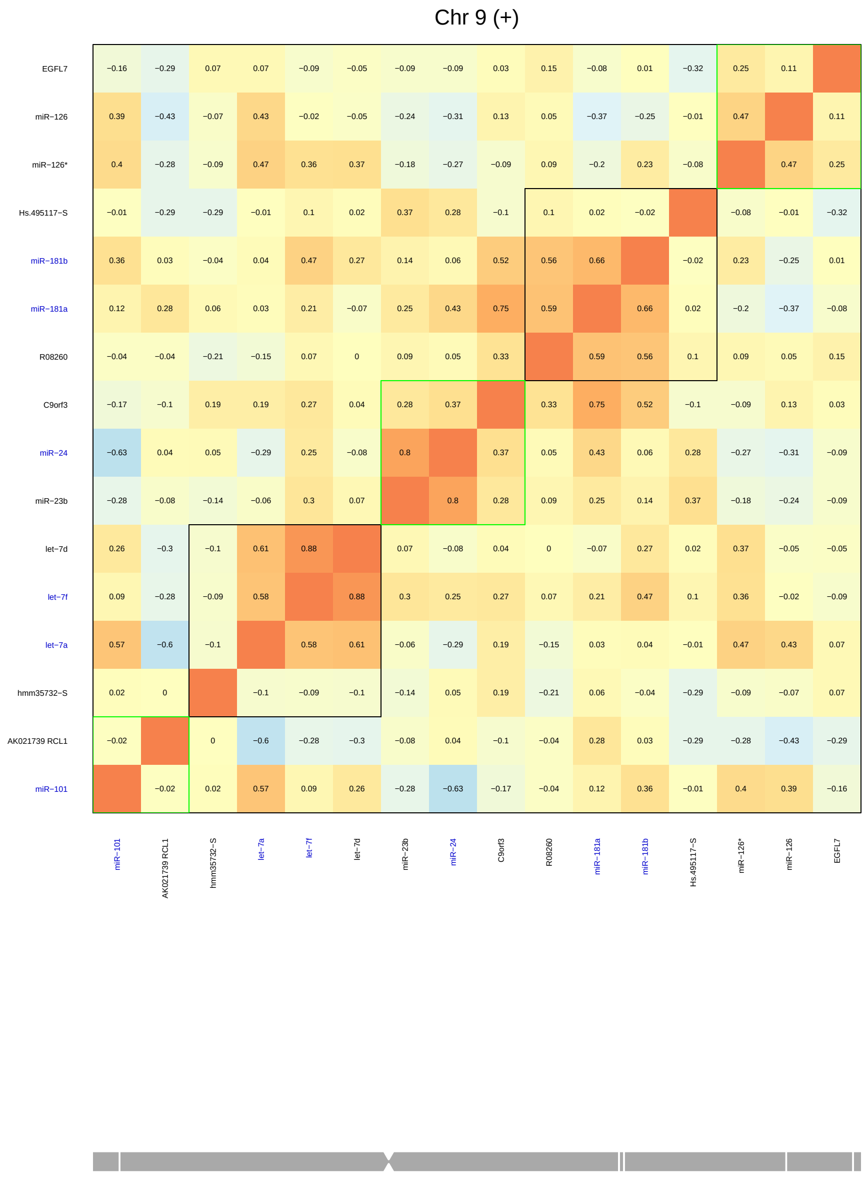
Chr 9 (+)
EGFL7
miR−126
miR−126*
Hs.495117−S
miR−181b
miR−181a
R08260
C9orf3
miR−24
miR−23b
let−7d
let−7f
let−7a
hmm35732−S
AK021739 RCL1
miR−101

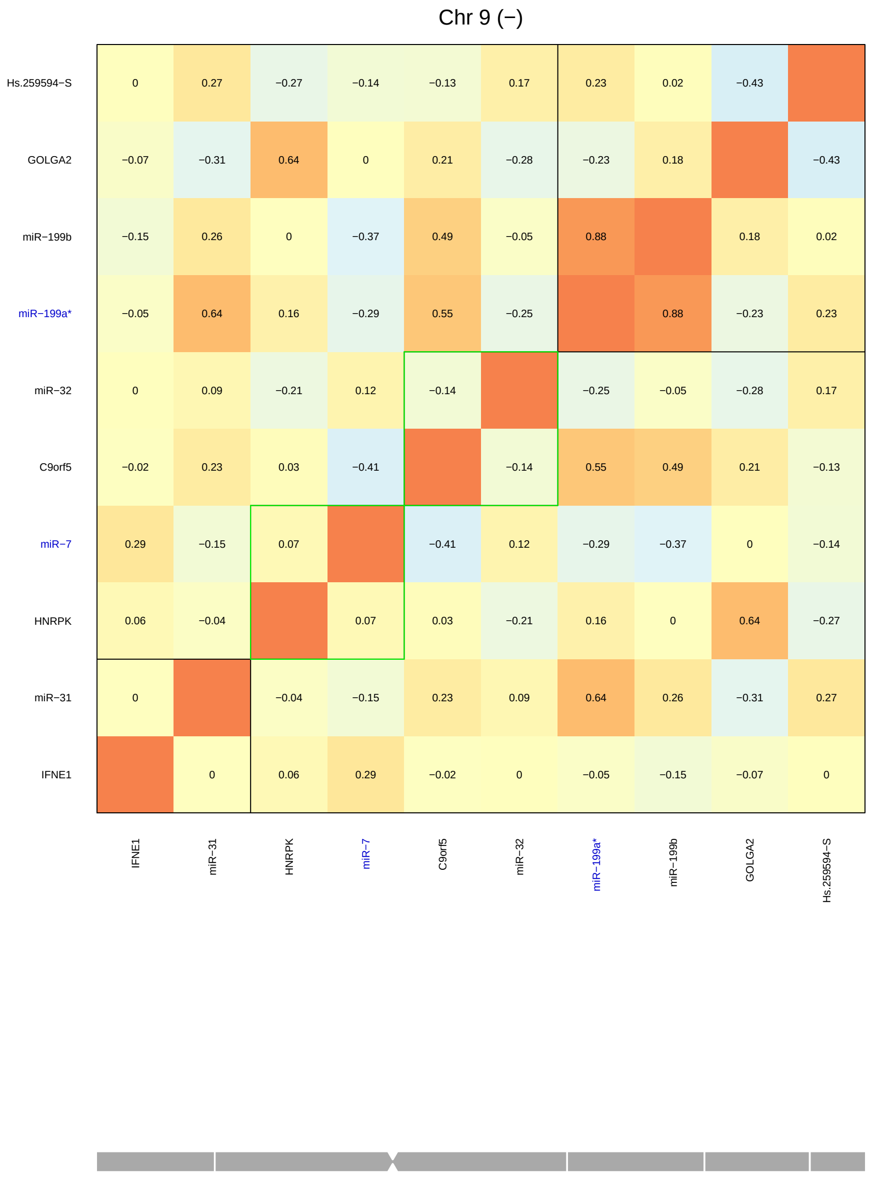
Chr 9 (−)
Hs.259594−S
GOLGA2
miR−199b
miR−199a*
miR−32
C9orf5
miR−7
HNRPK
miR−31
IFNE1

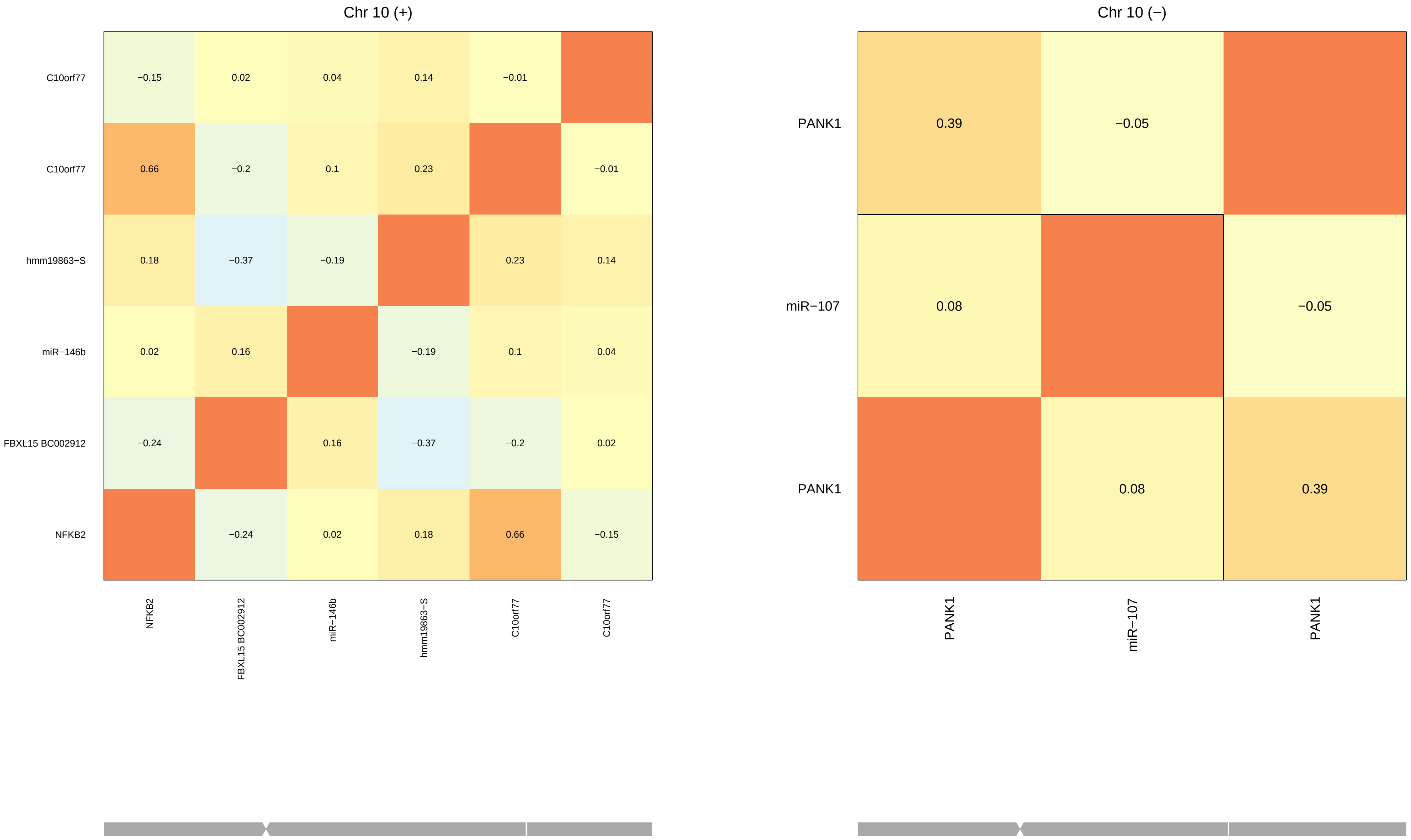
Chr 10 (+)
C10orf77
−0.15
0.02
0.04
0.14
−0.01
C10orf77
0.66
−0.2
0.1
0.23
−0.01
hmm19863−S
0.18
−0.37
−0.19
0.23
0.14
miR−146b
0.02
0.16
−0.19
0.1
0.04
FBXL15 BC002912
−0.24
0.16
−0.37
−0.2
0.02
NFKB2
−0.24
0.02
0.18
0.66
−0.15
NFKB2
FBXL15 BC002912
miR−146b
hmm19863−S
C10orf77
C10orf77
Chr 10 (−)
PANK1
0.39
−0.05
miR−107
0.08
−0.05
PANK1
0.08
0.39
PANK1
miR−107
PANK1

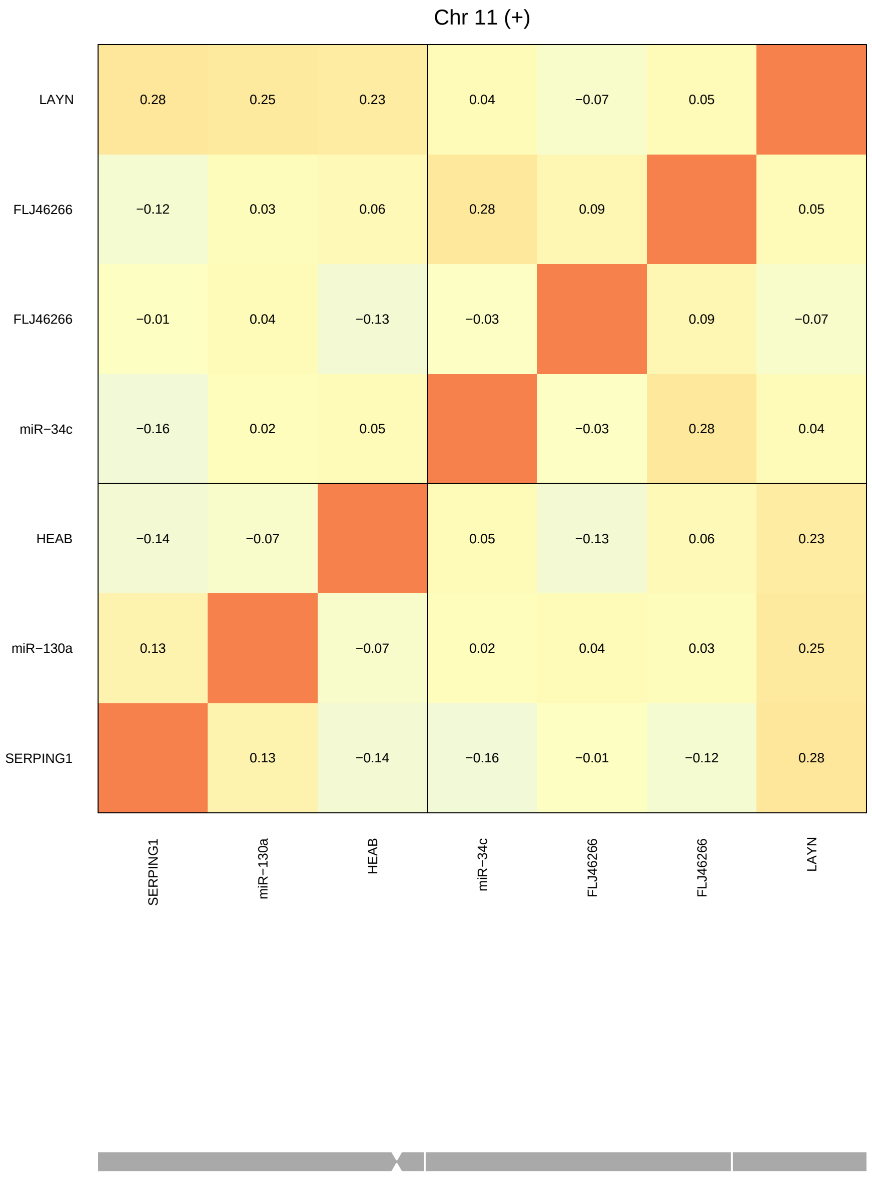
Chr 11 (+)
LAYN
FLJ46266
FLJ46266
miR−34c
HEAB
miR−130a
SERPING1

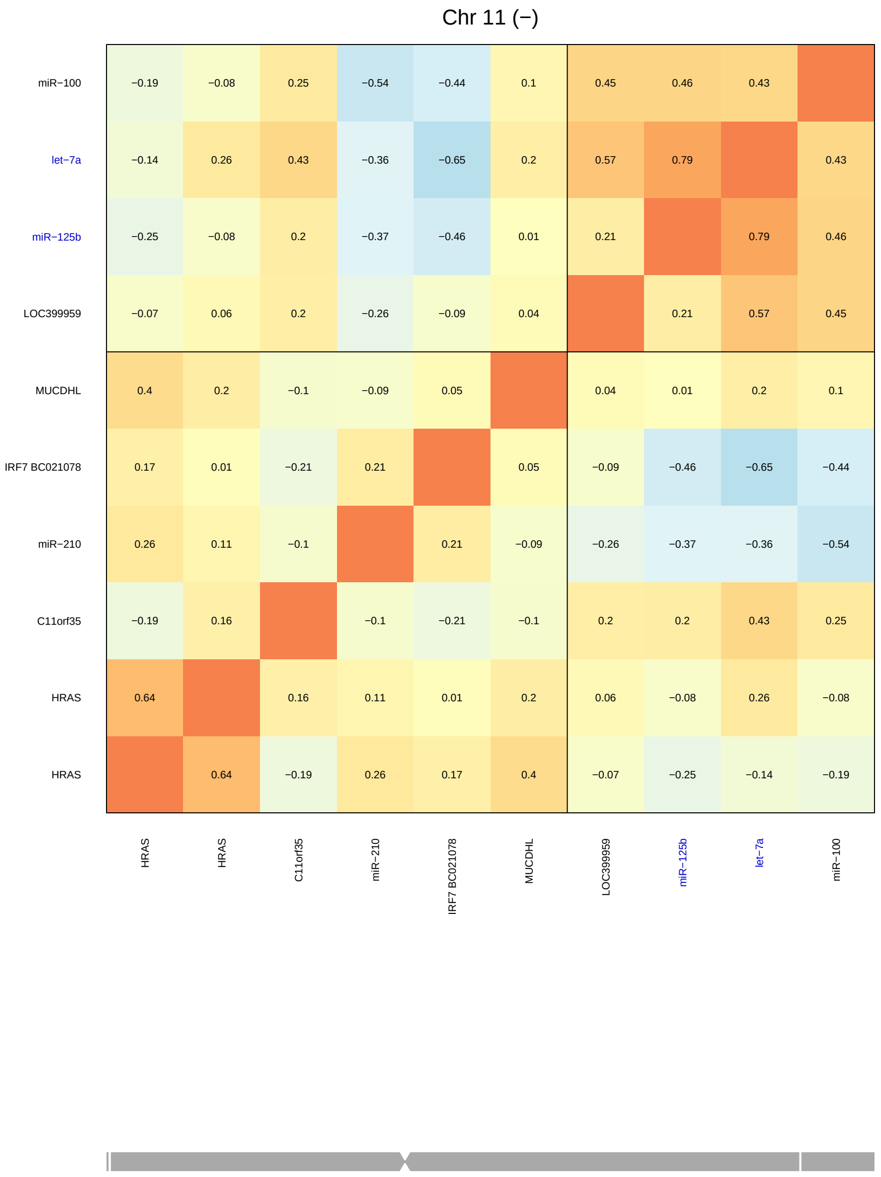
Chr 11 (−)
miR−100
let−7a
miR−125b
LOC399959
MUCDHL
IRF7 BC021078
miR−210
C11orf35
HRAS
HRAS

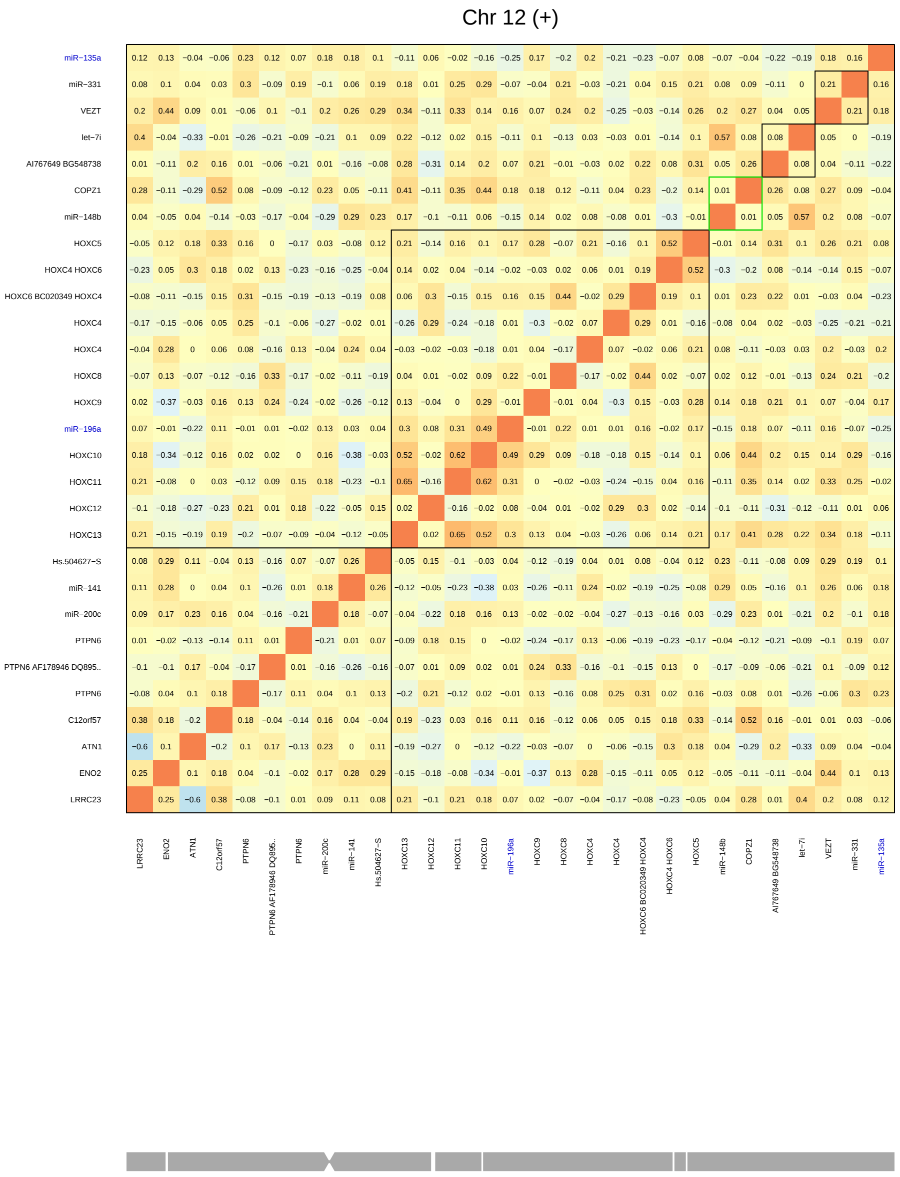
Chr 12 (+)
miR−135a
miR−331
VEZT
let−7i
AI767649 BG548738
COPZ1
miR−148b
HOXC5
HOXC4 HOXC6
HOXC6 BC020349 HOXC4
HOXC4
HOXC4
HOXC8
HOXC9
miR−196a
HOXC10
HOXC11
HOXC12
HOXC13
Hs.504627−S
miR−141
miR−200c
PTPN6
PTPN6 AF178946 DQ895..
PTPN6
C12orf57
ATN1
ENO2
LRRC23

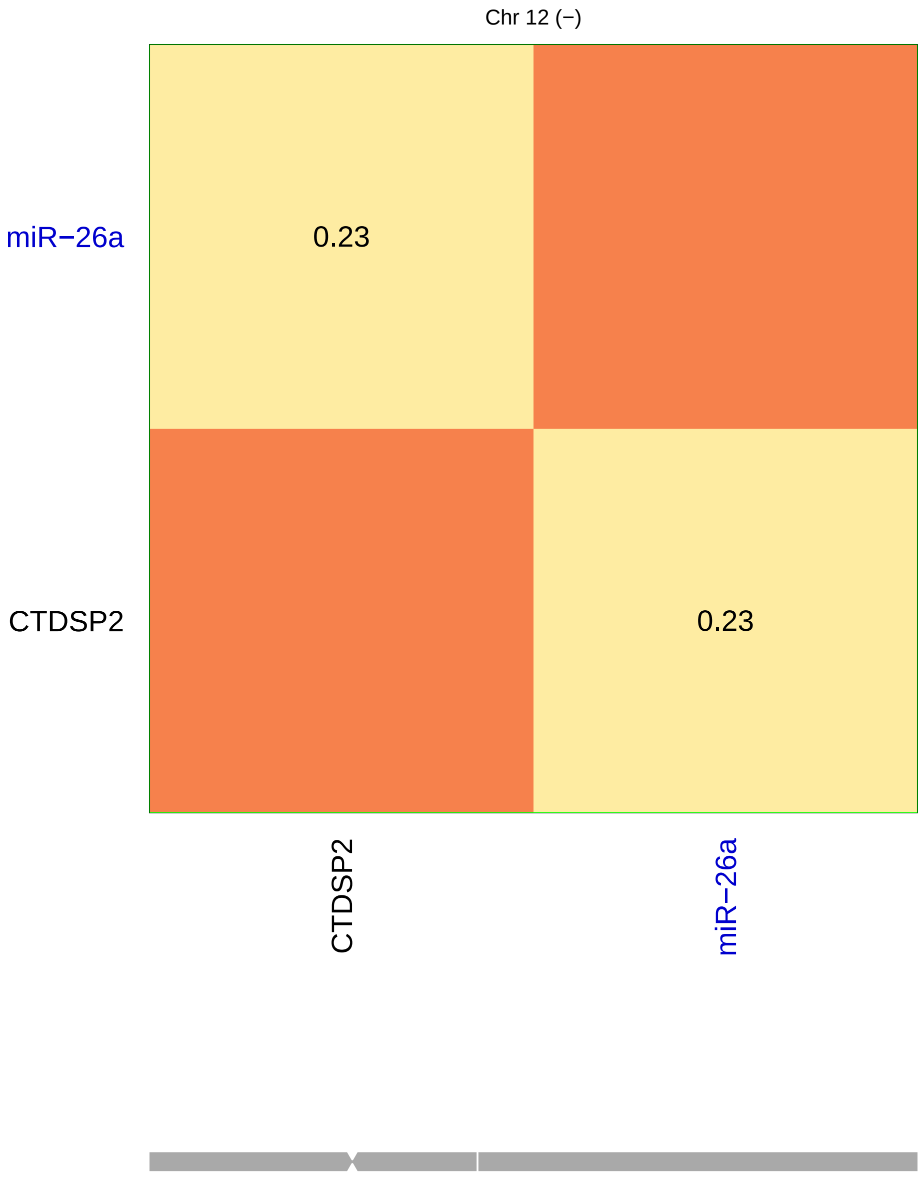
Chr 12 (−)
miR−26a
CTDSP2
0.23
0.23
CTDSP2
miR−26a

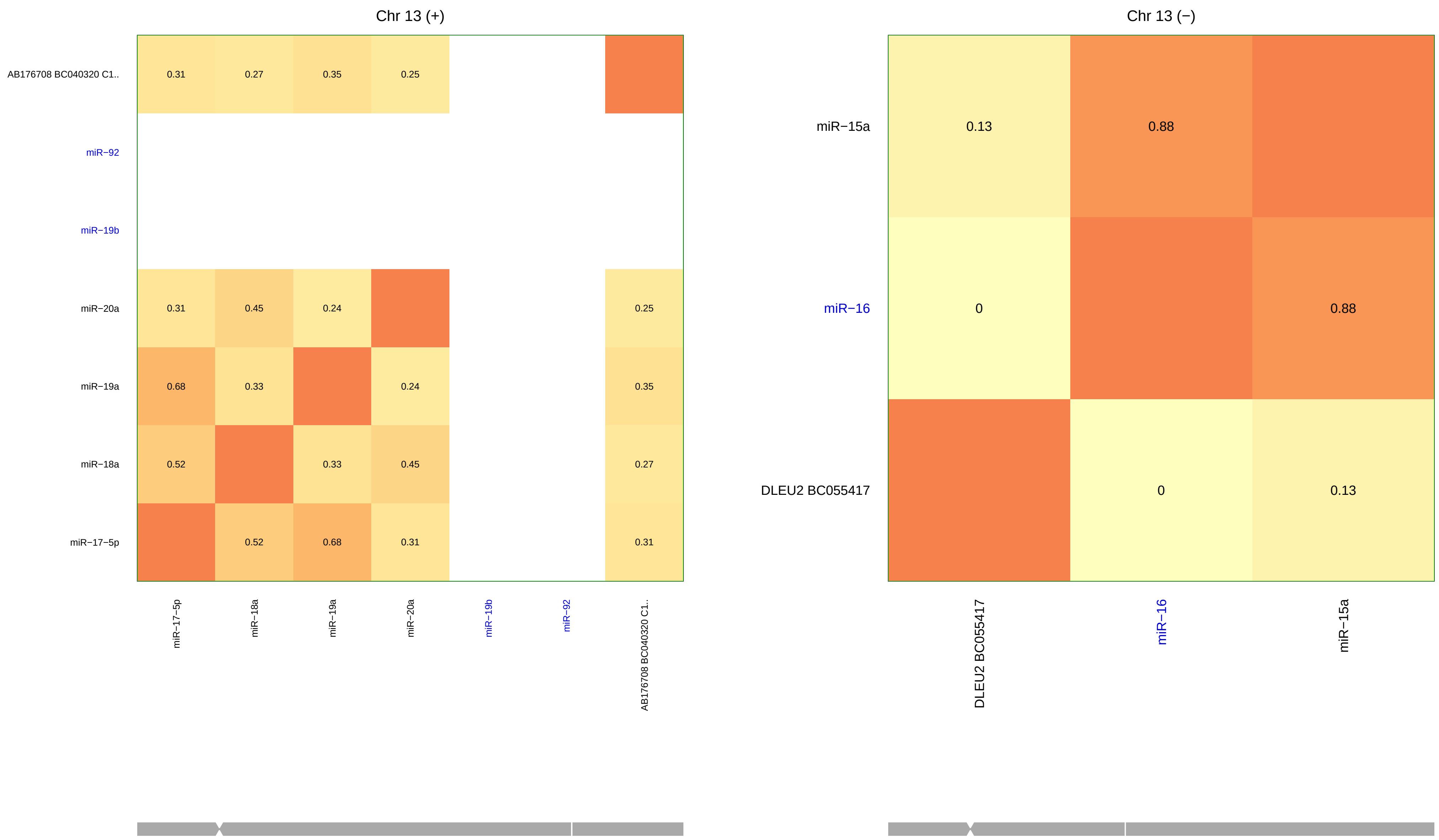

Chr 13 (+)
AB176708 BC040320 C1..
miR−92
miR−19b
miR−20a
miR−19a
miR−18a
miR−17−5p
0.31
0.27
0.35
0.25
0.31
0.45
0.24
0.25
0.68
0.33
0.24
0.35
0.52
0.33
0.45
0.27
0.52
0.68
0.31
0.31
miR−17−5p
miR−18a
miR−19a
miR−20a
miR−19b
miR−92
AB176708 BC040320 C1..
Chr 13 (−)
miR−15a
miR−16
DLEU2 BC055417
0.13
0.88
0
0.88
0
0.13
DLEU2 BC055417
miR−16
miR−15a

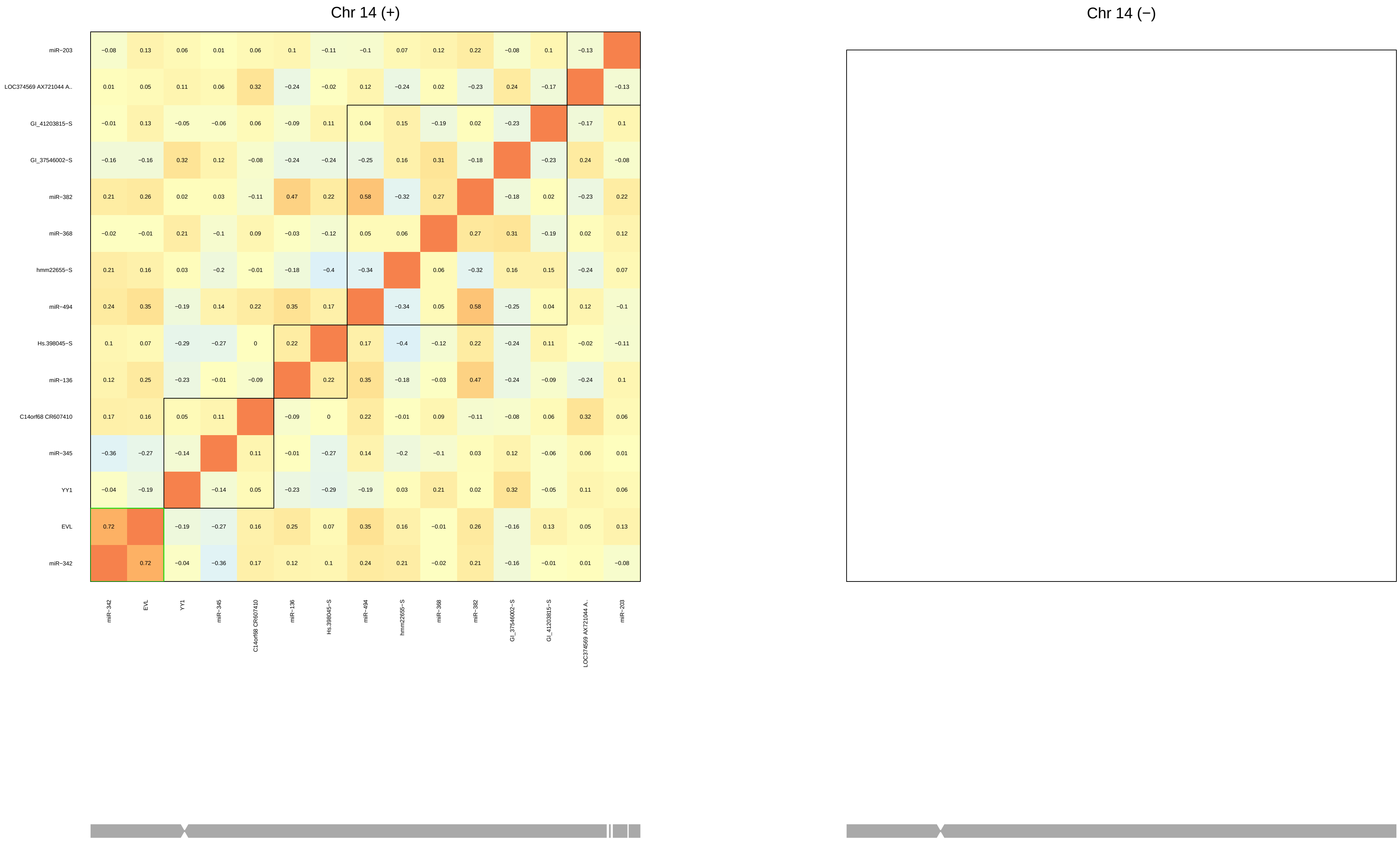
Chr 14 (+)
Chr 14 (−)
miR−203
LOC374569 AX721044 A..
GI_41203815−S
GI_37546002−S
miR−382
miR−368
hmm22655−S
miR−494
Hs.398045−S
miR−136
C14orf68 CR607410
miR−345
YY1
EVL
miR−342

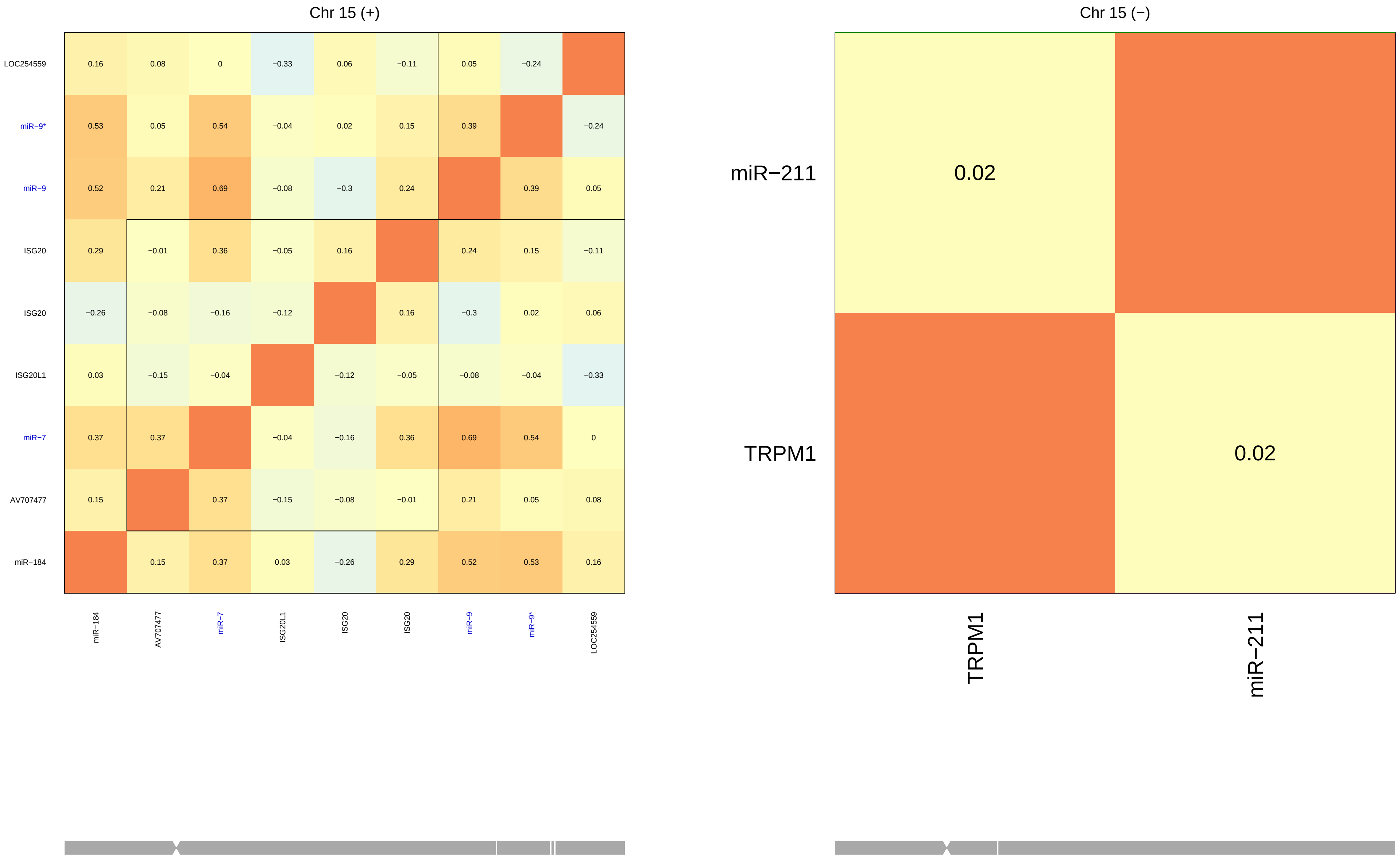
Chr 15 (+)
LOC254559 0.16 0.08 0 −0.33 0.06 −0.11 0.05 −0.24
miR−9* 0.53 0.05 0.54 −0.04 0.02 0.15 0.39 −0.24
miR−9 0.52 0.21 0.69 −0.08 −0.3 0.24 0.39 0.05
ISG20 0.29 −0.01 0.36 −0.05 0.16 0.24 0.15 −0.11
ISG20 −0.26 −0.08 −0.16 −0.12 0.16 −0.3 0.02 0.06
ISG20L1 0.03 −0.15 −0.04 −0.12 −0.05 −0.08 −0.04 −0.33
miR−7 0.37 0.37 −0.04 −0.16 0.36 0.69 0.54 0
AV707477 0.15 0.37 −0.15 −0.08 −0.01 0.21 0.05 0.08
miR−184 0.15 0.37 0.03 −0.26 0.29 0.52 0.53 0.16
miR−184
AV707477
miR−7
ISG20L1
ISG20
ISG20
miR−9
miR−9*
LOC254559
Chr 15 (−)
miR−211 0.02
TRPM1 0.02
TRPM1
miR−211

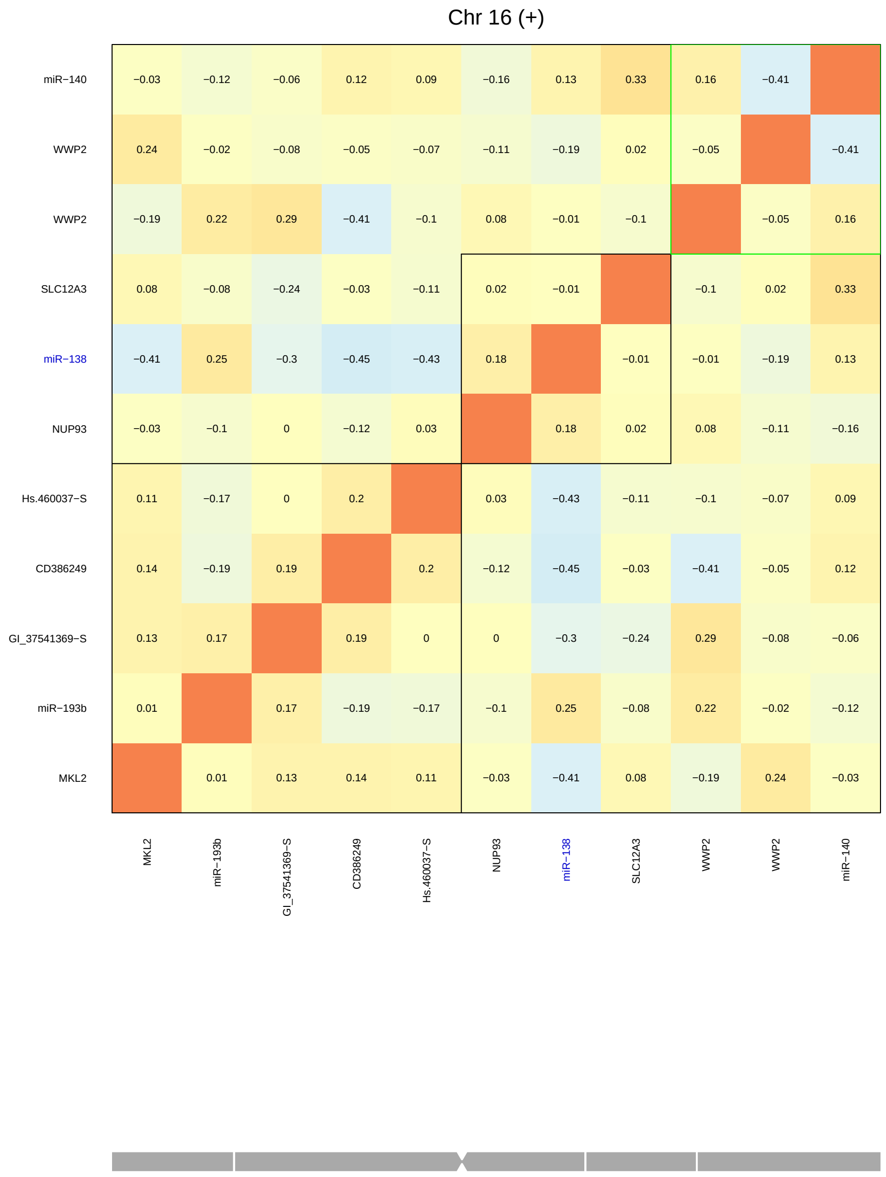
Chr 16 (+)
miR−140
WWP2
WWP2
SLC12A3
miR−138
NUP93
Hs.460037−S
CD386249
GI_37541369−S
miR−193b
MKL2

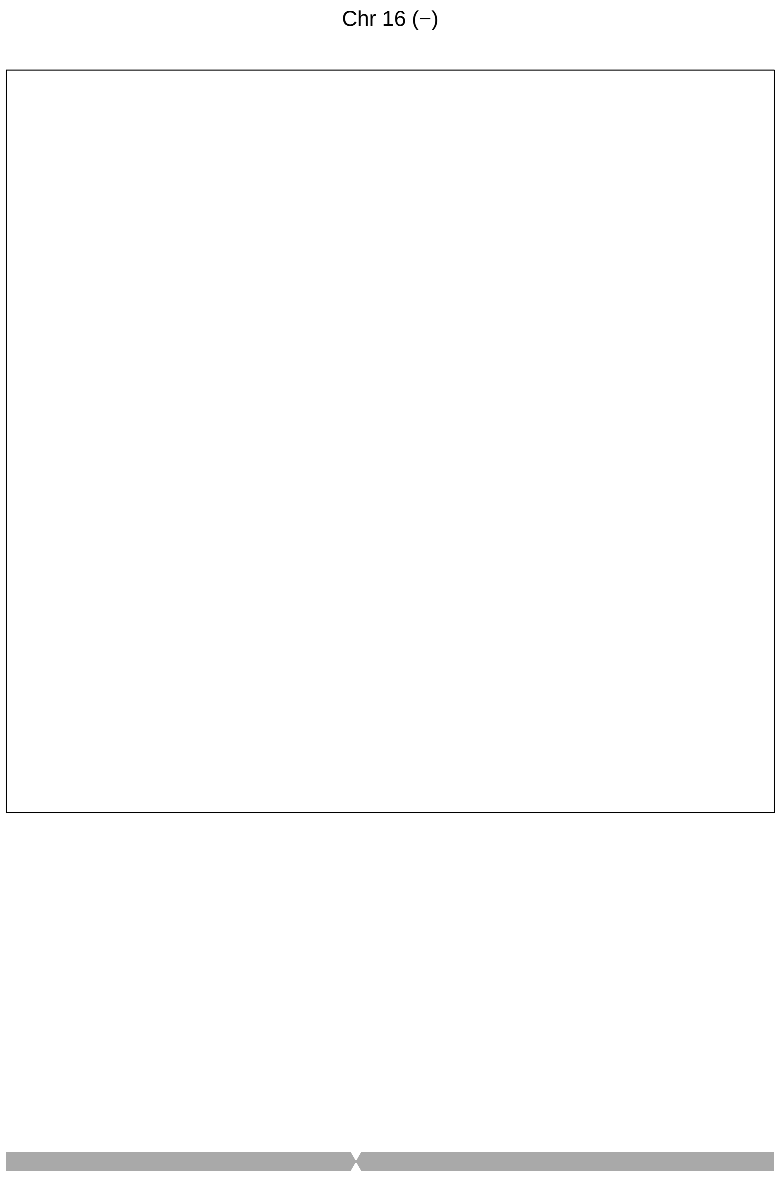
Chr 16 (−)

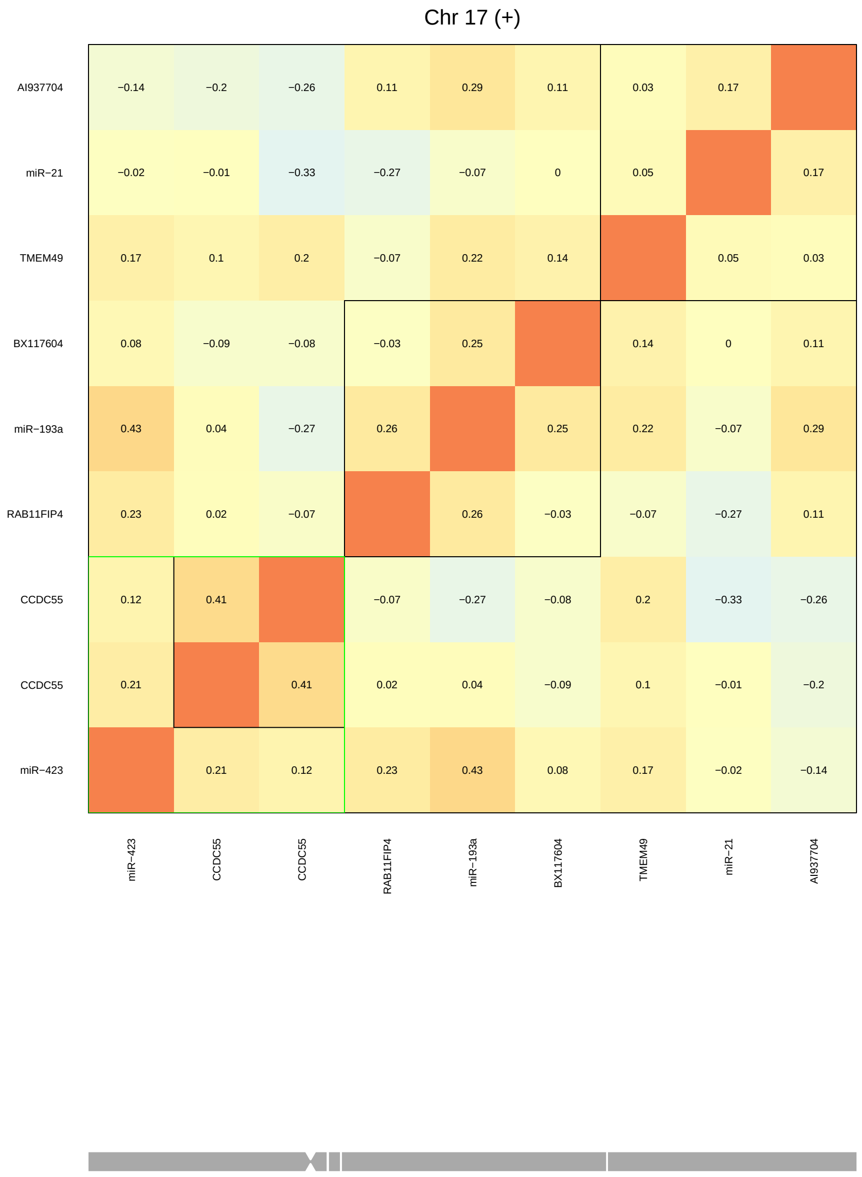
Chr 17 (+)
AI937704
miR−21
TMEM49
BX117604
miR−193a
RAB11FIP4
CCDC55
CCDC55
miR−423
−0.14 −0.2 −0.26 0.11 0.29 0.11 0.03 0.17
−0.02 −0.01 −0.33 −0.27 −0.07 0 0.05 0.17
0.17 0.1 0.2 −0.07 0.22 0.14 0.05 0.03
0.08 −0.09 −0.08 −0.03 0.25 0.14 0 0.11
0.43 0.04 −0.27 0.26 0.25 0.22 −0.07 0.29
0.23 0.02 −0.07 0.26 −0.03 −0.07 −0.27 0.11
0.12 0.41 −0.07 −0.27 −0.08 0.2 −0.33 −0.26
0.21 0.41 0.02 0.04 −0.09 0.1 −0.01 −0.2
0.21 0.12 0.23 0.43 0.08 0.17 −0.02 −0.14
miR−423
CCDC55
CCDC55
RAB11FIP4
miR−193a
BX117604
TMEM49
miR−21
AI937704

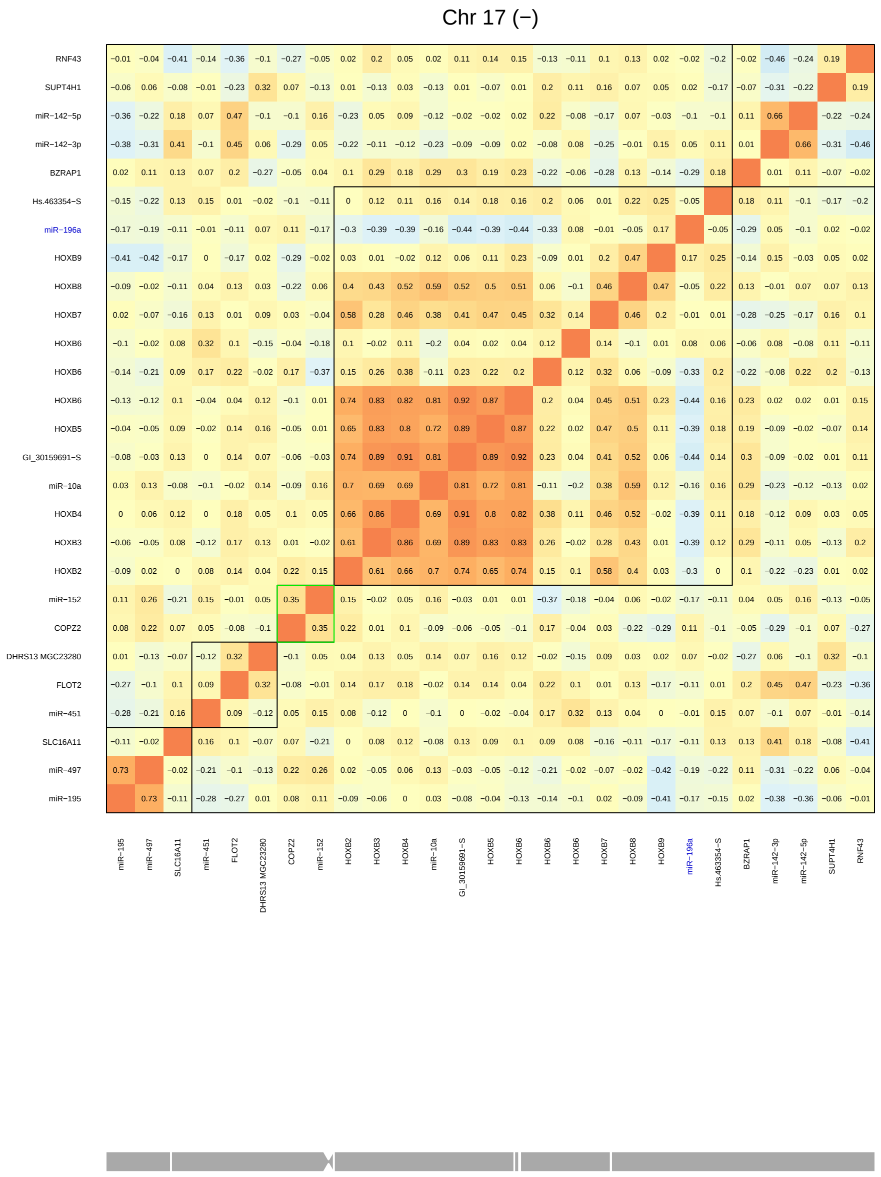
Chr 17 (−)
RNF43
SUPT4H1
miR−142−5p
miR−142−3p
BZRAP1
Hs.463354−S
miR−196a
HOXB9
HOXB8
HOXB7
HOXB6
HOXB6
HOXB6
HOXB5
GI_30159691−S
miR−10a
HOXB4
HOXB3
HOXB2
miR−152
COPZ2
DHRS13 MGC23280
FLOT2
miR−451
SLC16A11
miR−497
miR−195

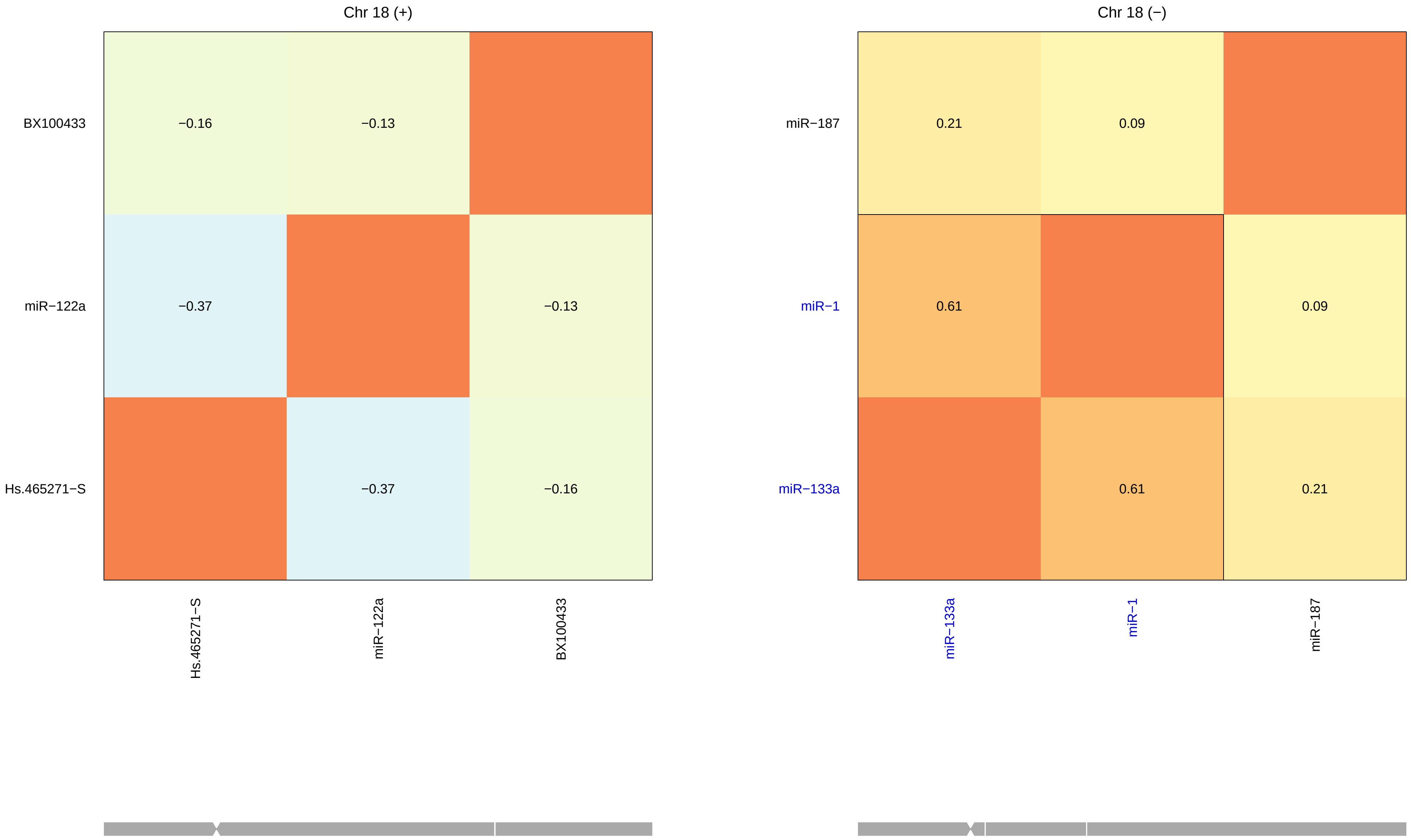

Chr 18 (+)
BX100433
miR−122a
Hs.465271−S
−0.16
−0.13
−0.37
−0.13
−0.37
−0.16
Hs.465271−S
miR−122a
BX100433
Chr 18 (−)
miR−187
miR−1
miR−133a
0.21
0.09
0.61
0.09
0.61
0.21
miR−133a
miR−1
miR−187

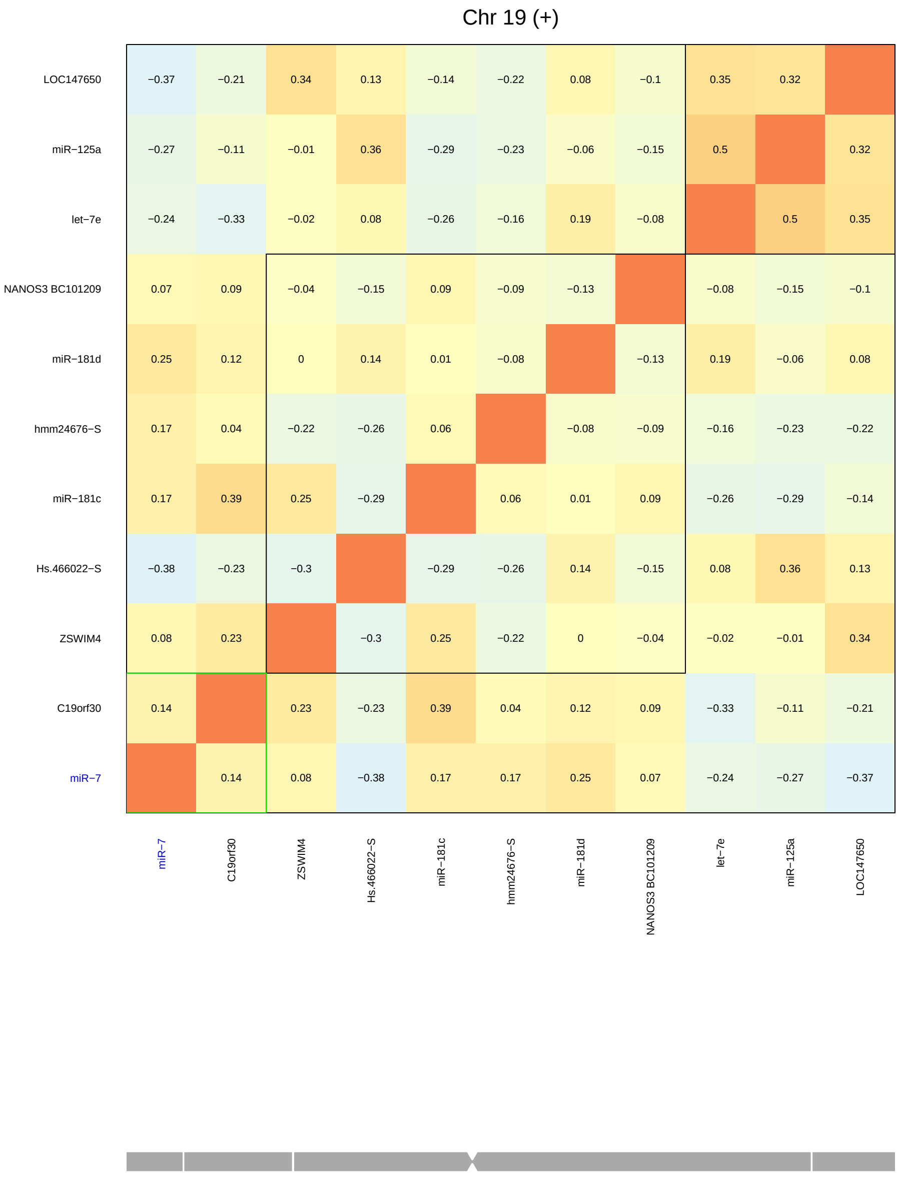

Chr 19 (+)
LOC147650
miR−125a
let−7e
NANOS3 BC101209
miR−181d
hmm24676−S
miR−181c
Hs.466022−S
ZSWIM4
C19orf30
miR−7


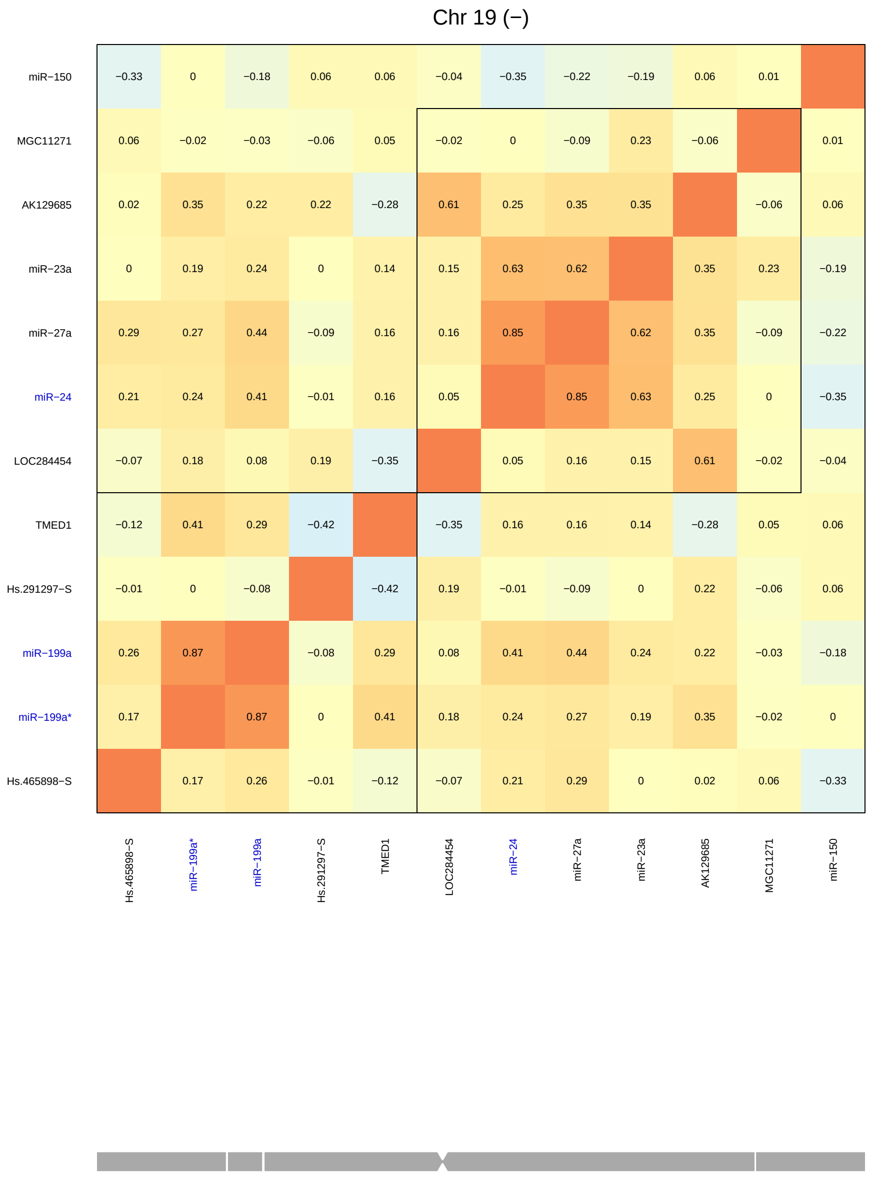

Chr 19 (−)
miR−150
MGC11271
AK129685
miR−23a
miR−27a
miR−24
LOC284454
TMED1
Hs.291297−S
miR−199a
miR−199a*
Hs.465898−S

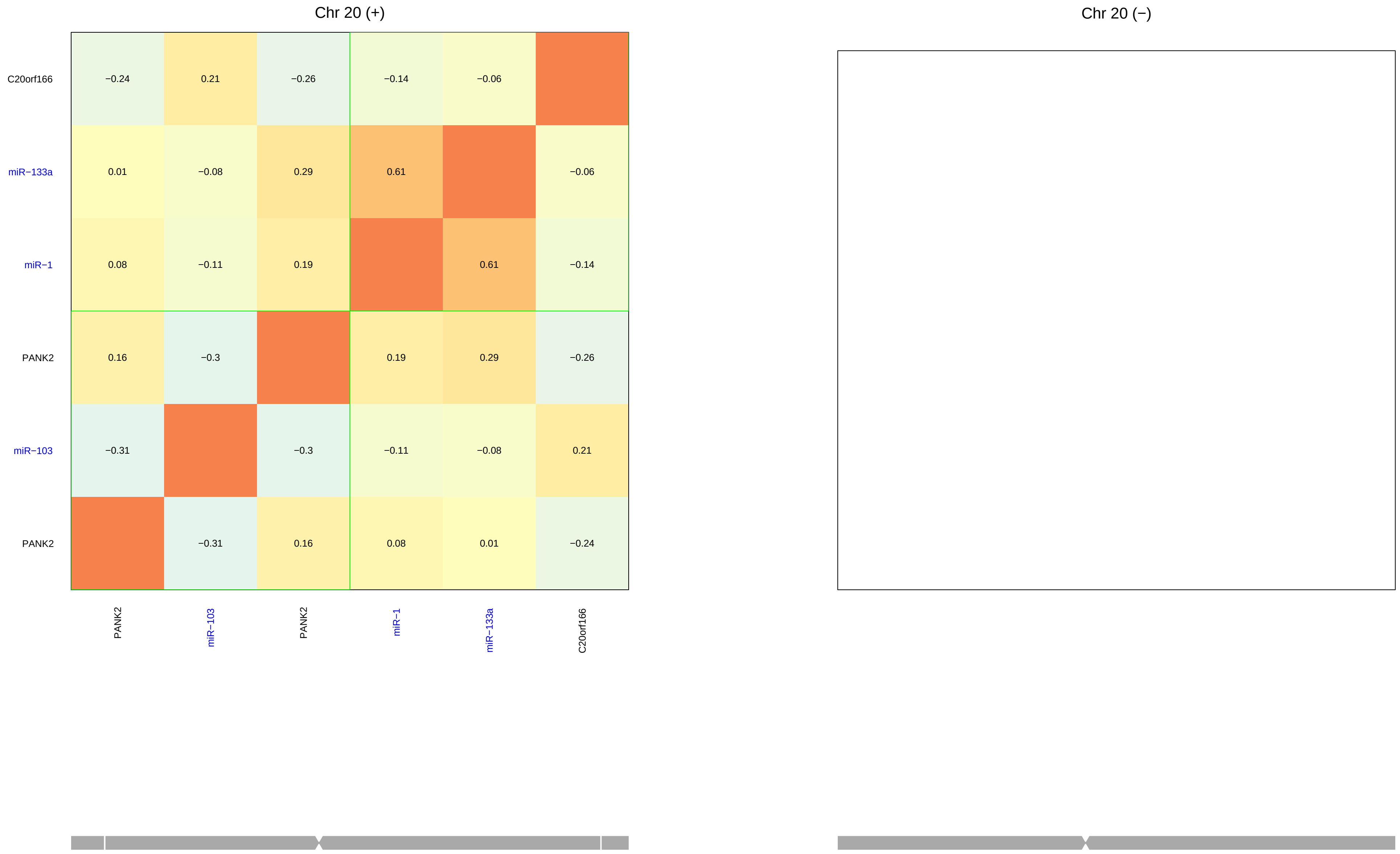
Chr 20 (+)
Chr 20 (−)
C20orf166
miR−133a
miR−1
PANK2
miR−103
PANK2
−0.24
0.21
−0.26
−0.14
−0.06
0.01
−0.08
0.29
0.61
−0.06
0.08
−0.11
0.19
0.61
−0.14
0.16
−0.3
0.19
0.29
−0.26
−0.31
−0.3
−0.11
−0.08
0.21
−0.31
0.16
0.08
0.01
−0.24
PANK2
miR−103
PANK2
miR−1
miR−133a
C20orf166

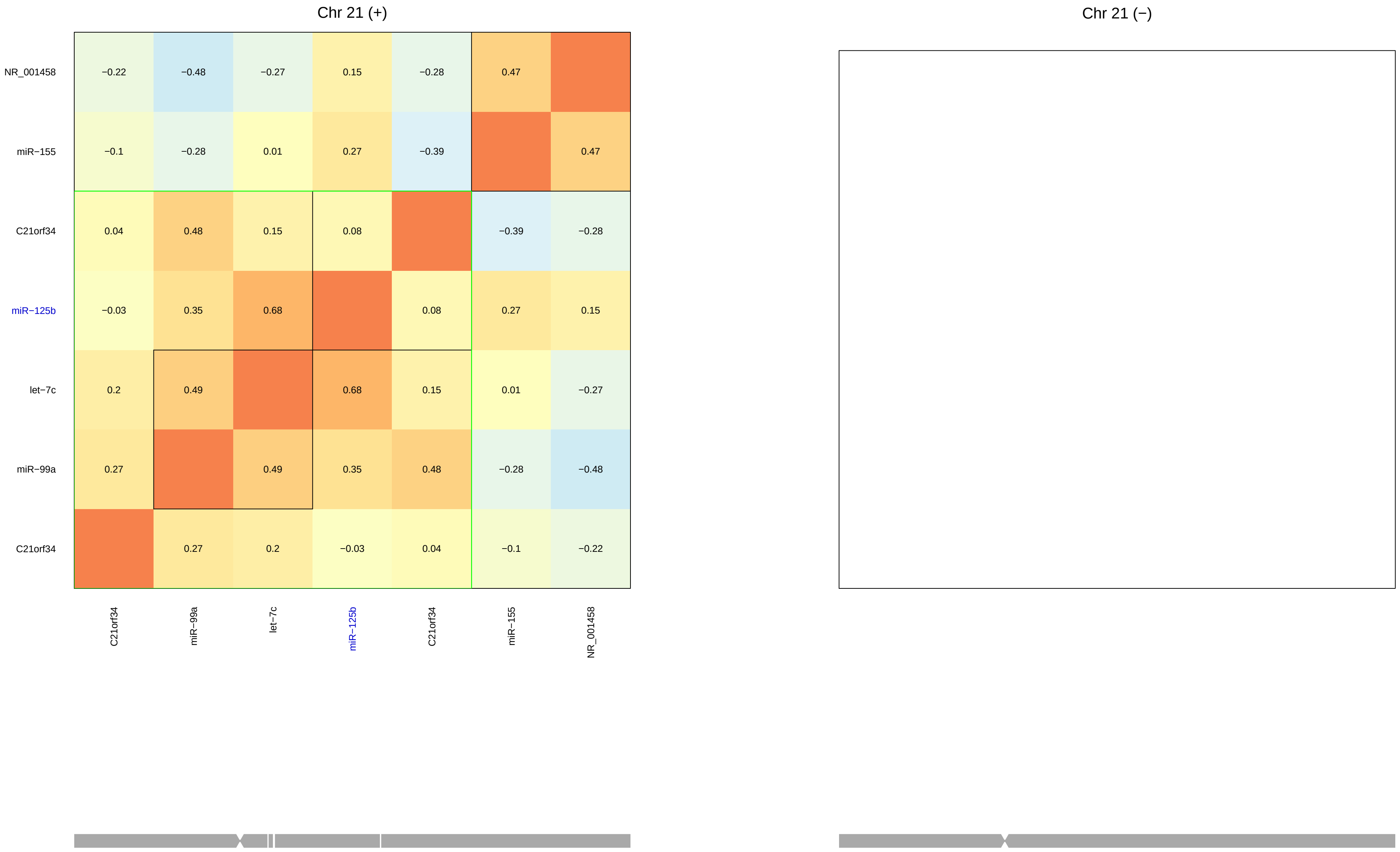
Chr 21 (+)
Chr 21 (−)
NR_001458
miR−155
C21orf34
miR−125b
let−7c
miR−99a
C21orf34
−0.22 −0.48 −0.27 0.15 −0.28 0.47
−0.1 −0.28 0.01 0.27 −0.39 0.47
0.04 0.48 0.15 0.08 −0.39 −0.28
−0.03 0.35 0.68 0.08 0.27 0.15
0.2 0.49 0.68 0.15 0.01 −0.27
0.27 0.49 0.35 0.48 −0.28 −0.48
0.27 0.2 −0.03 0.04 −0.1 −0.22
C21orf34
miR−99a
let−7c
miR−125b
C21orf34
miR−155
NR_001458

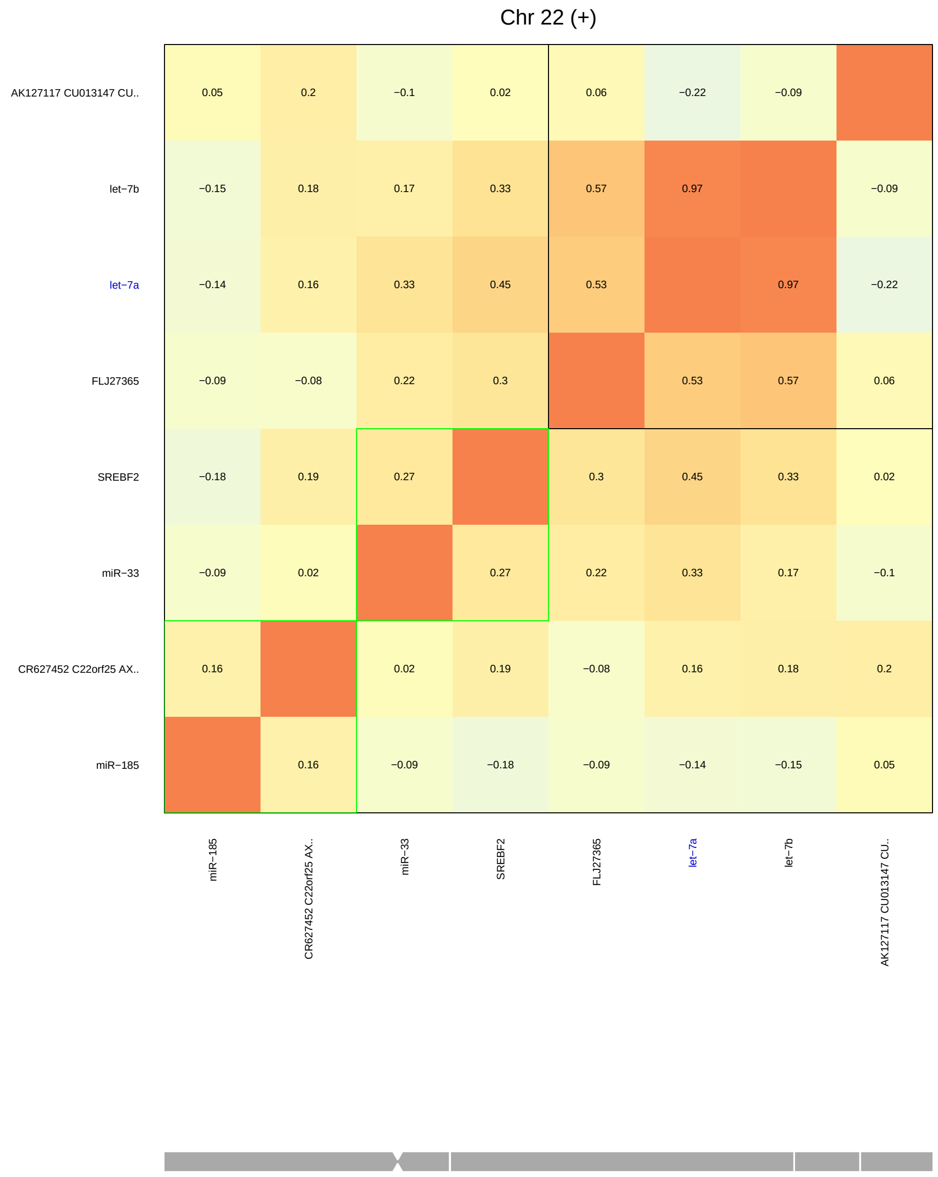
Chr 22 (+)
AK127117 CU013147 CU..
let−7b
let−7a
FLJ27365
SREBF2
miR−33
CR627452 C22orf25 AX..
miR−185

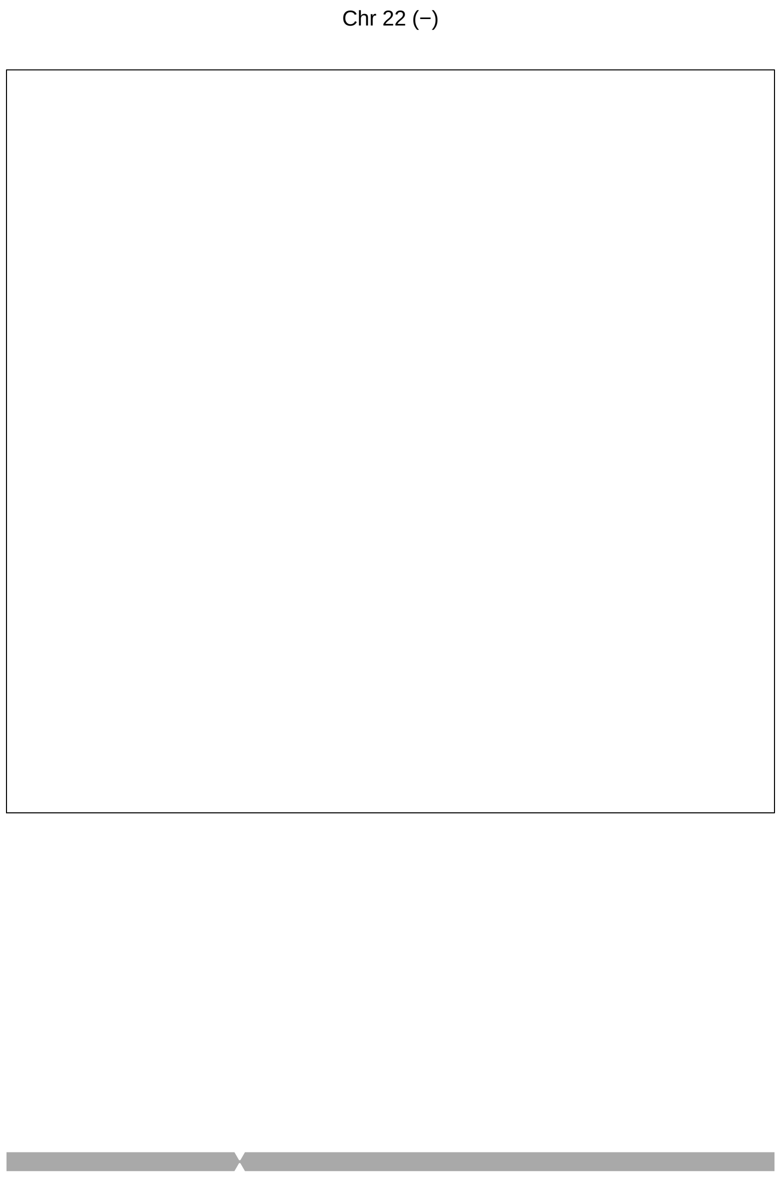
Chr 22 (−)

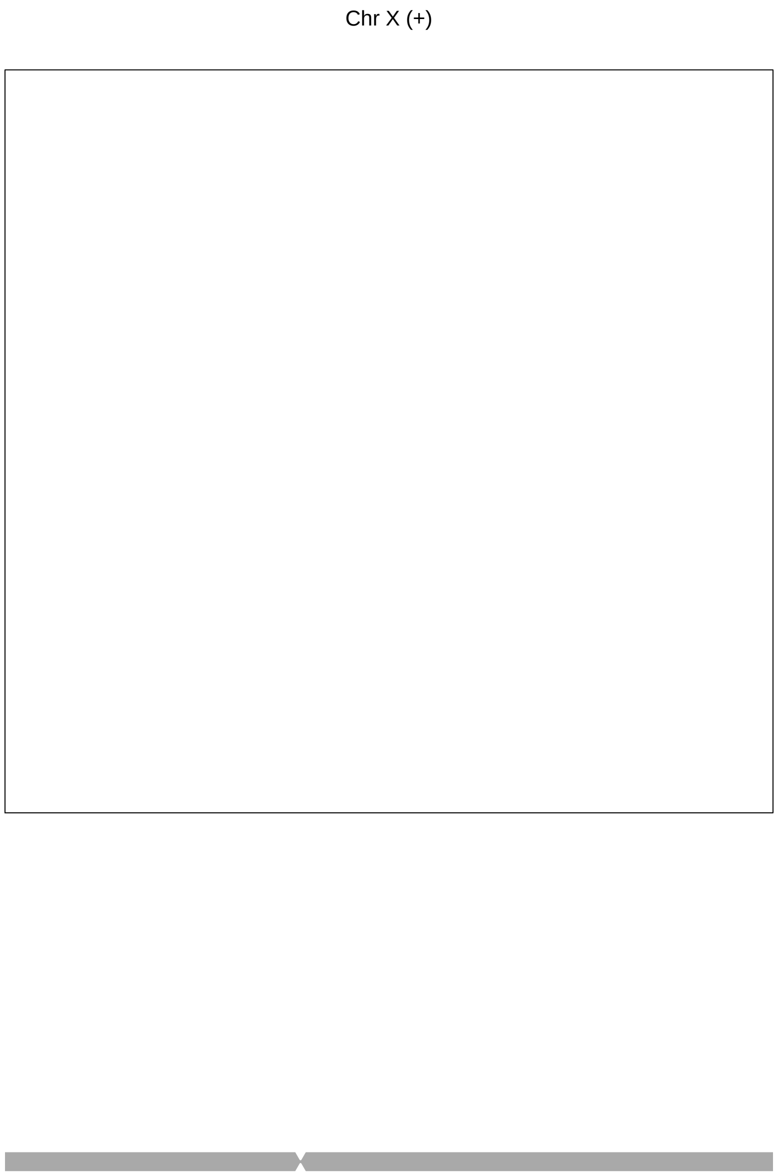
Chr X (+)

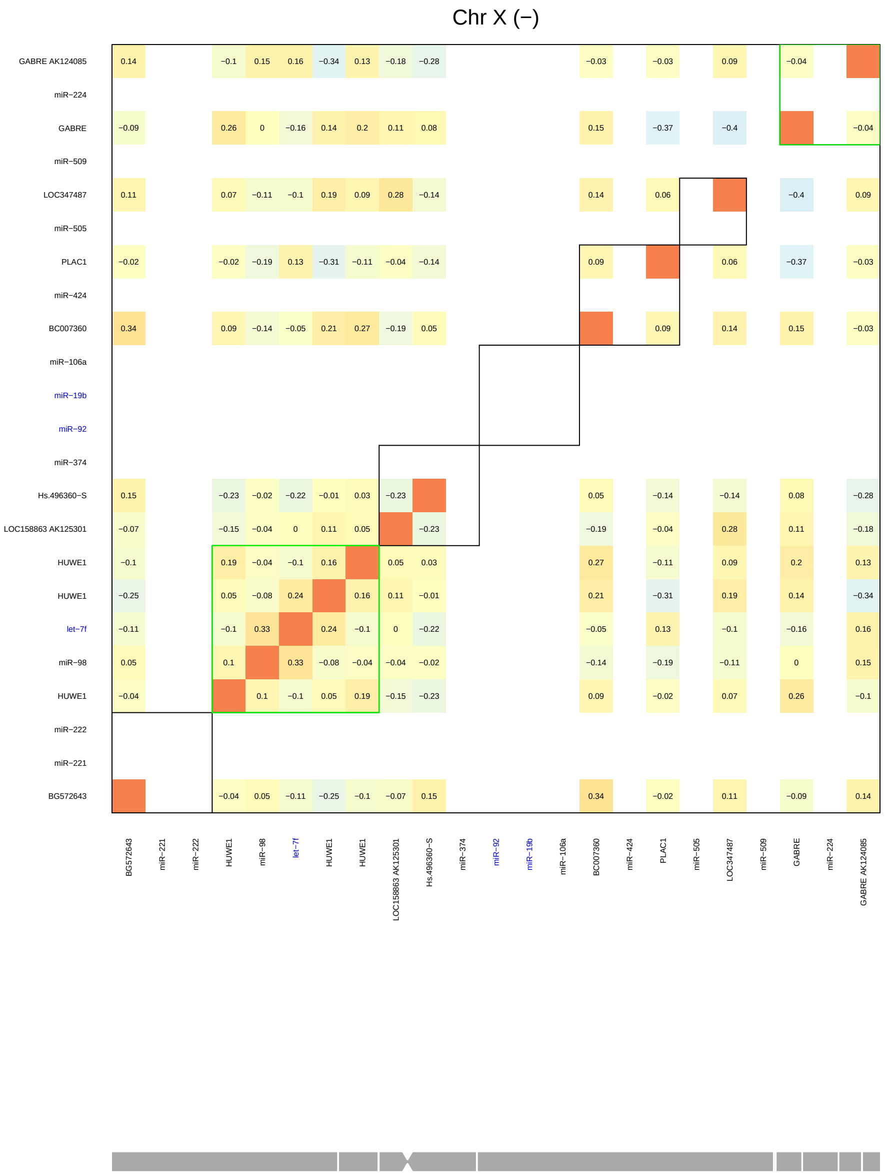
Chr X (−)
GABRE AK124085
miR−224
GABRE
miR−509
LOC347487
miR−505
PLAC1
miR−424
BC007360
miR−106a
miR−19b
miR−92
miR−374
Hs.496360−S
LOC158863 AK125301
HUWE1
HUWE1
let−7f
miR−98
HUWE1
miR−222
miR−221
BG572643
